# Host Genetic Determinants of Hepatitis B Virus Infection

**DOI:** 10.3389/fgene.2019.00696

**Published:** 2019-08-13

**Authors:** Zhenhua Zhang, Changtai Wang, Zhongping Liu, Guizhou Zou, Jun Li, Mengji Lu

**Affiliations:** ^1^Department of Infectious Diseases, the Second Affiliated Hospital of Anhui Medical University, Hefei, China; ^2^College of Pharmacy, Anhui Medical University, Hefei, China; ^3^Department of Infectious Diseases, the Affiliated Anqing Hospital of Anhui Medical University, Anqing, China; ^4^Institute of Virology, University Hospital of Duisburg-Essen, Essen, Germany

**Keywords:** hepatitis B virus, genetic determinants, human leukocyte antigen, susceptibility gene, single nucleotide polymorphism, genome-wide association study

## Abstract

Chronic hepatitis B virus (HBV) infection is still a major health problem worldwide. Recently, a great number of genetic studies based on single nucleotide polymorphisms (SNPs) and genome-wide association studies have been performed to search for host determinants of the development of chronic HBV infection, clinical outcomes, therapeutic efficacy, and responses to hepatitis B vaccines, with a focus on human leukocyte antigens (HLA), cytokine genes, and toll-like receptors. In addition to SNPs, gene insertions/deletions and copy number variants are associated with infection. However, conflicting results have been obtained. In the present review, we summarize the current state of research on host genetic factors and chronic HBV infection, its clinical type, therapies, and hepatitis B vaccine responses and classify published results according to their reliability. The potential roles of host genetic determinants of chronic HBV infection identified in these studies and their clinical significance are discussed. In particular, HLAs were relevant for HBV infection and pathogenesis. Finally, we highlight the need for additional studies with large sample sizes, well-matched study designs, appropriate statistical methods, and validation in multiple populations to improve the treatment of HBV infection.

## Introduction

**Figure 1:**
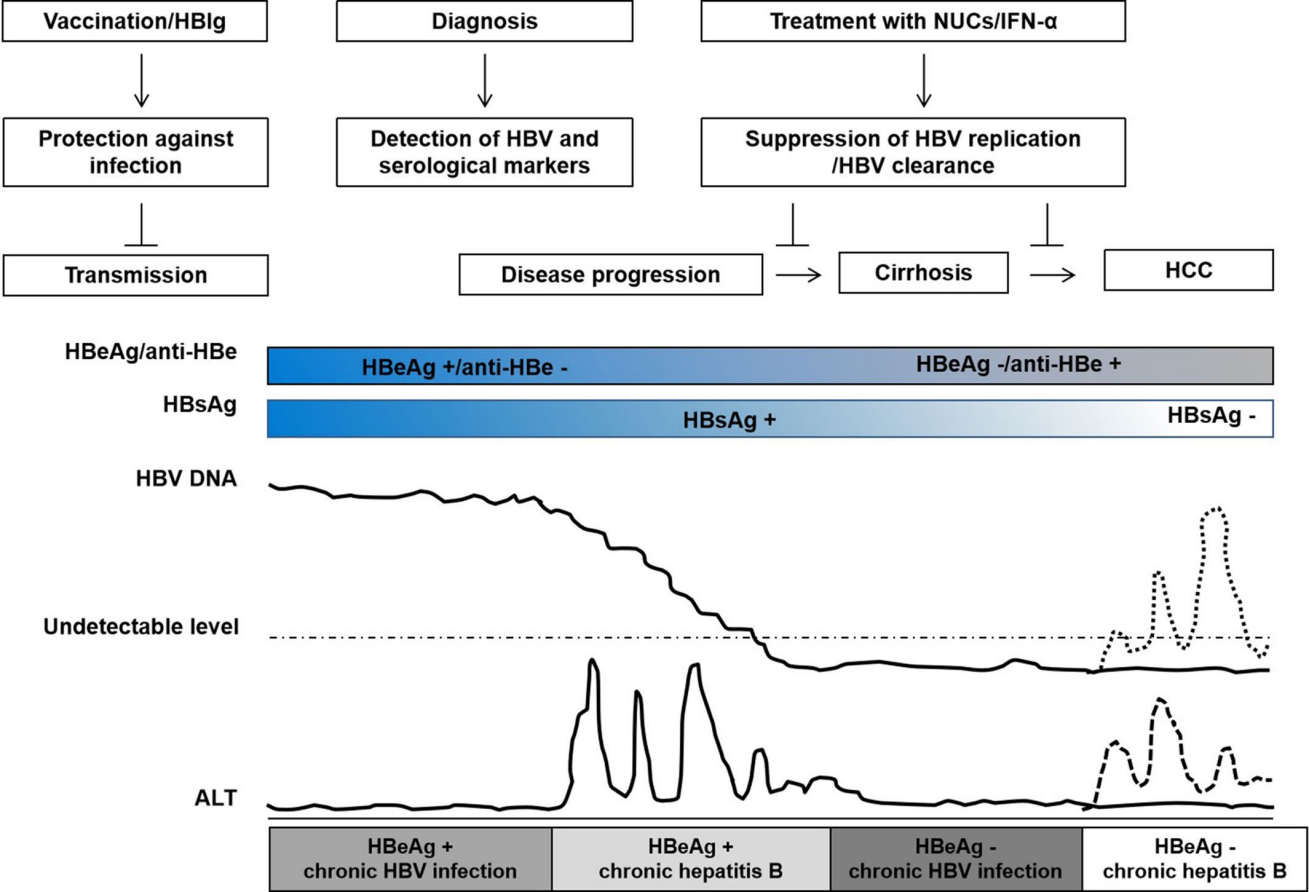
Natural history of chronic hepatitis B virus (HBV) infection, interventions, and clinical outcomes. The natural history of HBV infection is typically classified into four clinical states: HBeAg+ chronic HBV infection, HBeAg+ chronic hepatitis, HBeAg– chronic HBV infection, and HBeAg− chronic hepatitis according to the guideline of European Association for the Study of the Liver (EASL). Patients with chronic HBV infection do not necessarily experience all states. HBV vaccine and/or HBIg injection may prevent HBV transmission; during HBeAg+ chronic hepatitis, treatment with nucleos(t)ide analogs (NUCs) or interferon-alpha (IFN-α) may suppress HBV replication, slow disease progression, and reduce the risk of cirrhosis and hepatocellular carcinoma; HBV reactivation may occur during HBeAg– chronic HBV infection in patients with HBsAg and/or anti-HBc positive after chemotherapy or immunosuppression. The course of HBV infection and disease progression are variable, indicating that host genetic determinants play an important role in HBV infection.

With 292 million people infected and a global prevalence of 3.9%, hepatitis B virus (HBV) infection is still a major global public health problem (Polaris Observatory Collaborators, 2018). The outcomes of HBV infection are highly diverse, including acute hepatitis, self-limiting recovery, chronic hepatitis, cirrhosis, liver cancer, and liver failure (**Figure 1**) (European Association For The Study Of The Liver, 2017). In the natural history of HBV infection, there is substantial variation in the course of disease development and clinical outcomes depending on the transmission pattern, timing of infection, sex, immune status, host genetic factors, and underlying diseases in infected individuals. The disease outcome is related to viral, environmental, and host factors (Ganem and Prince, 2004; Rehermann and Bertoletti, 2015). With respect to host factors, in addition to age, gender, alcohol, obesity, diabetes, and renal failure, host gene variants may also affect the clinical course of HBV infection (Laskus et al., 1992; He et al., 2006; Thursz et al., 2011; Yano et al., 2013; Matsuura et al., 2016). Since the implementation of the Human Genome Project, great progress has been made in genetic and disease-related research, providing a basis for advances in precision medicine. Host genetic polymorphisms mainly include single nucleotide polymorphisms (SNPs), insertions/deletions, and copy number variations (CNVs). Owing to technological limitations, current research mainly focuses on the relationships between SNPs and susceptibility to diseases.

Two approaches are frequently used to screen susceptibility genes for HBV-related diseases and outcomes. First, genome-wide association study (GWAS) have become a common tool for genetic association research focused on complex diseases (Hardy and Singleton, 2009), including HBV infection. In the first stage of GWAS, patient samples are typically used to screen a large number of candidate SNP loci using TaqMan probes, SNPstream genotyping technology, SNaPshot genotyping, SNP chips, and other methods, with several rounds of validation to finally identify SNPs. Second, the traditional approach is the selection of candidate genes predicted to play a role in HBV-related diseases or treatment responses based on theory and previous analyses, followed by verification by comparisons of SNP genotype frequencies in patient and control groups.

**Figure 2:**
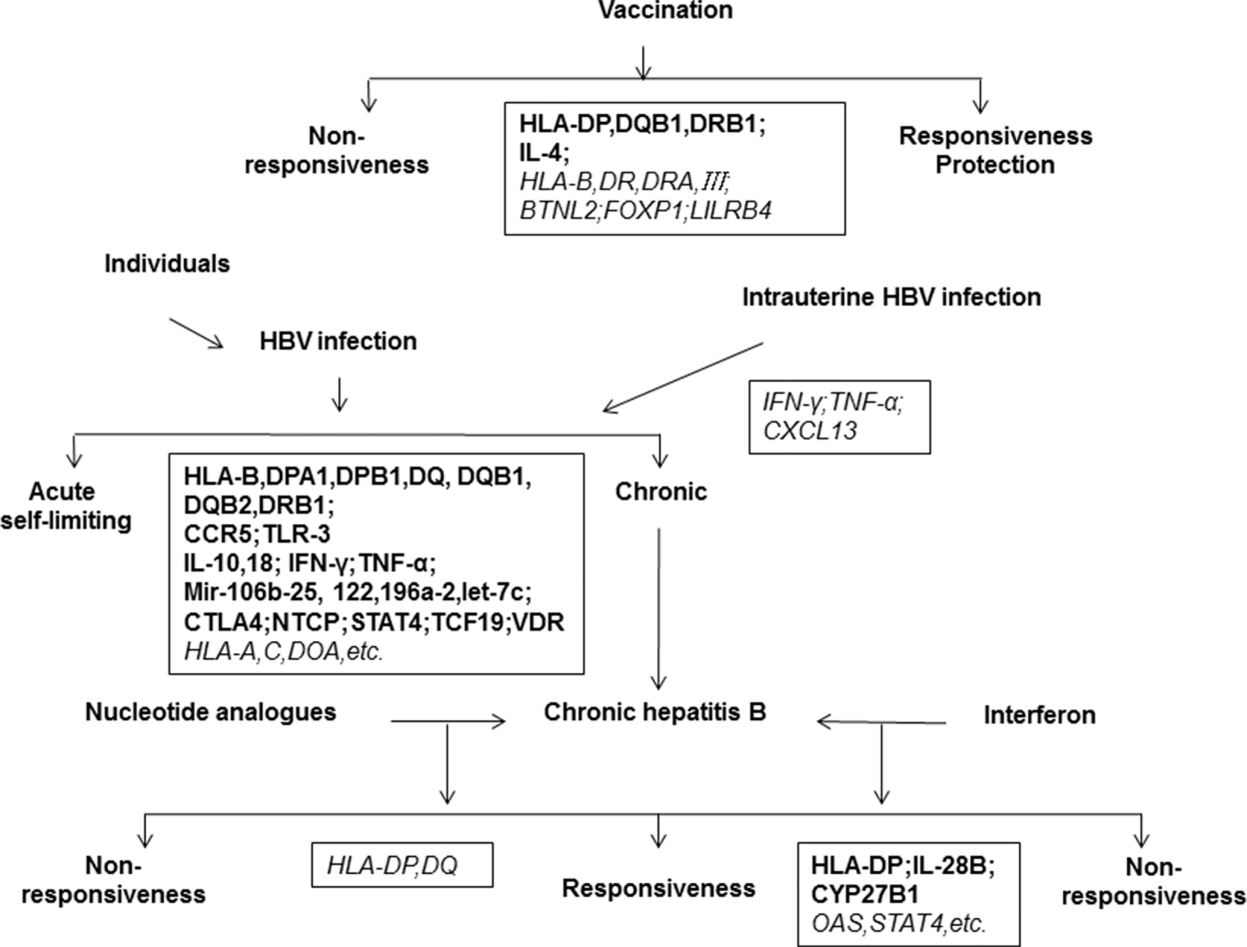
Host genetic determinants of HBV infection. The outcomes of HBV infection are highly diverse, including acute hepatitis, self-limiting recovery, chronic hepatitis, cirrhosis, liver cancer, and liver failure. The disease outcome is related to viral, environmental, and host factors. This map shows important host genes that may be associated with clinical outcomes of HBV infection or Hepatitis B vaccination. Black boxes show the relevant genes identified, in which the bold letter indicates a high degree of correlation and the italic indicates a possible correlation to clinical features of HBV infection.

Many clinical relevant aspects of HBV prevention, infection, and treatment are potentially related to host genetics (Kamatani et al., 2009; Mbarek et al., 2011; Brouwer et al., 2014; Chang et al., 2014; Hosaka et al., 2015; Jiang et al., 2015; Jiang et al., 2016a; Brouwer et al., 2019). Hepatitis B vaccines are widely used and effective for the prevention of new HBV infections. However, the overall success rate of hepatitis B vaccination is about 90% (Bruce et al., 2016; Hu et al., 2018). The outcome of HBV infection can vary dramatically, depending on both host and viral factors. A major part of patients do not experience clinically relevant symptoms during the acute infection phase while others have acute illness with symptoms that last several weeks. Without any intervention, about 95% of intrauterine or perinatal HBV infections but only about 5% in adults develop into chronic infections (Stevens et al., 1975; Wright and Lau, 1993; Petrova and Kamburov, 2010). HBV persistence is a major risk for developing chronic hepatitis B, cirrhosis, hepatocellular carcinoma, or acute liver failure (Wright and Lau, 1993; Zhang et al., 2013a). A small number of patients develop occult HBV infection (OBI) without detectable serum HBsAg but HBV DNA in the serum or liver (Torbenson and Thomas, 2002; Raimondo et al., 2008; Raimondo et al., 2019; Seed et al., 2019). Interferon-alpha (IFN-α) and nucleos(t)ide analogs (NUCs) have been approved and are widely used for the treatment of chronic hepatitis B (European Association For The Study Of The Liver, 2017). Regimens such as antiviral treatment with tenofovir and entecavir provide results in viral suppression in around 95% of patients, have limited associated resistance, and prevent liver disease progression, cirrhosis, and HCC. However, these treatments do not regularly achieve clinical cure or HBsAg clearance and the incidence of cirrhosis or HCC is still significantly higher in treated patients than those without HBV infection (**Figure 1**). Many studies have shown that host genetic polymorphisms may influence HBV infection, including hepatitis B vaccine responses, chronic HBV infection (CHI), intrauterine transmission (IT), OBI, liver cirrhosis (LC), hepatocellular carcinoma (HCC), liver transplantation (LT), and the antiviral efficacy of IFNs and NUCs (Kamatani et al., 2009; Davila et al., 2010; Li et al., 2012b; Chang et al., 2014). These studies have demonstrated that host genetics play an important role in HBV infection and pathogenesis (**Figure 2**). Yet, many contradictory or ambiguous findings have been reported.

In this review, we summarize the current state of research on the role of host genetic determinants of CHI, its clinical type, therapeutic responses, and responses to hepatitis B vaccines (**Tables S2**–**7**). More than 500 publications varying in quality have focused on this topic. Thus, we assessed the reliability of these studies according to the sample size, type of technology, and analysis methods. Note that for great many genes related to HBI or vaccine efficacy, only a single study was available. For intrauterine infections, OBI, drug efficacy, and other factors, all published papers were included owing to the small sample size. We also considered whether there are valid contradictions among studies. We classified studies into three categories: very relevant (I), possibly relevant (II), and unable to confirm (III) (**Table 1**; **Table S1**), as a guide for readers. The topic of host genetics and end-stage liver diseases, including HCC, is reviewed by An et al. (An et al., 2018) in this special issue and therefore is not included in our review.

**Table 1:** Classification of host genetic determinants associated with hepatitis B virus (HBV)-related liver diseases and responses to HBV vaccines.

Category	Relevance	Description
I	High	▲ Meta-analysis suggests that one or more SNP loci are associated with HBV-related diseases. ▲ More than three studies suggest that the same SNP locus is associated with HBV-related diseases.
II	Likely	▲ GWAS suggests that one or more SNP loci are associated with HBV-related diseases. ▲ At least two studies suggest that the same SNP locus is associated with HBV-related diseases. ▲ A single sample with a total sample size of more than 1,000 indicates that SNP loci are associated with disease.
III	Not fully confirmed	▲ A single study with a total sample size of less than 1,000 indicates that SNP loci are associated with HBV-related diseases. ▲ Meta-analysis suggest that there was no correlation, but some studies suggested a correlation with HBV-related diseases. ▲ The majority of studies suggest no correlation with HBV-related diseases. ▲ Single studies with a total sample size of more than 1,000 indicated no correlation with HBV-related diseases. ▲ The total sample size was less than 500 cases. ▲ For the same SNP loci, there are contradictions between different MA or GWAS. ▲ The sample size is greater than 1,000, and the results (the same SNP locus) of studies contradict each other. ▲ Less than two studies failed to show a correlation. ▲ A total sample size of 500–1,000 study subjects, and no correlation is found.

1. The total sample size refers to the sample size of a single study, including the experimental and control group.

2. The study groups of intrauterine infection, OBI, IFN-*α*, and NUCs were changed to 200 and 500 (500 and 1,000 for CHI and HBV vaccine groups, respectively), and the other assessment criteria remain the same.

## Chronic HBV Infection

Early epidemiological data showed that chronic HBV infection is very frequently established in infants born to HBV carrier mothers or in young children (Stevens et al., 1975; Li et al., 2015) and shows a strong gender disparity with significantly higher susceptibility of male patients (Lee et al., 1999; Su et al., 2007; Liaw et al., 2009; Wang et al., 2015). Adequate host immune responses to HBV are essential for sustained viral control (Bertoletti and Gehring, 2006). HBV control is associated with vigorous, multi-specific T cell responses in the host. In chimpanzees, T cell depletion prevents HBV clearance (Thimme et al., 2003; Asabe et al., 2009; Hoogeveen et al., 2018), demonstrating the essential role of cellular immunity in HBV control. Chronic HBV infection is associated with impaired immune responses (Guidotti et al., 2015; Lebosse et al., 2017). Thus, great efforts have been made to identify associations between host immunogenetics and chronic HBV infection. A number of host genetic variants, such as mutations in human leukocyte antigens (HLAs), cytokine and chemokine genes, toll-like receptor (TLRs), microRNAs, vitamin D–related genes, and HBV receptor sodium taurocholate cotransporting polypeptide (NTCP), have been found to influence the outcome of HBV infection (**Table 2**; **Tables S1**, **S2**). The SNPs loci of genes very relevant or possibly relevant with chronic HBV infection are listed in **Table 2**. Other SNPs loci of genes that are still unable to confirm are listed in **Tables S1** and **S2**.

**Table 2:** Host genetic factors associated with chronic HBV infection.

Category	Gene ontology	Gene	Genetic determinants (SNP/Hap/CNVs)	Main reference
I	HLA	HLA-B	*07, *58	Seshasubramanian et al., 2018
		HLA-DPA1	rs3077	Kamatani et al., 2009; Mbarek et al., 2011; Yan et al., 2012b; Jiang et al., 2015
		HLA-DPB1	rs2281388, rs9277535, rs9277542	Kamatani et al., 2009; Mbarek et al., 2011; Nishida et al., 2012; Yan et al., 2012b; Zhang et al., 2013b; Jiang et al., 2015; Trinks et al., 2017
		HLA-DQ	rs9275319, rs9275572	Al-Qahtani et al., 2014; Zhang et al., 2014b; Kim et al., 2015; Xu et al., 2018
		HLA-DQB1	*0201, *0301, *0303, *0502, *0604, rs2856718	Mbarek et al., 2011; Jiang et al., 2015; Huang et al., 2016; Xu et al., 2017b; Xu et al., 2018
		HLA-DQB2	rs7453920	Hu et al., 2013; Jiang et al., 2015; Xu et al., 2017b; Xu et al., 2018
		HLA-DRB1	*13	Zhu et al., 2016b
	Cytokines	IL-10	−592	Cheong et al., 2006; Chen et al., 2010
		IL-18	−137	Karra et al., 2015
		IFN-γ	+874	Sun et al., 2015
		TNF-α	−238, −308, −857, −863	Kim et al., 2003; Cheong et al., 2006; Shi et al., 2012; Zheng et al., 2012
	Chemokines	CCR5	Δ32	Suneetha et al., 2006
	TLRs	TLR-3	rs3775291	Geng et al., 2016
	MicroRNAs	MiR-106b-25	rs999885	Liu et al., 2012b; Zhou et al., 2015
		miR-122	rs3783553	Zhou et al., 2015
		miR-196a-2	rs11614913	Zhou et al., 2015; Al-Qahtani et al., 2017
		miR-let-7c	rs6147150	Zhou et al., 2015
	Others	CTLA-4	rs231775, rs5742909	Schott et al., 2007; Huang et al., 2013; Xu et al., 2013
		NTCP	rs2296651	Hu et al., 2016; Wang et al., 2017a; Nfor et al., 2018
		STAT4	rs7574865	Kim et al., 2015; Jiang et al., 2016b
		TCF19	rs1419881	Kim et al., 2013; Jiang et al., 2015
		VDR	FokI	He et al., 2018
II	HLA	HLA-A	*33:03:01	Miao et al., 2013
		HLA-B	*13:01:01	Miao et al., 2013
		HLA-C	Leu-15, rs2853953, rs3130542	Hu et al., 2013; Jiang et al., 2015; Zhu et al., 2016b
		HLA-DOA	rs378352	Jiang et al., 2015
		HLA-DP	rs9366816	Chang et al., 2014
		HLA-DPA1	rs2301220, rs2395309, rs9277341	Guo et al., 2011; Li et al., 2011a
		HLA-DPB1	rs9277534, *0201, *0401, *0901, positions 84–87, *0402, *0501, *0201-*0401, *0201-*0402, *0201-*0501, *0401-*0402, *0401-*0501, *0402-*0402, *0402-*0501, *0501-*0501, *0501-*0901, *0501*others, rs9277378, rs10484569, rs3117222, rs9380343, rs3135021, G-A-G-A-T-T^1^, G-G-G-G-T-C^2^	Guo et al., 2011; Thomas et al., 2012; Wong et al., 2013; Zhang et al., 2013e; Hu et al., 2014; Nishida et al., 2014; Nishida et al., 2015; Zhu et al., 2016b
		HLA-DQ	A1*0101-B1*0501, A1*0102-B1*0303, A1*0102-B1*0604, A1*0301-B1*0601, A1*0102-B1*0602, A1*0301-B1*0302, A1*0301-B1*0303, A1*0301-B1*0401, A1*0501-B1*0301	Mbarek et al., 2011
		HLA-DQA2	rs9276370	Chang et al., 2014
		HLA-DQB2	rs7756516	Chang et al., 2014
		HLA-DPA1/DPB1	A-A^3^, A-A^4^, T-A-T^5^,C-A-T^6^,A1*0103-B1*0401, A1*0103-B1*0402, A1*0202-B1*0301, A1*0202-B1*0501, A-A-C-T^7^, A-A-C-C//A-G-T-G-C-C^8^, A-A-C-T//A-G-T-G-C-C^9^, G-G-T-C//A-G-T-G-C-C^10^	Kamatani et al., 2009; Guo et al., 2011; Li et al., 2011a; Wang et al., 2011; Wong et al., 2013
		HLA-DP/DQ	T-T-G-A-T^11^, T-T-G-G-T^12^, carrying 4–6 variant alleles, G-A^13^, A-G^14^, A-A^15^	Hu et al., 2012; Chang et al., 2014; Al-Qahtani et al., 2014
		HLA-J	rs400488	Zhu et al., 2016b
	Cytokines	IL-10RB	rs2834167	Romporn et al., 2013; Ma et al., 2014
		IL-12B	rs3212227	He et al., 2015
		IL-16	rs11556218	Li et al., 2011b
		IL-21	rs2221903	Li et al., 2013a
		IFN-α2	p.Ala120Thr	Zhao et al., 2012
		IFN-αR2	rs1051393, rs12233338	Ma et al., 2018a
		IFNLR1	rs4649203, rs7525481	Ma et al., 2018a
		IFN-γR1	rs3799488	He et al., 2015
		IFN-γR2	rs1059293	He et al., 2015
		TNF-α	T-C-C-G-G-G^16^, C-A-C-G-G-G^17^	Kim et al., 2003
		TGF-α	+106151, +103461, A-T-G-T-T-T-T-C-T^18^	Kim et al., 2009
	TLRs	TLR-3	rs1879026	Al-Qahtani et al., 2012a
		TLR-9	rs352140	He et al., 2015
	MicroRNAs	miR-30a	rs1358379	Al-Qahtani et al., 2017
		miR-101-2	rs12375841, T-C^19^	Bae et al., 2012
		miR-122	rs4309483	Liu et al., 2014b
		miR-219-1	rs107822, rs213210, rs421446, C-A-C^20^, T-G-T^21^	Cheong et al., 2013
		miR-323b	rs56103835	Yu et al., 2014
		miR-423	rs6505162	Al-Qahtani et al., 2017
		miR-492	rs2289030	Al-Qahtani et al., 2017
	Others	C2	p.Glu318Asp, rs7746553, rs9267673, rs9267665, rs9267677, rs9279450, rs10947223	Zhao et al., 2012; Namgoong et al., 2018
		CD40	rs1883832	Jiang et al., 2015
		CFB	rs12614	Jiang et al., 2015
		CTLA-4	rs3087243, C-A-C-C-G^22^, T/C-A-C-C-G^23^, T-A-C-C-A^24^	Thio et al., 2004; Chen et al., 2010
		EHMT2	rs7887, rs652888, rs35875104, rs41267090	Jiang et al., 2015; Shin et al., 2017
		ESR1	+29	Deng et al., 2004
		IFN4/HLA-DQ	rs12971396-rs9275319, rs12971396-rs12979860-rs9275319	Fan et al., 2016a
		INST10	rs7000921	Li et al., 2016
		KIF1B	A-T-A^25^	Al-Qahtani et al., 2012b
		MCP1	−2518, −2518/−2076(−/ht2), −2518/−2076(ht2/ht2)	Park et al., 2006; Chen et al., 2010
		MIF	rs755622	Moudi et al., 2016
		MxA	−123	Cao et al., 2009; Rebbani et al., 2017
		MX1	rs467960	He et al., 2015
		NF-κB	rs2233406, rs3138053	Zhang et al., 2014a
		NLRX1	p.Arg707Cys	Zhao et al., 2012
		NOTCH4	rs422951	Jiang et al., 2015
		NTCP	rs943277, rs4646285	Wang et al., 2017a; Wu et al., 2018a
		OCT4	rs13409, rs885952, rs879882, rs1265163, rs2394882, rs3094193, rs3130501, rs3130503, rs3130931, rs3132526, rs3757349, rs9263800, rs117265349	Shin et al., 2017
		PAPL	rs423058	Ma et al., 2014
		SOCS3	rs111033850, rs12953258, C-C ^26^	Hoan et al., 2017
		SPP1	−1800, T-T-C-T-A^27^	Shin et al., 2007
		STAT4	rs8179673, rs10168266, rs11889341, C-T-C-T-T^28^	Jiang et al., 2016b, Lu et al., 2015b
		TMEM2	p.Ser1254Asn	Zhao et al., 2012
		TMEM2/IFNA2/NLRX1/C2	p.Ser1254-Asn/p.Ala120Thr/p.Arg707Cys/p.Glu318Asp	Zhao et al., 2012
		UBE2L3	rs2266959, rs4821116	Liu et al., 2018d, Hu et al., 2013
		VARS2	rs1043483, rs1264295, rs2249464, rs2517459, rs2532932, rs9394021,	Cheong et al., 2015
		ZNRD1	rs3757328, G-G-A^29^	Wen et al., 2015

**Haplotypes**

^1^G-A-G-A-T-T, rs9277535-rs10484569-rs3128917-rs2281388-rs3117222-rs9380343; ^2^G-G-G-G-T-C, rs9277535-rs10484569-rs3128917-rs2281388-rs3117222-rs9380343; ^3^A-A, rs3077-rs9277535; ^4^A-A, rs2395309-rs9277535; ^5^T-A-T, rs3077-rs9277378-rs3128917; ^6^C-A-T, rs3077-rs9277378-rs3128917; ^7^A-A-C-T, rs2395309-rs3077-rs2301220-rs9277341; ^8^A-A-C-C//A-G-T-G-C-C, rs2395309-rs3077-rs2301220-rs9277341//rs9277535-rs10484569-rs3128917 -rs2281388-rs3117222-rs9380343; ^9^A-A-C-T//A-G-T-G-C-C, rs2395309-rs3077-rs2301220-rs9277341//rs9277535-rs10484569-rs3128917 -rs2281388-rs3117222-rs9380343; ^10^G-G-T-C//A-G-T-G-C-C, rs2395309-rs3077-rs2301220-rs9277341//rs9277535-rs10484569-rs3128917 -rs2281388-rs3117222-rs9380343; ^11^T-T-G-A-T, rs9276370-rs7756516-rs7453920-rs9277535-rs9366816; ^12^T-T-G-G-T, rs9276370-rs7756516-rs7453920-rs9277535-rs9366816; ^13^G-A, rs2856718- rs9275572; ^14^A-G, rs2856718- rs9275572; ^15^A-A, rs2856718- rs9275572; ^16^T-C-C-G-G-G, -1031/-863/-857/-308/-238/-163; ^17^C-A-C-G-G-G, -1031/-863/-857/-308/-238/-163; ^18^A-T-G-T-T-T-T-C-T, +88344/+102906/+103432/+103437/+103461/+104261/+104802/+106151/+106318; ^19^T-C, rs12375841-rs17803780; ^20^C-A-C, rs421446-rs107822-rs213210; ^21^T-G-T, rs421446-rs107822-rs213210; ^22^C-A-C-C-G, -1722/-1661/-658/-319/+49; ^23^T/C-A-C-C-G, -1722/-1661/-658/-319/+49; ^24^T-A-C-C-A, -1722/-1661/-658/-319/+49; ^25^A-T-A, rs17401966-rs12734551-rs3748578; ^26^C-C, rs111033850-rs12953258; ^27^T-T-C-T-A, -1800/-1627/+4645/+5806/+6139; ^28^C-T-C-T-T, rs8179673-rs7574865-rs4274624-rs11889341-rs10168266; ^29^G-G-A, rs3757328-rs6940552-rs9261204.

HLA is the human major histocompatibility complex (MHC) and a central element of antiviral immune defense (Shiina et al., 2009). HLA genes are classified as class I (HLA-A, B, C, E, F, and G) and II (HLA-DP, DQ, DR, DM, and DO) (Koller et al., 1989; van Lith et al., 2010). When a pathogen enters the body, it is engulfed by antigen-presenting cells (APCs) and the pathogen proteins are digested into small pieces and loaded onto HLA antigens. They are then displayed by APCs to T cells, which produce a variety of effector molecules to eliminate the pathogen. Therefore, HLAs can affect the outcome of infectious diseases (Singh et al., 2007; Goulder and Walker, 2012; Wang et al., 2016). Genetic polymorphisms, especially in HLA class II genes, are significantly associated with HBV infection and pathogenesis according to many studies (Kamatani et al., 2009; Zhang et al., 2014b; He et al., 2015; Wasityastuti et al., 2016; Trinks et al., 2017). Kamatani et al. (Kamatani et al., 2009) performed a GWAS of chronic HBV infection using 786 cases and 2,201 HBsAg seronegative controls in the Japanese population, followed by a replication study of three additional Japanese and Thai cohorts consisting of 1,300 cases and 2,100 controls. HLA-DPA1 rs3077 and HLA-DPB1 rs9277535 were significantly associated with chronic HBV infection in the Asian population (combined OR [95% CI] = 0.56 [0.51–0.61], *P* = 2.31 × 10^–38^; OR [95% CI] = 0.57 [0.52–0.62], *P* = 6.34 × 10^–39^, respectively). They also identified four associated haplotypes: HLA-DPA1*0202-DPB1*0501 and HLA-DPA1*0202-DPB1*0301 were associated with susceptibility to chronic hepatitis B and HLA-DPA1*0103-DPB1*0402 and HLA-DPA1*0103-DPB1*0401 showed a protective effect. Several GWAS of Asian populations have also shown that SNPs in HLA regions, such as HLA-DP (rs9277535, rs3077, rs9366816, and rs9277542), HLA-DQ (rs2856718, rs7453920, rs9276370, rs7756516, and rs7453920), HLA-DPB1 (positions 84–87), HLA-C (Leu-15, rs3130542, and rs2853953), HLA-DRB1*13, HLA-J (rs400488), and HLA-DOA (rs378352), are associated with chronic HBV infection (Mbarek et al., 2011; Nishida et al., 2012; Hu et al., 2013; Kim et al., 2013; Chang et al., 2014; Jiang et al., 2015; Zhu et al., 2016b). Many studies have provided evidence based on case–control samples and conventional PCR-based detection methods that HLA molecules may be crucial determinants of the outcomes of HBV infection in Asian, European, African-American, Saudi Arabian, and Caucasian populations (Thomas et al., 2012; Al-Qahtani et al., 2014; Trinks et al., 2017). However, some studies have reported contradictory results. A study of the Chinese Zhuang population found no association between HLA-DPA1 rs3077 and chronic HBV infection (Wang et al., 2011). Akgöllü et al. (Akgöllü et al., 2017) suggested that HLA-DP rs3077 is not associated with HBV infection in the Turkish population. Vermehren et al. (Vermehren et al., 2012) failed to confirm an association of HLA-DPB1 rs9277535 with hepatitis B in Caucasians. A comprehensive meta-analysis suggested that HLA-DP/DQ (rs3077, rs9277535, rs9275572, rs9275319, rs2856718, and rs7453920) is associated with susceptibility to HBV or the clearance of HBV infection (Zhang et al., 2013e; Yu et al., 2015; Xu et al., 2017b; Xu et al., 2018). A meta-analysis by Huang et al. (Huang et al., 2016) suggested that HLA-DQB1*0201, DQB1*0301, and DQB1*0502 are associated with an increased risk of CHB (chronic hepatitis B), while HLA-DQB1*0303 and DQB1*0604 are associated with a decreased risk of CHB.

Another meta-analysis indicated that HLA B*07 and B*58 protect against chronic HBV infection (Seshasubramanian et al., 2018). Recent studies have shown that non-classical HLA-class I molecules, including HLA-E may also be related to hepatitis B virus infection (Zidi et al., 2016). Available data are consistent with the fact that cellular immunity is a major determinant for HBV control. However, it is not clear how the identified genetic variation influences immune functions in the host and specific immune responses to HBV. These critical questions need to be answered in future studies.

Cytokines are key antiviral and immunomodulatory molecules produced by immune cells and certain non-immune cells; they are involved in host defense against HBV infection and pathogenesis (Koziel, 1999; Shin et al., 2016). The antiviral and immunomodulatory functions of a number of cytokines, like type I and II IFNs, TNF-α, IL-10, and IL-21, play essential roles in the direct suppression of HBV replication in hepatocytes (Phillips et al., 2010), mediating the antiviral functions of T cells (Freeman et al., 2012), and the modulation of adaptive immune responses to HBV (Lin and Young, 2014; Gehring and Protzer, 2019). IFN-γ and TNF-α are major antiviral mediators of specific CD8+T cells and are required for HBV control in primary HBV infections (Phillips et al., 2010; Bertoletti and Ferrari, 2013). IL-10 may contribute to negative regulation of host immune responses and thereby plays a role in viral persistence (Saraiva and O’Garra, 2010). IL-18 is a cytokine that belongs to the IL-1 superfamily and is produced by macrophages and other cells (Mavragani and Moutsopoulos, 2014). Recently, IL-21 has drawn attention in the field of HBV research owing to its potential association with viral control *via* follicular helper T cell functions (Johnson and Jameson, 2009). A series of studies have identified associations between genes encoding cytokines (including IL-1, IL-4, IL-6, IL-10/IL-10RB, IL-12/IL-12B, IL-18, IL-27, IL-28B, IFN-γ, TNF-α, and TGF-β) and chronic HBV infection (Cheong et al., 2006; Du et al., 2006; Liu et al., 2006; Chen et al., 2010; Fletcher et al., 2011; Ma et al., 2014; Saxena et al., 2014; Talaat et al., 2014; He et al., 2015; Karataylı et al., 2015; Karra et al., 2015; Eskandari et al., 2017b). Clinical researches have revealed correlations between IL-18 -607C/A and -137G/C (rs1946519 and rs187238) polymorphisms and the risk of HBV infection (Karra et al., 2015). The study of a Indian population by Karra et al. involving 271 patients with hepatitis B–related liver diseases, including 109 with spontaneous recovery, 162 patients with persistent HBV infection, and 280 healthy volunteers, indicated that the −607A allele in the promoter region of the IL-18 gene may protect against HBV infection (healthy controls *vs*. cases, OR [95% CI] = 0.711 [0.559–0.904], *P* = 0.005). The AA genotype was associated with spontaneous clearance (spontaneous recovery *vs*. persistent HBV infection, OR [95% CI] = 2.765 [1.582–4.832], *P* = 0.0002), while the CC genotype was associated with HBV infection (persistent HBV infection *vs*. spontaneous recovery, OR [95% CI] = 0.318 [0.151–0.67], *P* = 0.001). Moreover, the -137C allele (cases *vs*. healthy controls, OR [95% CI] = 1.355 [1.037–1.771], *P* = 0.025) and the GC genotype (cases *vs*. healthy controls, OR [95% CI] = 1.558 [1.11–2.185], *P* = 0.01) were associated with persistent HBV infections. SNPs (−1,082, −592) within the IL-10 gene have been reported to be associated with susceptibility or the clearance of HBV infection (Cheong et al., 2006; Talaat et al., 2014). Further studies are needed to determine the functions of these cytokines in HBV infection and thereby to explain these conflicting results.

IL-28B SNPs are major determinants of HCV clearance and the efficacy of IFN therapy (Ge et al., 2009; Prokunina-Olsson et al., 2013). This locus encodes a cytokine that is distantly related to type I interferons and the IL-10 family and is induced by viral infection (Sheppard et al., 2003). A systematic review by Lee et al. (Lee et al., 2014) including 4,028 patients with chronic hepatitis B and 2,327 spontaneously recovered controls from 11 case–control studies indicated that there is no significant association between IL28B SNPs (rs12979860, rs12980275, and rs8099917) and spontaneous HBV clearance. These findings are not surprising, as the mechanisms underlying HBV control may differ substantially from those underlying HCV infection.

Interferon gamma (IFN-γ) is a type II interferon and is critical for innate and adaptive immunity against viral, bacterial, and protozoal infections (Schoenborn and Wilson, 2007). Cellular responses to IFN-γ are activated by its interaction with a heterodimeric receptor consisting of IFN-γ receptor 1 (IFN-γR1) and IFN-γ receptor 2 (IFN-γR2) (Robek et al., 2004). Previous studies have reported that several polymorphisms in IFN-γ, IFN-γR1, and IFN-γR2 are associated with the natural history of HBV infection (Liu et al., 2006; Khanizadeh et al., 2012). In a study by Liu et al. (Liu et al., 2006) including 181 patients with HBV infection and 272 gender, age-matched healthy controls, the A allele frequency of IFN-γ +874 (OR [95% CI] = 2.25 [1.69–2.99], *P* < 0.0001), and the AG haplotype (+874A and +2109G) (*P* < 0.0001) were found to significantly influence susceptibility to HBV infection in the Chinese population. A meta-analysis by Sun et al. (Sun et al., 2015) suggested that the IFN-γ +874T/A polymorphism contributes to an increased risk of HBV-related diseases, especially in Asians.

Chemokines are a family of small cytokines found in all vertebrates, some viruses, and some bacteria; they guide cells of the innate and adaptive immune systems. Some chemokines are involved in the control of immune cells during immune surveillance, directing lymphocytes to lymph nodes, to screen for pathogen invasion *via* interactions with APCs residing in these tissues (Kehrl, 2006; Mélik-Parsadaniantz and Rostène, 2008).

To date, studies of cytokine and chemokine genes have evaluated limited numbers of SNPs with small sample sizes, and rather inconsistent results have been obtained. Therefore, future large-scale and multi-center studies are needed to establish the relationship between the genetic control of cytokine- and chemokine-related functions and chronic HBV infection, given the importance of cytokines in anti-HBV immune responses.

TLRs play key roles as pattern recognition receptors; they activate the innate immune system (Chang, 2010; Kondo et al., 2011) and are required for efficient host defense against viral infection. Deficiencies in TLR or TLR-related signaling greatly reduce host-specific T cell responses to HBV and may contribute to HBV persistence (Ma et al., 2017). Thus, genetic variation in TLRs is expected to impact the susceptibility to chronic HBV infection (Al-Qahtani et al., 2012a; He et al., 2015; Huang et al., 2015; Zhu et al., 2017). Multiple SNPs in TLR genes (TLR3 rs1879026, rs3775290, and rs3775291, TLR7 rs179010, and TLR9 rs352140) have been examined for their association with the risk of HBV infection (He et al., 2015; Huang et al., 2015; Zhu et al., 2017). SNPs in TLR3 may be associated with an increased risk of chronic HBV infection. Al-Qahtani et al. (Al-Qahtani et al., 2012a) investigated SNPs in the TLR3 gene in Saudi Arabian patients chronically infected with HBV, including 707 patients and 600 uninfected controls. Only TLR3 rs1879026 (OR [95% CI] = 0.809 [0.655–0.999], *P* = 0.0480) and the GCGA haplotype (rs1879026, rs5743313, rs5743314, and rs5743315) (*P* = 0.0339) potentially contribute to the risk of HBV infection. Huang et al. (Huang et al., 2015) indicated that the TT genotype of TLR3 rs3775290 is closely correlated with a decreased risk of CHB. A meta-analysis by Geng et al. (Geng et al., 2016) demonstrated a significant effect of TLR3 rs3775291 and HBV-related diseases.

MicroRNAs (miRNAs) are a group of endogenous, highly conserved, small noncoding RNAs that modulate various cellular processes and play key roles in host–virus interactions and the pathogenesis of viral diseases (Qureshi et al., 2014). HBV replication is also regulated by miRNAs *via* their cellular targets (Zhang et al., 2011a; Zhang et al., 2013d; Lin et al., 2017). Hundreds of SNPs in miRNAs have been reported. Recent studies have shown that some miRNAs may play a role in anti-HBV defense (Su et al., 2011). In addition, miRNA polymorphisms are related to hepatitis B infection (Bae et al., 2012; Cheong et al., 2013; Al-Qahtani et al., 2017). A study of a Saudi Arabian population including 1,352 HBV-infected patients and 600 uninfected healthy individuals found that SNPs in different microRNA genes, including miR-149 (rs2292832), miR-146a (rs2910164), miR-196a-2 (rs11614913), and miR-30a (rs1358379), were associated with hepatitis B infection, while other SNPs in microRNA genes, including miR-423 (rs6505162), miR-492 (rs2289030), miR-146a (rs2910164), miR-196a-2 (rs11614913), and miR-30a (rs1358379) were associated with HBV clearance (Al-Qahtani et al., 2017). A meta-analysis (Zhou et al., 2015) has shown that the carriers of miR-196a-2*T (rs11614913), miR-122*del (rs3783553), miR-106b-25*A (rs999885), and miR-let-7c*del (rs6147150) alleles in the Asian population have an increased risk of chronic HBV infection.

Cytotoxic T-lymphocyte-associated protein 4 (CTLA4) is expressed by T lymphocytes and plays a key role as a negative regulator of T-cell responses (Buchbinder and Hodi, 2015). Several studies have evaluated the association between CTLA4 polymorphisms and chronic HBV infection. In particular, the + 49A/G polymorphism (rs231775) of CTLA4 may influence susceptibility to HBV infection in the Chinese population (Chen et al., 2010). Mohammad et al. (Mohammad et al., 2006) found that CTLA-4 -318 polymorphisms (rs5742909), but not +49 and −1,172 polymorphisms (rs733618) are significantly associated with susceptibility to chronic HBV infection in the Iranian population (OR = 0.49, 95% = 0.206–1.162, *P* = 0.012). Another study also showed that the CTLA4 –318C > T, but not +49G > A, is associated with chronic HBV infection (Schott et al., 2007). A meta-analysis (Xu et al., 2013) suggested that the A allele of the CTLA4 +49 polymorphism is significantly associated with an increased risk of persistent HBV infection, whereas the G allele may influence viral clearance. Another meta-analysis also suggested that the CTLA-4 +49A/G polymorphism is significantly correlated with persistent HBV infection in the Asian population (Huang et al., 2013).

NTCP encoded by the solute carrier family 10 member 1 (SLC10A1) gene is mainly expressed in hepatocytes and a functional receptor for HBV and hepatitis D virus (Hagenbuch and Meier, 1994; Yan et al., 2012a). NTCP rs2296651 (S267F) has been found to be related to HBV susceptibility in the Asian population (Hu et al., 2016; Yang et al., 2016a; Nfor et al., 2018; Wu et al., 2018a). In a larger cohort of Taiwanese patients, including 3,801 with chronic HBV infection and 3,801 matched HBsAg seronegative controls, the AA genotype of the S267F variant (rs2296651) was associated with resistance to chronic HBV infection (OR = 0.13, 95% CI = 0.05–0.34, *P* < 0.001), but there were no significant associations with serological outcomes, including HBV DNA detectability and HBeAg and HBsAg seroclearance (Hu et al., 2016). However, the S267F variant is absent in the Moroccan population (Ezzikouri et al., 2017). A large cohort study did not confirm the association between the common and rare alleles or CNVs in the SLC10A1 gene with the risk of persistent HBV infection in a population from Southern China (Zhang et al., 2017). A meta-analysis including 14,591 chronically HBV-infected patients and 12,396 healthy controls suggested that the A allele and GA genotypes of rs2296651 are inversely correlated with chronic HBV infection. They also reported that NTCP rs4646287, rs7154439, and rs4646296 show no significant correlation with HBV infection (Wang et al., 2017a).

The signal transducer and activator of transcription (STAT) protein family mediates many aspects of cellular immunity, proliferation, apoptosis, and differentiation (Miklossy et al., 2013). STAT proteins are involved in the development and function of the immune system and play a role in maintaining immune tolerance (Singh et al., 2009). In HBV infection, polymorphisms in STAT4 are related to the clinical outcome of HBV infection (Lu et al., 2015b; Jiang et al., 2016b). A case–control study including 1,610 Chinese patients with chronic HBV infection and 1,423 uninfected control subjects showed that the STAT4 SNPs rs7574865, rs10168266, rs11889341, and rs8179673 are significantly associated with the risk of HBV infection and inversely related to HBV clearance. They also found that the CTCTT haplotype, formed by the SNPs rs8179673, rs7574865, rs4274624, rs11889341, and rs10168266, is related to HBV infection susceptibility and clearance in the Chinese Han population. A meta-analysis including 8,944 chronically HBV-infected patients, 8,451 healthy individuals, and 2,081 subjects with HBV clearance demonstrated that STAT4 rs7574865 is significantly associated with HBV infection (OR [95% CI] = 1.14 [1.07–1.21]; *P* = 3.8 × 10^−5^) and clearance (OR [95% CI] = 1.20 [1.07–1.35], *P* = 0.002) (Jiang et al., 2016b). Lu et al. (Lu et al., 2015b) showed that STAT4 minor alleles (rs7574865, rs7582694, rs11889341, and rs8179673) are associated with spontaneous HBV clearance. Another meta-analysis by Liao et al. (Liao et al., 2014) did not confirm the correlation between STAT4 rs7574865 and HBV susceptibility or clearance. Thus, the association of STAT4 polymorphisms with HBV infection requires further verification.

Signal transducer and activator of transcription 3 (STAT3) and nuclear factor-kappaB (NF-κB) pathways may play a significant role in chronic HBV infection. A Chinese Han population study showed that STAT3 rs1053004 and rs1053005 polymorphisms and haplotypes formed by rs1053004 and rs1053005 might contribute to the susceptibility to chronic HBV infection (Li et al., 2018). Another Chinese study also found that the SNPs rs2233406 (CT versus CC) and rs3138053 (AG or AG+GG versus AA) in the promoter region of NFKBIA were significantly associated with decreased risk of HBV persistence, especially in genotype B HBV-infected subjects (Zhang et al., 2014a). Unfortunately, related research is still not sufficient and mainly focused on HCC. Their role of HBV infection needs to be better defined by future research.

Using a GWAS approach, several loci that are strongly associated with HBV clearance or susceptibility to chronic HBV infection have been identified, including TCF19 (Kim et al., 2013; Jiang et al., 2015), UBE2L3 (Hu et al., 2013; Jiang et al., 2015), CFB (Jiang et al., 2015), NOTCH4 (Jiang et al., 2015), CD40 (Jiang et al., 2015), EHMT2 (Kim et al., 2013; Jiang et al., 2015; Shin et al., 2019), and INTS10 (Li et al., 2016).

Vitamin D has the ability to activate the innate immune system and dampen the adaptive immune system (Hewison, 2011). A vitamin D deficiency is associated with an increased risk or severity of viral infections (Beard et al., 2011). Vitamin D has both anti-inflammatory and anti-microbial effects and may play an active role in HBV infection (Bellamy et al., 1999; Beard et al., 2011; Gao et al., 2017). Vitamin D–related genes include 1-α-hydroxylase (CYP27B1), cytochrome P450 family 2 subfamily R polypeptide 1 (CYP2R1), vitamin D–binding protein (GC), VDR, 7-dehydrocholesterol reductase (DHCR7), sterol 27-hydroxylase (CYP27A1), and cytochrome P450 family 24 subfamily A member 1 (CYP24A1). Vitamin D–related genes have recently been found to play an important role in HBV susceptibility (Chatzidaki et al., 2012; Gao et al., 2017). The majority of these studies have focused on four SNPs in VDR: ApaI (rs7975232), TaqI (rs731236), FokI (rs10735810), and BsmI (rs1544410). Chatzidaki et al. (Chatzidaki et al., 2012) found that the joint haplotype of the VDR ApaI α allele and TaqI T allele is related to the outcome of HBV infection in children in the context of mother-to-child transmission (RR [95% CI] = 1.74 [0.97–3.13], *P* = 0.049). A meta-analysis (He et al., 2018) has reported that the genotypes FF, Ff, and allele F of FokI in VDR increase the risk of HBV infection. However, no associations were found between VDR ApaI and BsmI polymorphisms and HBV infection.

There is a marked difference in HBV infection rates between populations in East/Southeast Asia and Western world. For instance, the prevalence of HBsAg was as high as 10% in the Chinese population prior to the initiation of the HBV vaccination program but less than 1% in the Caucasian population (Fattovich et al., 2008; Yin et al., 2010). The reason for this difference is not completely clear. The role of host genetic determinants is also not well-established. Li et al. (Li et al., 2015) summarized multiple studies and found that some genetic loci with rare alleles include rs3138053 (NFKBIA), rs2856718, rs7453920, and rs9275319 (HLA-DQ), and rs9277378, rs2395309, rs2301220, and rs9277341 (HLA-DP) are significantly associated with decreased risks of development of chronic HBV infection. These loci are different between the Chinese Han and European populations, indicating that the Chinese Han patients may be inherently more prone to develop chronic infection once exposed to HBV, whereas Europeans tend to recover from HBV infection spontaneously (Li et al., 2015). A study including Chinese Han and Uyghur populations reported that there are some differences in HLA-DP/DQ polymorphisms that affect susceptibility and resistance to HBV infection (Xiang et al., 2016). A meta-analysis by Geng et al. (2016) demonstrated that TNF-α-238 increases the risk in the European population but not in an Asian population in all genetic models. Therefore, the genetic determinants may contribute the different rates of chronic HBV infection in Asian and European populations. However, future global multicenter research is needed to precisely define such host genetic determinants.

HBV infection often occurs in families. If a mother is chronically HBV infected, infants have an extremely high chance of establishing a chronic HBV infection in the absence of medical interventions (Stevens et al., 1975; Chen et al., 2018; Dionne-Odom et al., 2018). HBV exposure during infancy or early childhood is more prone to the development of chronic infection and HBV infection-related diseases (Lai and Yuen, 2007). Studies have shown that spontaneous HBsAg clearance is most likely to occur in patients born to mothers of non-HBsAg carriers (Chiu et al., 2014). Globally, a total of 42.1% of infants naturally born to HBsAg carrier mothers but only 2.9% of infants with HBV passive–active immunoprophylaxis acquired HBV infection perinatally (Li et al., 2015). The rate of vertical HBV infection had stayed constant (approximately 0.3%) until immunoprophylaxis of mother-to-infant transmission was implemented in 1986 in Japan. In contrast, horizontal HBV transmission decreased from 1.43 to 0.10% in men and from 0.95 to 0.03% in women over years. The decrease of horizontal transmission of HBV may be due to many factors, including improved socioeconomic environments, advanced medical maneuvers and equipment, and careful vaccination procedures (Sato et al., 2014). However, the current studies on host genetic and chronic HBV infection did not separate vertical from horizontal transmission, nor did they stratify the age at the time of infection. The role of environmental exposure including early exposure of HBV in the childhood when immune function is immature, and their interaction with the genetic predisposition is still not clarified.

In studies of associations between host genetic determinants and chronic HBV infection to date, reported OR values for significant associations typically range from 1 to 2. Thus, these determinants have much less influence than the HBV infection status of the mother or the age of the HBV infection. Nevertheless, some genetic variants, like mutations in NTCP, may play a decisive role in HBV infection. NTCP variation could prevent the binding of the HBV virion to receptors on hepatocytes and therefore limit viral infection (Yan et al., 2012a). Such findings may provide mechanistic insights into the viral life cycle and new targets for drug development. Many genetic determinants (HLA, TLR, IL) may modulate host innate and adaptive immunity and significantly influence the course and outcome HBV infection (Kamatani et al., 2009; Mbarek et al., 2011; Kim et al., 2013; Jiang et al., 2015; Geng et al., 2016) (**Table 2**; **Table S2**). It will be useful to identify the functional relevance of these genetic polymorphisms in cellular signaling and other processes, with the goal of identifying new targets. Many factors contribute to the development of chronic HBV infection, such as the mode of transmission (vertical or horizontal transmission), age at the time of infection, the presence of underlying diseases involving other organs, and the use of immune inhibitors (Pawlowska et al., 2016; Chien et al., 2019). The majority of studies do not including subgroup analyses of these factors, which may have a significant impact on the reliability of the results. Furthermore, there are substantial variations among studies from different countries and regions due to differences in the study populations, genetic background, and polymorphisms. Therefore, it may be necessary to conduct stratified analyses of HBV infection time, ethnicity, host immune status, and mode of transmission to further determine the impact of HLA-DP, DQ, and DR on chronic hepatitis B or clearance. Nevertheless, the available information suggests that genetic variation in host immune-related genes has substantial contribution to the outcome of HBV infection. Future research is necessary to uncover the mechanisms underlying these associations.

## Intrauterine Transmission

Mother-to-child transmission is the major route of HBV infection in developing countries (Stevens et al., 1975; Shao et al., 2007; Li et al., 2015). Intrauterine transmission is the main explanation for mother-to-child transmission, since the implementation of effective measures against HBV infection, such as antiviral treatment, vaccination, and the application of hepatitis B immunoglobulins (Gu et al., 2004; Brown et al., 2016; Ren et al., 2016). Therefore, blocking intrauterine HBV transmission is an important part of the eradication of HBV infection in the general population (Peng et al., 2019). Unfortunately, the incidence of intrauterine HBV infection remains high (Han et al., 2011). The exact mechanism underlying intrauterine infection with HBV has not been fully elucidated (Xu et al., 2008). Previous studies have shown that genetic factors could play an important role in determining the outcome of HBV intrauterine infection (Yu et al., 2006; Xu et al., 2008; Gao et al., 2015b; Liu et al., 2018d). Based on twin and family study, host genetic factors are critical for the development of chronic HBV infection (Lin et al., 1989). Up to date, SNPs in the host genes IFN-γ, TNF-α, CXCL13, PDCD1, TLR3, and TLR9 were found to be relevant for intrauterine transmission (Yu et al., 2006; Gao et al., 2015b; Wan et al., 2016; Liu et al., 2018c; Gao et al., 2015b) (**Table S3**).

A number of host genes may play important roles in HBV intrauterine infection (**Table 3**, **Tables S1**, **S3**). Yu et al. (Yu et al., 2006) showed that a SNP (+874) in IFN-γ is associated with HBV intrauterine infection (both *P* < 0.05). Wan et al. (Wan et al., 2016) investigated associations between candidate genes (SLC10A1, HLA-DP, HLA-C, CXCR5, CXCL13, TLR-3, TLR-4, TLR-9, and UBE2L3) and HBV intrauterine infection in a sample of 44 neonates with HBV intrauterine infection and 662 neonatal controls. They demonstrated that only the rs355687 CT genotype of CXCL13 was associated with susceptibility to HBV intrauterine infection (OR [95% CI] = 0.25 [0.08–0.82], *P* = 0.022).

**Table 3:** Host genetic factors associated with intrauterine hepatitis B infection or response to interferon-alpha (IFN-α) or nucleos(t)ide analog (NUC).

Category	Gene ontology	Gene	Genetic determinants (SNP/Hap/CNVs)	Main reference
Host genetic factors associated with susceptibility to intrauterine hepatitis B infection				
II	Cytokines	IFN-γ	+874	Yu et al., 2006
	Chemokines	CXCL13	rs355687	Wan et al., 2016
Host genetic factors associated response to IFN-α treatment				
I	HLA	HLA-DP	rs3077	Tseng et al., 2011; Tangkijvanich et al., 2016
	Chemokines	IL-28B	rs8099917, rs12979860, rs12980275	Sonneveld et al., 2012; Guo et al., 2013; Boglione et al., 2014; Wu et al., 2015a; Cusato et al., 2017
	Others	CYP27B1	rs4646536	Boglione et al., 2015; Cusato et al., 2017; Limothai et al., 2017
II	HLA	HLA-DP	rs9277535	Brouwer et al., 2014; Cheng et al., 2014
		HLA-DQA1-DQB1-DRB1	*0302-*0303-*09	Zhu et al., 2011
		HLA-DQB1	*0303	Zhu et al., 2011
		HLA-DRB1	*08	Zhu et al., 2011
		HLA-A, B, C	*1101-*4601-*0102	Zhu et al., 2011
	Cytokines	IL-28B	A-C^1^	Wu et al., 2015a
	Others	CYP24A1	rs2248359	Cusato et al., 2017
		CYP27B1	rs10877012	Cusato et al., 2017
		G3BP2	rs3821977	Brouwer et al., 2019
		OAS	G-T-G-A^2^, C-C-T-A^3^, C-C-C-A^4^, A-C-T-A^5^	Wu et al., 2009; Ren et al., 2011
		OAS3	rs2072136	Ren et al., 2011
		PRELID2	rs371991	Brouwer et al., 2019
		STAT4	rs7574865	Jiang et al., 2016a
		TRAPPC9	rs78900671	Brouwer et al., 2019
		VDBP	rs7041	Cusato et al., 2017
		VDR	rs1544410, rs731236, rs11568820, rs10735810	Cusato et al., 2017
Host genetic factors associated response to NUC treatment				
II	HLA	HLA-DP	rs3077	Hosaka et al., 2015; Su et al., 2018
		HLADQ	rs9275572, rs9276370	Chang et al., 2014; Zhang et al., 2014b

**Haplotypes**

^1^A-C, rs12980275-rs12979860; ^2^G-T-G-A, rs3177979-rs1293747-rs4767043-rs10849829; ^3^C-C-T-A, rs2285934-rs2072138-rs2072136-rs10849829; ^4^C-C-C-A, rs2285934-rs2072138-rs2072136-rs10849829; ^5^A-C-T-A, rs2285934-rs2072138-rs2072136-rs10849829.

A case–control study of the Chinese population including 69 HBsAg-positive mother–newborn pairs with intrauterine infection and 138 mother–newborn pairs without intrauterine infection assessed the involvement of the LTβR/APOBEC3B signaling pathway and PD-1/PD-L1 signaling pathway genes, including LTA, LTBR, TNFSF14, PDCD1, APOBEC3B, CD274, CD40, and CD40LG, in HBV intrauterine transmission. They found that the maternal rs2227981 TT genotype of the PDCD1 gene is associated with a decreased risk of intrauterine HBV infection (OR 0.11, 95% CI = 0.01–0.95, *P* = 0.045). There was no significant correlation between the remaining genes and the risk of intrauterine HBV infection (Liu et al., 2018c). Only a few studies have evaluated genetic susceptibility to intrauterine transmission. It is often difficult to distinguish whether a child acquired an HBV infection by the intrauterine or intra-natal route. HBV infection by intrauterine transmission is mainly identified by a blood test at birth. However, this approach may result in false positives due to maternal blood contamination during birth, especially when sensitive PCR methods for HBV DNA detection are used. Moreover, the sample sizes of existing studies are not large enough; thus, the reliability of studies could be questioned. Further research is needed to improve the diagnostic accuracy of intrauterine HBV infection and explore the relationship between HBV intrauterine infection and susceptibility genes as well as other risk factors associated with HBV intrauterine infection.

There is no effective intervention available to prevent intrauterine HBV infection. Neither antiviral treatment during the late phase of pregnancy nor perinatal vaccination/hyperimmunoglobulin application changed the rate of intrauterine HBV infection (Pan et al., 2012a; Brown et al., 2016). Therefore, the identification of relevant genetic determinants could be very helpful.

## Occult HBV Infection

OBI is defined by serum HBV DNA positivity but negative serum HBsAg detection (Torbenson and Thomas, 2002; Raimondo et al., 2008; Raimondo et al., 2019; Seed et al., 2019). If blood from patients with OBI is transfused to other persons, post-transfusion hepatitis may occur (Seo et al., 2011). OBI may also be associated with hepatitis B vaccination failure, vertical HBV transmission, organ transplant failure, cirrhosis, and liver cancer (Raimondo et al., 2013).

The mechanisms underlying OBI have yet to be elucidated and may be related to HBV mutations in the pre-S/S genomic region or antigen–antibody cycle immune complex formation (Zhang et al., 2011b; Samal et al., 2012). Some investigators have suggested that host genetic factors, especially immune-related factors including HLAs, IL10, CXCL12, and VDR, may play a role in the occurrence of OBI (Arababadi et al., 2010; Hassanshahi et al., 2010; Mardian et al., 2017; Wang et al., 2017b) (**Tables S1**, **S4**). Wang et al. (Wang et al., 2017b) performed a case–control study with 107 patients with OBI and 280 healthy individuals to assess associations of SNPs in HLA loci, including HLA-A, -B, -C, -DRB1, and -DQB1, in Chinese patients. They found that HLA-B*44:03 (OR = 2.146, 95% CI = 1.070–4.306, *P* = 0.028), C*07:01 (OR = 4.693, 95% CI = 1.822–12.086, *P* = 0.000), DQB1*02:02 (OR = 1.919, 95% CI = 1.188–3.101, *P* = 0.007), and DRB1*07:01 (OR = 2.012, 95% CI = 1.303–3.107, *P* = 0.001) were strongly associated with susceptibility to OBI, while DRB1*08:03 (OR = 0.395, 95% CI = 0.152–1.027, *P* = 0.049), DRB1*15:01 (OR = 0.495, 95% CI = 0.261–0.940, *P* = 0.029), and DQB1*06:02 (OR = 0.500, 95% CI = 0.249–1.005, *P* = 0.048) alleles were more prevalent in the healthy control group. Mardian et al. (Mardian et al., 2017) found a significant association between the minor allele “T” of HLA-DP (rs3077) and the TGA haplotype (rs3077–rs3135021–rs9277535) and OBI in Indonesian blood donors. Therefore, HLA polymorphisms are an important factor in OBI infection and additional studies are needed to validate these findings. Iranian researchers have shown that the genotypes and alleles of IL-10 (-592) (Ahmadabadi et al., 2012), the +801 region of CXCL12 (Hassanshahi et al., 2010), and the T/T allele of exon 9 of VDR (Arababadi et al., 2010) may be associated with OBI.

Owing to the low frequency of OBI, the accumulation of cases is difficult and time-consuming. Thus, the number of cases included in studies is always small (generally less than 100 cases). In addition, the occurrence of OBI is also related to HBV mutations (Samal et al., 2012). OBI diagnostic criteria are not uniform among studies. Both viral and host factors need to be considered in future analyses. Patients with OBI are positive for HBV DNA but negative for HBsAg. Thus, there could be specific mechanisms for the control of HBsAg production without affecting HBV DNA synthesis. Recently, Lin et al. found that autophagy and related genes (e.g., Rab7) may determine HBsAg production (Lin et al., 2017; Lin et al., 2019), but further studies are needed to evaluate this.

## Antiviral Efficacy of Interferon-α or Nucleos(t)ide Analogues

IFN-α and NUCs have been approved and are widely used for the treatment of chronic hepatitis B and slow down liver disease progression, cirrhosis, and HCC (Lee and Keeffe, 2011; European Association For The Study Of The Liver, 2017). However, NUCs and IFN-α alone or combined treatment are rare to achieve functional cure (Reijnders et al., 2010; Zoulim and Locarnini, 2012; Terrault et al., 2016; Revill et al., 2019). Extensive studies have identified host genetic genes, alanine aminotransferase (ALT) levels, HBV genotype, and HBV DNA, anti-HBc, and HBsAg levels as important predictors of treatment efficacy (Brouwer et al., 2014; Fan et al., 2016b; Zhu et al., 2016a). Accumulating evidence suggests that host genetics play an important role in the patient response to IFN-α or NUCs (**Table 3**; **Tables S1**, **S5**, **S6**).

IFN-α is a cytokine with a broad-spectrum effect against viruses and tumors. It is able to inhibit cell growth and exerts immunomodulatory effects. IFN-α exerts antiviral activity mainly *via* the Janus-activated kinase-signal transducer and activator of transcription (JAK-STAT) signaling pathway and interferon-stimulating genes (ISGs) (Levy and Darnell, 2002). IFN-α binds to type I IFN receptor (IFN-αR1) on the surface of target cells and induces the synthesis of antiviral proteins, such as MxA, 2’,5’-oligoadenylate synthetase (OAS), protein kinase (PKR), and adenosine deaminases acting on RNA (ADAR) (Samuel, 2001). In addition, IFN-α upregulates HLAs and thereby modulates adaptive immunity. Thus, host genetic polymorphisms, particularly those associated with IFN signaling pathways, may potentially alter the responsiveness to IFN-α therapy. Many studies have evaluated the relationship between the outcome of IFN-α treatment for CHB and host genetics and identified the potential relevance of HLAs, STAT4, vitamin D–related genes, and some ISGs (Tseng et al., 2011; Brouwer et al., 2014; Wu et al., 2014; Jiang et al., 2016a; Thanapirom et al., 2017; Brouwer et al., 2019) (**Table S5**).

The first GWAS published to date did not find any candidate gene associated with the efficacy of IFN-α treatment. This may be explained by an insufficient sample size, with only 43 patients (Chang et al., 2014). Recently, Brouwer et al. (Brouwer et al., 2019) published a GWAS on the efficacy prediction of PEG-IFN in the treatment of CHB, including 1,058 patients from 21 centers from Europe, Asia, and North America. A SNP rs78900671 GC allele (TRAPPC9, COL22A1) had been identified to have the strongest association with response to PEG-IFN treatment (*P* = 6.43×10^-7^). Another SNP PRELID2 rs371991 (*P* = 3.44×10^-6^) was associated with the primary response to PEG-IFN treatment in HBeAg-positive patients while G3BP2 rs3821977 (*P* = 2.46×10^-6^) was associated with response in HBeAg-negative patients. Yet the correlation between these SNP loci and the efficacy of interferon treatment needs confirmation by future studies.

HLA molecules play an important role in the recognition of viral proteins and regulation of adaptive immunity. Adaptive immunity is upregulated by IFN-α treatment and essential for sustained responses and HBV control (Bengsch and Thimme, 2018; Ma et al., 2018b). Thus, HLAs may be an important genetic marker for the prediction of IFN-α efficacy. Many studies have analyzed the relationship between HLA genes and the IFN-α response (Tseng et al., 2011; Zhu et al., 2011; Brouwer et al., 2014; Tangkijvanich et al., 2016). A study of a Taiwan population by Tseng et al. (Tseng et al., 2011) including 115 HBeAg-positive patients with CHB who received peginterferon (PEG-IFN) therapy showed that the HLA-DPA1 rs3077 GG genotype (OR [95% CI] = 3.49 [1.12–10.84], *P* = 0.031) is associated with HBeAg seroconversion at 6 months of therapy. Brouwer et al. (Brouwer et al., 2014) evaluated 262 Caucasian patients with chronic HBV infection who were treated with PEG-IFN for 1 year and found that the HLA-DPB1 (rs9277535) G-allele and haplotype block GG combining HLA-DPB1 (rs9277535) and HLA-DPA1 (rs3077) are strongly associated with virological (HBV DNA <2,000 IU/ml at 6 months post-treatment) and serological responses (HBeAg seroconversion combined with HBV DNA <2,000 IU/ml at 6 months post-treatment) to PEG-IFN therapy. Zhu et al. (Zhu et al., 2011) reported that the HLA-DQB1*0303 or DRB1*08 alleles and the *1101-*4601-*0102 (HLA-A, B, C) or *0302-*0303-*09 (HLA-DQA1, DQB1, DRB1) haplotypes are associated with the efficacy of IFN therapy in the Chinese population. However, some studies have failed to observe a relationship between HLA variants and the response to IFN-α- or PEG IFN-based treatment (Limothai et al., 2016).

IFN-λ3 is encoded by the IL-28B gene, which regulates the immune system by activating the JAK-STAT signaling pathway, thereby exerting an antiviral effect (Liu et al., 2012a). Recent GWAS has revealed that IL28B is associated with the efficacy of anti-HCV treatment (Ge et al., 2009). In a large international collaborative study of 205 HBeAg-positive patients with CHB who received PEG-IFN-α-2a or PEG-IFN-α-2b, IL28B rs12979860 and rs12980275 were independent predictors of HBeAg seroconversion in response to PEG-IFN (Sonneveld et al., 2012). Boglione et al. (Boglione et al., 2014) examined the effect of the IL28B gene polymorphisms rs12979860, rs8099917, and rs12980275 on the response to PEG-IFN in 190 chronically HBV-infected, HBeAg-negative patients and showed that rs12979860 CC, rs8099917 TT, and rs12980275 AA are significantly associated with HBV-DNA < 2,000 IU/ml post-treatment, while rs12979860 CC and rs12980275 AA are significantly associated with qHBsAg > 1 log during therapy. Many other research teams worldwide have indicated that IL-28B is involved in the efficacy of IFN-α therapy (Lampertico et al., 2013; Wu et al., 2015a; Cusato et al., 2017). However, variable and inconclusive results have been obtained (Cheng et al., 2014; Tangkijvanich et al., 2016). A study in Taiwan showed that IL28B rs12979860 is not associated with the virological response to PEG-IFN alpha-2b with or without entecavir in patients with HBeAg-negative CHB (Tangkijvanich et al., 2016). A Chinese study also suggested that IL28B (rs12979860 and rs8099917) has no significant association with the response to IFN-α or PEG IFN (Cheng et al., 2014). To determine whether IL28B polymorphisms play a role in the response to interferons, further large-scale cohort studies of homogenous patient populations are necessary.

Vitamin D has important immunomodulatory effects on innate and adaptive immune responses (Hewison, 2011). Vitamin D enhances the IFN-α-mediated activation of the JAK-STAT pathway, increases the expression levels of antiviral proteins, and improves the overall antiviral ability (Lange et al., 2014). Vitamin D–related genes, including VDR (Cusato et al., 2017), CYP27B1 (Cusato et al., 2017), CYP24A1 (Cusato et al., 2017), CYP2R1 (Thanapirom et al., 2017), DHCR7 (Thanapirom et al., 2017), and DBP/GC (Cusato et al., 2017; Thanapirom et al., 2017), have been evaluated for potential associations with the outcome of IFN-α therapy in patients with CHB. Several studies in China, Thailand, and Italy have shown that polymorphisms in the rs4646536 and rs10877012 loci of CYP27B1 may be related to the efficacy of IFN-α in patients with CHB; OR values reported in studies of Italy (Cusato et al., 2017), Thailand (Limothai et al., 2017), and Italy (Boglione et al., 2015) were 2.87, 3.13, and 3.13 for the rs4646536 locus (all P < 0.01); and the OR values for studies of Italy (Cusato et al., 2017) was 3.13 for the rs10877012 locus (both P < 0.001). Therefore, the TT (rs4646536) and GG (rs10877012) genotypes may be predictors of sustained HBeAg seroconversion in HBeAg-positive patients treated with IFN-α. Cusato et al. (Cusato et al., 2017) evaluated SNPs in vitamin D pathway genes in HBeAg-negative patients with CHB treated with PEG-IFN-α, suggesting that VDR (rs7975232 [ApaI], rs11568820 [Cdx2], rs10735810 [FokI]), CYP27B1 (rs4646536 [+2838], rs10877012 [−1260]), VDBP (rs7041), and CYP24A1 (rs2248359) are correlated with the clinical outcomes of PEG-IFN-α treatment. Thanapirom et al. (Thanapirom et al., 2017) showed that the CYP2R1 (rs12794714) TT genotype is a predictor of sustained HBeAg seroconversion in HBeAg-positive patients treated with Peg-IFN-α (OR [95% CI] = 4.53 [1.51–13.61], *P* = 0.01), whereas SNPs in DHCR7 (rs12785878), CYP27B1 (rs10877012), CYP2R1 (rs2060793), GC (rs4588, rs7041, rs222020, and rs2282679), and VDR (FokI, BsmI, Tru9I, ApaI, TaqI) are not related to HBeAg seroconversion. Several studies have investigated the role of other SNPs in vitamin D pathway genes in predicting the outcome of IFN-α therapy, but their predictive value remains controversial (Thanapirom et al., 2017). Differences among studies could be explained by several factors, such as differences in study design, sample size, ethnicity, and genotyping methods.

SNPs in JAK-STAT pathway genes and ISGs may contribute to the antiviral effects of IFN-based therapy (Kong et al., 2007; Ren et al., 2011; Wu et al., 2014; Jiang et al., 2016a). OAS3, MxA-88, and STAT4 genes in the IFN signaling pathway may have predictive value for the efficacy of interferons, but further studies are needed to verify this.

NUCs (e.g., lamivudine [LAM], adefovir [ADV], telbivudine [LdT], entecavir [ETV], and tenofovir [TDF]) inhibit HBV replication mainly by inhibiting HBV DNA polymerase. They bind competitively to HBV DNA polymerase and are incorporated into the DNA chain, resulting in the termination of DNA synthesis and inhibition of HBV replication (Lok et al., 2007; European Association For The Study Of The Liver, 2017). As the direct effect of NUCs on HBV does not require host functions and may not be directly related to host genetics, few studies have evaluated the correlation between host gene SNPs and drug efficacy, and these have generally yielded negative results (**Table 3**; **Tables S1**, **S6**).

There were significant differences in the patterns of HBsAg decline, and seroclearance during LAM therapy in Japanese patients with HBeAg-positive CHB was associated with HLA-DPA1 (rs3077), HLA-DPB1 (rs9277535), and A alleles at rs3077 and rs9277535 (Hosaka et al., 2015). A GWAS (Chang et al., 2014) with small sample sizes (LAM group, *n* = 119; ETV group, *n* = 64) of male Taiwanese individuals revealed that the TT genotype of rs9276370 (HLA-DQA2) is highly associated with a non-sustained response in the LAM group (OR [95% CI] = 5.41 [1.73–16.95], *P* = 0.0037), but not in the ETV group (OR [95% CI] = 0.68 [0.16–2.86], *P* = 0.5954). Zhang et al. (Zhang et al., 2014b) reported that the HLA-DQ (rs9275572) A allele is associated with viral and biochemical responses to LAM treatment in Chinese patients.

Using a small sample size (*n* = 76), our team evaluated the virological responses associated with the ESR1 PvuII T/C genotype in nucleoside-naive patients with chronic hepatitis B treated with ETV after 48 and 96 weeks of treatment; we did not detect associations between XbaI polymorphisms and virological response (Zhang et al., 2013c). Another study also found that CTLA4 (rs231775) and HLA-DPA1 (rs3077) are associated with predictors of relapse and outcomes after discontinuing TDF and ETV therapy in Taiwan patients with CHB (Su et al., 2018). However, the sample sizes of these studies are insufficient.

The main issues with studies of the efficacy of IFN/NUCs are as follows. 1) ALT, HBsAg, HBeAg, anti-HBc, HBV DNA, and HBV genotype may affect the efficacy of IFN/NUCs, and it is difficult to control for these confounding factors. 2) Some studies do not distinguish between HBeAg-positive or -negative patients, resulting in the inconsistent use of drugs as well as differences in treatment duration. 3) The criteria for determining efficacy and time points for evaluations are often inconsistent. 4) Many studies include small sample sizes and a lack of secondary verification. 5) Different and even contradictory results have been reported. Despite studies showing that several SNP sites in IL28B are closely related to the efficacy of IFN-α for the treatment of HCV infection, it is difficult to confirm that any host genetic determinant is significantly related to the efficacy of IFN-α or NUCs in chronic HBV infection without resolving these issues. It is necessary to clarify whether host genetic factors determine the efficacy of antiviral therapies. Currently, available antiviral treatments based on IFN-α/NUCs are not effective to clear HBV covalently closed circular DNA (cccDNA). Information about the involvement of host factors in antiviral activity may be helpful to optimize treatment approaches.

Attempts to improve the response by administering two different NUCs or a combination of NUCs and IFN-α failed to increase the rate of “functional cure.” New drug candidates targeting different steps of HBV life cycle including entry, cccDNA formation, viral transcription, capsid assembly, and secretion of viral envelope proteins were developed and partly tested in clinical trials (Qazi et al., 2018; Tu and Urban, 2018; Yang and Lu, 2018; Yang et al., 2019). Immunotherapeutic approaches to enhance antiviral immunity using IL-15, TLR agonists, retinoic acid inducible gene I (RIG-I) agonist, stimulator of IFN genes (STING) agonist or based on engineered T cells are also under investigation (Ma et al., 2015; Zhang and Lu, 2015; Guo et al., 2017; Koh et al., 2018). A new drug targeting cIAPs has being now tested in a clinical trial phase I (Liu et al., 2018a). Checkpoint inhibitors like anti-PD1 are able to stimulate host adaptive immunity and considered as potential drug candidates to treat chronic HBV infection (Liu et al., 2014a; Liu et al., 2018b). Such innovative drugs, Along with current available therapies, will offer the prospect of a markedly improved response to treatments and an increased rate of functional cure (Ko et al., 2015; Blank et al., 2016; Di Scala et al., 2016; Zhu et al., 2019). Those new drug candidates, especially for immunotherapies, may exert the antiviral actions in dependence on host genetic background. Some genetic determinants found in the previous studies like SNPs in HLA loci may reappear again in such studies. Their efficacy in anti-HBV treatment related to human genetics remains to be a topic for future studies.

## Response to Hepatitis B Vaccination

Hepatitis B vaccines are widely used for the prevention of new HBV infections and are the most effective way to prevent HBV transmission (Kew, 1995; Ni et al., 2012; Bruce et al., 2016). The overall success rate of vaccination is about 90%. Several factors, such as age, gender, body mass index (BMI), immunosuppression, vaccine escape mutations, and premature birth, are associated with a failure to respond to hepatitis B vaccination (Zuckerman, 2006; Kang et al., 2014; Yang et al., 2016b). A twin study indicated that the immune response to hepatitis B vaccination has a high heritability (77%) (Newport et al., 2004). Yan et al. investigate the overall contribution of genetic and environmental effects on poor response to HBV vaccination in Chinese infants. They found that the HBV vaccine response in infants is dominantly determined by genetic effect by 91%, compared with perinatal environmental factors (Yan et al., 2013). Genetic factors may have important roles in the immune response to hepatitis B vaccination, and many studies have focused on the identification of these genes including HLAs and IL4 (Png et al., 2011; Cui et al., 2013; Li et al., 2013b; Nishida et al., 2018) (**Table 4**; **Tables S1**, **S7**).

**Table 4:** Host genetic factors associated with response to hepatitis B vaccine.

Category	Gene ontology	Gene	Genetic determinants (SNP/Hap/CNVs)	Main reference
I	HLA	HLA-DP	rs9277535	Png et al., 2011; Wu et al., 2015b
		HLA-DQB1	*02, *05, *0501, *06, *0602	Li et al., 2013b; Nishida et al., 2018
		HLA-DRB1	*01, *03, *0301, *04, *07, *1301, *1302,*15	Li et al., 2013b; Yoon et al., 2014; Nishida et al., 2018
	Cytokines	IL-4	rs2070874, rs2227284, rs2243250	Wang et al., 2012; Cui et al., 2013
II	HLA	HLA-B	*62	Yoon et al., 2014; Roh et al., 2016
		HLA-DP	rs2116260, rs3128961, rs35953215, rs3830066, rs4282438, rs5025825, rs6457709, rs7770370, rs9277542, *04:02, *05:01, *09:01, *02:02, *03:01:01, *04:01:01, A-G-A-G-G^1^, A-G^2^, A-G-A-G-G-A-G^3^	Wu et al., 2015b; Sakai et al., 2017; Nishida et al., 2018
		HLA-DQB1	*0401	Xu et al., 2017a; Nishida et al., 2018
		HLA-DR	rs3135363	Png et al., 2011
		HLA-DRA	rs7192, rs2395177, rs5000563	Davila et al., 2010
		HLA-DRB1	*01:01, *04:05, *08, *08:03, *15:01, rs477515, rs13204672, rs28366298	Sakai et al., 2017; Nishida et al., 2018
		HLA-DRB1-DQB1	*01:01-*05:01,*04:05-*04:01,*08:03-*06:01,*14:06-*03:01, *15:01-*06:02	Nishida et al., 2018
		HLA-III	rs9267665	Png et al., 2011
	Others	BTNL2	rs2076530, rs3763311, rs3763313, rs3763316, rs3806156, rs3817963, rs4248166, rs9268494, rs9268501, A-A^4^, C-G^5^, G-T-A-T-C-A-G^6^, G-T-A-T-C^7^, G-C-A-T-C^8^	Davila et al., 2010; Pan et al., 2014; Yang et al., 2014; Nishida et al., 2018
		FOXP1	rs6789153	Davila et al., 2010
		LILRB4	rs1654668	Davila et al., 2010

**Haplotypes**

^1^A-G-A-G-G, rs5025825-rs6457709-rs35953215-rs3830066-rs7770370; ^2^A-G, rs3128961-rs9277535; ^3^A-G-A-G-G-A-G, rs5025825-rs6457709-rs35953215-rs3830066-rs7770370-rs3128961-rs9277535; ^4^A-A, rs3806156-rs2076530; ^5^C-G, rs3806156-rs2076530; ^6^G-T-A-T-C-A-G, rs9268501-rs3763316- rs3763313-rs3763311- rs9268494- rs3806156-rs2076530; ^7^G-T-A-T-C, rs9268501-rs3763316- rs3763313-rs3763311-rs9268494; ^8^G-C-A-T-C, rs9268501-rs3763316- rs3763313-rs3763311-rs9268494.

A recent GWAS has shown that genetic variants in HLA loci are strongly associated with chronic HBV infection. Difference in host immune responses to HBV antigens are largely explained by HLA alleles (Ovsyannikova et al., 2004). Associations of SNPs in HLA regions, including -A, -B, -DR, and -DQ loci, with the response to hepatitis B vaccines have been identified (Davila et al., 2010; Png et al., 2011; Yoon et al., 2014; Roh et al., 2016). A GWAS of an Indonesian population including 3,614 hepatitis B vaccine recipients suggested that HLA loci, including HLA-DR (rs3135363) (*P* = 6.53 × 10^−22^; OR = 1.53, 95% CI = 1.35–1.74), HLA-DP (rs9277535) (*P* = 2.91 ×10^−12^; OR = 0.72, 95% CI = 0.63–0.81), and HLA class III (rs9267665) (*P* = 1.24 × 10^−17^; OR = 2.05, CI = 1.64–2.57), are strongly associated with the response to hepatitis B vaccination (Png et al., 2011). Other GWAS have found that HLA-DRB1 (rs477515, rs28366298, and rs13204672), HLA-DP (rs7770370), and multiple HLA-DRB1-DQB1 haplotypes are associated with the response to hepatitis B vaccines (Davila et al., 2010; Wu et al., 2015b). Although similar results have been obtained in several additional studies (Yoon et al., 2014; Roh et al., 2016; Sakai et al., 2017), other studies do not support these associations, likely owing to the sample sizes, statistical methods, or the use of different vaccines (Okada et al., 2017; Xu et al., 2017a). A meta-analysis (Li et al., 2013b) has shown that HLA class II genotypes (HLA-DRB1*01, DRB1*1301, DRB1*15, HLA-DQB1*05, DQB1*0501, DQB1*06, DQB1*0602) are associated with the antibody response to the hepatitis B vaccine, while the opposite results were found for DRB1*03 (DRB1*0301), DRB1*04, DRB1*07, DRB1*1302, and DQB1*02. Taken together, HLA is an important marker for the response to the hepatitis B vaccine; multicenter studies are needed for verification and to determine the mechanism underlying this association.

Cytokines play key roles in regulating immune responses and may modulate responses to HBV vaccines (Harber et al., 2000; Howe et al., 2005). Among these cytokine genes, IL-4 induces the differentiation of naive helper T cells to Th2 cells and is a key regulator of humoral immune responses. Genetic polymorphisms in IL-4 have been reported to be associated with the response to the hepatitis B vaccine in many studies (Chen et al., 2011; Wang et al., 2012), such as IL-4 (rs2070874, rs2243250, rs2227282, rs2243248, rs2227284) (Chen et al., 2011; Wang et al., 2012), IL-4RA (rs1805015) (Chen et al., 2011), IL-12B (rs17860508) (Pan et al., 2012b), and IL-13 (rs1295686) (Chen et al., 2011) that are associated with responses to the hepatitis B vaccine. In a meta-analysis of eight published studies, Cui et al. (Cui et al., 2013) evaluated associations of IL4 genetic polymorphisms, including rs2243250 (C > T), rs2070874 (C > T), rs2227284 (A > C), rs2227282 (C > G), and rs2243248 (G > T), with the response to the hepatitis B vaccine. The analysis suggested that the T allele of rs2243250, the T allele of rs2070874, and the C allele of rs2227284 in IL4 may be useful biomarkers for predicting favorable responses to the hepatitis B vaccine, especially in the Asian population. Relatively few studies have examined other cytokine genes.

Other studies have suggested that polymorphisms in other host genes (BTNL2, FOXP1, LILRB4, TLR2, VDR, etc.) may be related to responses to hepatitis B vaccination (Davila et al., 2010; Chen et al., 2011; Grzegorzewska et al., 2014), but further validation of these results is required.

The overall success rate of vaccination is about 90% (Zuckerman, 2006), and the factors that affect the success of vaccination mainly include 1) the type of vaccine; 2) the dose, mode, and frequency of inoculation; 3) the vaccination schedule; 4) ethnic differences; 5) the age at vaccination; 6) the presence of severe heart, liver, and kidney disease; 7) OBI status; and 8) assessment criteria for successful vaccination. When analyzing the effect of SNPs on the success of vaccination, it is important to match patients with respect to the main factors or perform appropriate statistical analyses to account for these factors. However, at present, correlation analyses are relatively simple. In addition, most subjects are under 20 years of age, and age is related to the rate of antibody production in response to the hepatitis B vaccine. These factors can lead to differences and contradictory results among studies. Further in-depth analyses of host genes may be helpful for understanding the mechanisms underlying the response to hepatitis B vaccination and for exploring new approaches for the development of more effective hepatitis B vaccines. Furthermore, studies should evaluate whether so-called non-responders, who do not produce anti-HBs antibodies after vaccination, may develop chronic HBV infection. It would be useful to determine the genetic factors leading to non-responsiveness to HBV vaccines.

## Conclusion

**Figure 3:**
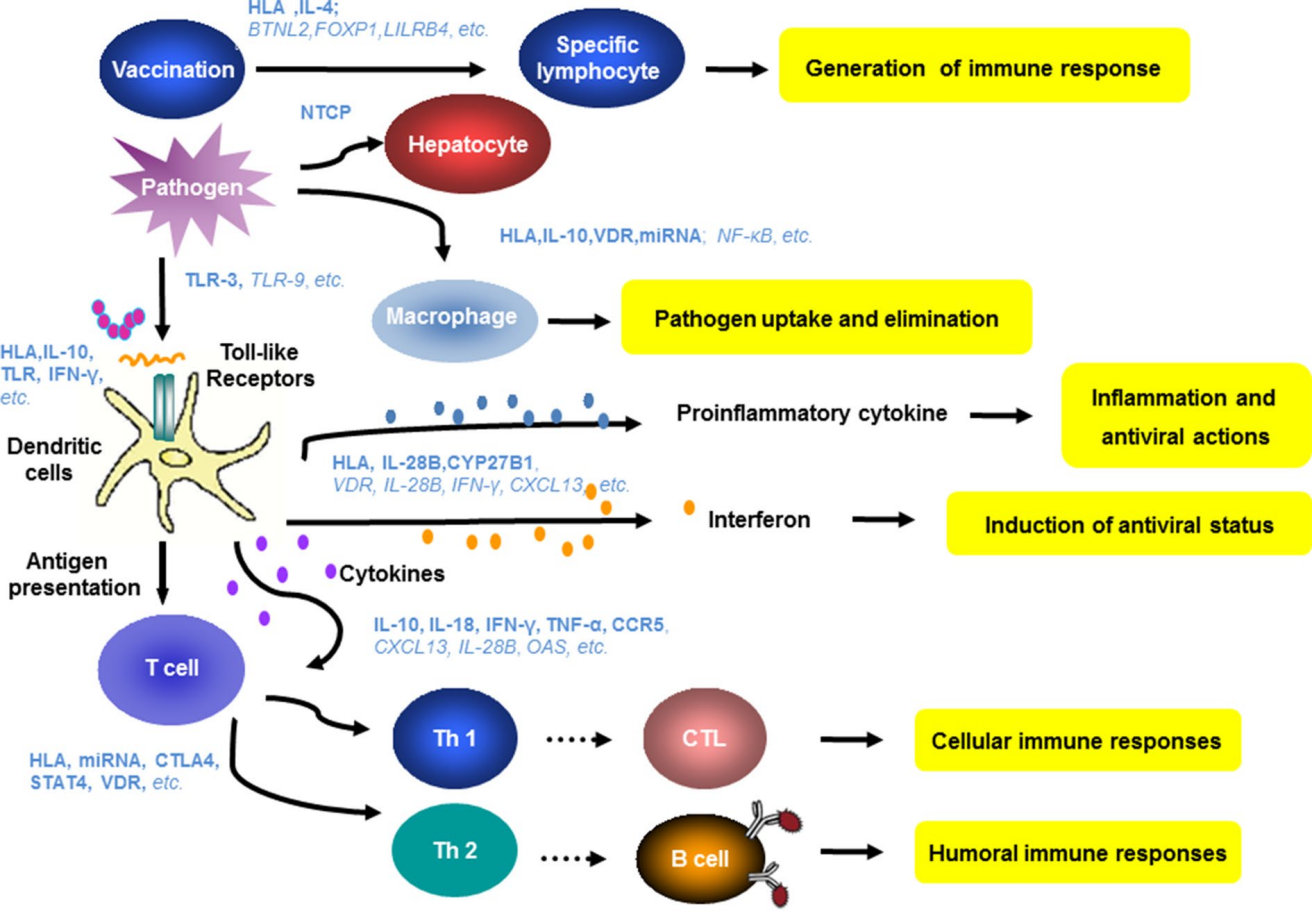
Host genes and immune mechanisms involved in HBV infection. Host innate and adaptive immunity represent the major determinants of the outcome of HBV infection. The genetic diversity of many genes related to innate and adaptive immunity have been found to be relevant for hepatitis B vaccination, HBV infection, and antiviral therapy. Blue letters show the relevant genes identified in the previous studies. The bold and italic letters indicate a high degree or potential correlation of the indicated host factors to clinical features of HBV infection.

A large number of studies have reported host genetic factors that are associated with chronic HBV infection, clinical type, therapeutic responses, or responses to hepatitis B vaccines (**Figure 2**). Host innate and adaptive immunity represent the major determinants of the outcome of HBV infection. The genetic diversity of many genes related to innate and adaptive immunity have been found to be relevant for hepatitis B vaccination, HBV infection, and antiviral therapy (**Figure 3**).

HBV vaccines induce specific B cell responses, leading to the production of protective antibodies. The genetic polymorphism in many genes like HLAs and IL-4 has an impact on antigen presentation or cytokine production and thereby on antibody production, resulting in different degrees of responsiveness to vaccination (Png et al., 2011; Wang et al., 2012; Cui et al., 2013; Li et al., 2013b; Akcay et al., 2018; Nishida et al., 2018). The polymorphism in NTCP may determine the process of HBV entry (Yan et al., 2012a; Hu et al., 2016; Yang et al., 2016a; Nfor et al., 2018; Wu et al., 2018a). Many host factors may influence the innate and adaptive immunity including activation and phagocytosis of macrophages, antigen presentation by APCs, and priming and functionality of HBV-specific T cells, influencing HBV clearance and persistence (Bertoletti and Ferrari, 2016; Akcay et al., 2018; Lang et al., 2019; Rehermann and Thimme, 2019). It is well recognized that the recognition and presentation of HBV antigens by DCs to specific T cells initiate specific T cell antibody responses (Schurich et al., 2011; Das et al., 2012; Guidotti et al., 2015; Gehring and Protzer, 2019; Rehermann and Thimme, 2019; Zhu et al., 2019). IFNs and proinflammatory cytokines also contribute to HBV pathogenesis and control (Maini and Gehring, 2016; Hong and Bertoletti, 2017; Lang et al., 2019; Zhu et al., 2019). The genetic polymorphisms in relevant human genes could directly or indirectly determine these immune functions (**Figure 3**). Owing to the complexity of the genetic basis for infection characteristics, it is unlikely that clinical parameters can be attributed to variation in a single genetic factor. Furthermore, contradictory results are not unexpected. As age and mother-to-child transmission of HBV infection have great impacts (Li et al., 2015), host genetic factors are usually not the only determinants for chronic HBV infection, HBV intrauterine infection, its clinical outcome, or antiviral therapies.

Owing to the complexity of the pathogenesis of HBV, future studies should apply strict criteria for patient selection and for the selection of matched controls according to various factors, like age, route of transmission, time of infection, race, and host immune status, to identify relevant host factors. It is also necessary to standardize the diagnosis of HBV-related diseases. Results should be verified using different ethnic groups and confirmed by future controlled studies with large sample sizes as well as meta-analyses. Importantly, understanding the biological functions of candidate genetic markers is essential to gain mechanistic insight into HBV-related diseases.

At the moment, genetic markers associated with host responses to HBV infection/vaccines and HBV-related diseases could be classified into three categories according to the quality of studies. Some markers, like human HLAs, are clearly important determinants of HBV pathogenesis and the response to HBV vaccines. However, the precise functions of these loci in HBV infection are unclear. For example, the immunological effects of genetic variation in HLAs are not obvious, though these mutations are likely to contribute to HBV-specific immunity. Future research should consider interactions between genetic factors and other factors, including viral genotypes/subtypes, population characteristics, other genes, and environmental factors (**Table 1**; **Table S1**). Additional candidate genes that may play a role in the pathogenesis of HBV require confirmation by large-scale, multi-center validation studies. In these future studies, it will be important to stratify populations, match samples, and choose appropriate control groups to improve the reliability of results (**Table 1**; **Table S1**). Additional candidate genes with unconfirmed relevance owing to small samples sizes and studies with different designs should be evaluated by future controlled studies with suitable study subjects and meta-analyses (**Table 1**; **Table S1**).

Recently, our understanding in HBV infection and pathogenesis is fast growing. The knowledge about the molecular and physiological mechanisms of HBV pathogenesis will help us to improve future study design and develop better criteria for patient recruitment. New diagnostic methods like quantitative HBsAg and anti-HBc assays may provide more information to select suitable patient cohorts. The development of genomic and proteomic analysis tools will also enable us to define novel parameters—for example, at the level of single cells or protein chemistry, thereby opening new ways to define genetic determinants for HBV pathogenesis.

## Author Contributions

ZZ and CW reviewed the literature. ZZ, CW, ZL, and GZ wrote the manuscript. JL and ML participated in the coordination of the study and manuscript modification. ML conceived the project. All authors contributed, read, and approved the manuscript.

## Funding

This work was supported by the Natural Science Foundation of Anhui Province (Grant no. 1608085MH162).

## Conflict of Interest Statement

The authors declare that the research was conducted in the absence of any commercial or financial relationships that could be construed as a potential conflict of interest.
