# Supplementary material for: Host Genetic Determinants of Hepatitis B Virus Infection
Source: Front Genet. 2019 Aug 13;10:696. doi: 10.3389/fgene.2019.00696 (PMC6702792; doi:10.3389/fgene.2019.00696)
Supplement: Supplementary file 1 [file Table_1.doc]

**Supplement Table S1. Classification of host genetic determinants for HBV-related diseases and responses to HBV.**

| Category | CHI |  | IT |  | OBI |  | IFN |  | NUCs |  | Vaccine |
| --- | --- | --- | --- | --- | --- | --- | --- | --- | --- | --- | --- |
| Ⅰ | CTLA-4, HLA-B, HLA-DPA1, HLA-DPB1, HLA-DQ, HLA-DQB1, HLA-DQB2, HLA-DRB1, IFN-γ, IL-18, MiR-106b-25, miR-122, miR-196a-2, miR-let-7c, NTCP, STAT4, TCF19, TLR-3, TNF-α, VDR |  |  |  |  |  | CYP27B1 |  |  |  | HLA-DP, HLA-DQB1, HLA-DRB1, IL-4 |
| Ⅱ | C2, CCR5,CD40, CFB, EHMT2, E-selectin ,ESR1, HL-A, HLA-C, HLA-DOA, HLA-DP, HLA-DPA1/DPB1, HLA-DP/DQ, HLA-J, IFN4/HLA-DQ, IFNLR1, IFN-γR2, IFN- α2, IFN-αR1, IFN-αR2, IFN-γR1, IL-10, IL-10RB, IL-12B, IL-16, IL-17A, IL-17F, IL-21,INST10, KIF1B, MCP1, MIF, miR-101-2, miR-219-1, miR-30a, miR-323b, miR-423, miR-492, MxA, MX1, NF-κB, NLRX1, NOTCH4, OCT4, PAPL, SOCS3, SPP1, TGF-α, TGF-βR3, TLR-9, TMEM2, TMEM2/IFNA2/NLRX1/C2, UBE2L3, VARS2, ZNRD1 |  | CXCL13, IFN-γ,  TNF-α |  |  |  | HLA-DP, IL-28B, OAS, VDR |  | HLA-DP |  | BTNL2, FOXP1, HLA-B, HLA-DR, HLA-DRA, HLA-DRB1/DQB1, HLA-III, LILRB4 |
| Ⅲ | AGO1, AGT, APOBEC3, APOBEC3G, AQP2, AZIN1, BAFF, CCR2, CCRL2, CCRL2/CCR5, CTLA-4, CXCR1, CXCR4, CYP27B1, CDH1, COX2, CXCL10, DEPDC5, DEGS1, DGCR8, FAS, FASL, FOXP1, GEMIN4, GNLY, HLA-F, HLA-G, HLA-E, IL-12A, IFN-α1, IFN-α5, IFNα5/IFNα1, IFNαR2/IL10RB, IL-1R1, IL-1β, IL-1RN, IL-2, IL-4, IL-6, IL-12, IL-13, IL-21/IL-21R, IL-21R, IFNL4, IL-1A, IL-2, IL-15, IL-27, IL-27B, IL-29, INF-αR1, IRAK1, IRF-3, IRF5, ITNF-β, KIR, KIR/HLA-C, MBL, MDR1, miR-146a, miR-149, miR-218, miR-34b/c, miR-499, miR-101-1, miR-26a-1, miR-338, miR-604, miR-608, MYD88, NF-κB1, NF-κB2, PAK4, PTEN, RANTES, RF-1, SFTA2, STAT3, TNF, TP53, TRPM5, TRIM22, TLR-4,TLR-5,THBS2, THBS4,VIM |  | APOBEC3B, CD274, CD40, CD40LG, CXCR5, DC-SIGN, DC-SIGNR, HLA-C, HLA-DP, HLA-DR, HLA-DRB1  , IL-4, IL-10, LTA, LTBR, NTCP, PDCD1, TLR-3, TLR-4, TLR-9, TNFSF14, UBE2L3 |  | CTNNB1, CXCL12, FAS, HLA-B, HLA-C, HLA-DP, HLA-DQB1, HLA-DRB1, IFN-γ, IL-10, IL-12, IL-28B, TP53, VDR |  | ADAR, ADAR1, CYP24A1, CYP2R1, DHCR7, DBP, eIF-2α, eIF-2S1, ESR1, GBP2, GC, HLA-A/B/C, HLA-DQ, HLA-DQA1/DQB1/DRB1, HLA-DQB1, HLA-DRB1, HLA-B, HLA-C, IFNL4, IL-10, IL-1RN, IL-1β, IFNAR1, IL-21, IL-21R, IP-10, JAK, JAK1, KIR, KIR/HLA-C, KIR-HLA, MxA, NTCP, OAS1, OAS2, OAS3, OASL, PKR, STAT1, TAT4, VDBP |  | CD40, CFB, CTLA-4, ESR1, HLA-DRB1, IL-10, KIR, NOTCH4, NTCP, IL-28B |  | BCL6, CCL15, CCR2, CCR5, CD58, FCN2, GC, HLA-A, HLA-DQ, HLA-I, HLA-II, IL-10, IL-12A, IL12A/IL12B, IL-12B, IL-13, IL-17, IL-1β, IL-2, IL-22, IL-4RA, ITGAL, MBL2, RXRA, SC, TGFB3, TLR-2, TNF, TNFRSF1A, TNFSF15, VDR |

**Note:**

CHI, Chronic HBV infection; II,Intrauterine HBV infection; OBI, Occult HBV infection; IFN, Response to interferon therapy; NAs, Response to nucleotide analogues therapy; Vaccine, Response to hepatitis B vaccination

**Gene Abbreviation**

ADAR, Adenosine to inosine acting on RNA enzyme; ADAR1, Adenosine to inosine acting on RNA enzyme 1; AGO1,Argonaute-1; AGT, Angiotensinogen; APOBEC3, Apolipoprotein B mRNA editing enzyme, catalytic polypeptide-like 3; APOBEC3B, Apolipoprotein B messenger RNA-editing, enzyme-catalytic, polypeptide-like 3B; APOBEC3G, Apolipoprotein B mRNA editing enzyme, catalytic polypeptide-like 3G; AQP2, Aquaporin 2; AZIN1, Antizyme inhibitor 1; BAFF, B cell activating factor; BCL6, B-cell lymphoma 6 protein; BTNL2, Butyrophilin Like Protein 2; C2, Complement component 2; CCL15, C-C motif chemokine ligand 15; CCR2, Chemokine receptor 2; CCR5, Chemokine receptor 5; CCRL2, Chemokine receptor-like 2; CD274, Cluster of differentiation 274; CD40, Cluster of differentiation 40; CD40LG, CD40 Ligand; CD58, Cluster of differentiation 58; CDH1, Cadherin 1; CFB, Complement factor B; COX2, Cyclooxygenase 2; CTLA-4, Cytotoxic T lymphocyte antigen 4; CTNNB, Catenin beta-1; CXCL10, Chemokine motif ligand 10; CXCL12, Chemokine motif ligand 12; CXCL13, Chemokine motif ligand 13; CXCR1, Chemokine receptor 1; CXCR4, Chemokine receptor 4; CXCR5, Chemokine receptor 5; CYP24A1, Cytochrome P450 family 24 subfamily A member 1; CYP27B1, Cytochrome P450, family 27, subfamily B, peptide 1; CYP2R1, Cytochrome P450 family 2 subfamily R member 1; DBP, Vitamin D binding protein; DC-SIGN, Dendritic Cell-Specific Intercellular adhesion molecule-3-Grabbing Non-integrin; DC-SIGNR, Dendritic Cell-Specific Intercellular adhesion molecule-3-Grabbing Non-integrin related; DEGS1, Degenerative spermatocyte homolog 1, lipid desaturase; DEPDC5, DEP domain containing 5; DGCR8, DiGeorge syndrome critical region 8; DHCR7, 7-dehydrocholesterol reductase; EHMT2, Euchromatic histone-lysine methyltransferase 2; eIF-2S1, Eukaryotic translation initiation factor 2 subunit alpha; eIF-2α, Eukaryotic translation initiation factor 2α; E-selectin, CD62 antigen-like family member E; ESR1, Estrogen receptor α; FAS, A cell membrane receptor; FASL, Fas ligand; FCN2, Ficolin-2; FN-γR1, Interferon Gamma Receptor-1; FOXP1, Forkhead box protein P1; GBP2, Interferon-induced guanylate-binding protein 2; GC, Vitamin D binding protein; GEMIN4, Gem (nuclear organelle) associated protein 4; GNLY, Granulysin; HLA-DQ, Human leukocyte antigens, DQ; HLA-A, Human leukocyte antigens, A; HLA-B, Human leukocyte antigens, B; HLA-C, Human leukocyte antigens,C; HLA-DP, Human leukocyte antigens, DP; HLA-DOA, Human leukocyte antigens, DO alpha; HLA-DPA1, Human leukocyte antigens, DP, alpha 1; HLA-DPB1, Human leukocyte antigens, DP, Beta 1; HLA-DQ, Human leukocyte antigens, DQ; HLA-DQA1, Human leukocyte antigens, DQ, alpha 1; HLA-DQB1, Human leukocyte antigens, DQ, Beta 1; HLA-DQB2, Human leukocyte antigens, DQ, Beta 2; HLA-DR, Human leukocyte antigens, DR; HLA-DR, Human leukocyte antigens, DR; HLA-DRA, Human leukocyte antigens, DR alpha; HLA-DRB1, Human leukocyte antigens, DR, Beta 1; HLA-E, Human leukocyte antigen E; HLA-F, Human leukocyte antigen F; HLA-G, Human leukocyte antigen G; HLA-I, Human leukocyte antigens, Ⅰ; HLA-II, Human leukocyte antigens, Ⅱ; HLA-III, Human leukocyte antigens, Ⅲ; HLA-J, Human leukocyte antigen J; IFN- α2, Interferon alpha-2; IFNAR1, Interferon alpha receptor-1; IFNL4, Interferon-λ4; IFNLR1, Interferon lambda receptor 1; IFN-α1, Interferon alpha-1; IFN-α5, Interferon alpha-5; IFN-αR1, Interferon receptor 1; IFN-αR2, Interferon receptor 2; IFN-γ, Interferon gamma; IFN-γR2, Interferon Gamma Receptor-2; IL-10, Interleukin-10; IL-10RB, Interleukin 10 receptor, beta subunit; IL-12, Interleukin-12; IL-12A, Interleukin-12 subunit alpha; IL-12B, Interleukin-12 subunit beta; IL-13, Interleukin-13; IL-15, Interleukin-15; IL-16, Interleukin-16; IL-17, Interleukin-17; IL-17A, Interleukin-17 subunit alpha; IL-17F, Interleukin-17F; IL-18, Interleukin-18; IL-1A, Interleukin-1 alpha; IL-1R1, Interleukin 1 receptor Type 1; IL-1RN, Interleukin 1 receptor antagonist; IL-1β, Interleukin-1β; IL-2, Interleukin-2; IL-21, Interleukin-21; IL-21R, Interleukin-21 receptor; IL-22, Interleukin-22; IL-27, Interleukin-27; IL-27B, Interleukin-27 receptor; IL-28B, Interleukin 28B or interferon-λ 3; IL-29, Interleukin-29; IL-4, Interleukin-4; IL-4RA, Interleukin-4 Receptor Subunit Alpha; IL-6, Interleukin-6; INF-αR1, Interferon receptor 1; INST10, Integrator complex subunit 10; IP-10, Interferon gamma induced protein 10; IRAK1, Interleukin-1 receptor-associated kinase 1; IRF-1, Interferon regulatory factor 1; IRF-3, Interferon regulatory factor 3; IRF5, Interferon regulatory factor 5; ITGAL, Integrin subunit alpha L; JAK, Janus kinase; JAK1, Janus kinase 1; KIF1B, Kinesin family member 1B; KIR, Killer cell immunoglobulin-like receptor; LILRB4, Leukocyte immunoglobulin-like receptor subfamily B member 4; LTA, Lymphotoxin Alpha; LTBR, Lymphotoxin Beta Receptor; MBL, Mannose binding lectin; MBL2, Mannose-binding lectin-2; MCP1, Monocyte chemotactic protein-1; MDR1, Multidrug resistance 1; MIF, Macrophage migration inhibitory factor; MiR, miRNA gene family; MX1, Myxovirus-resistance protein -1; MxA, Myxovirus resistence-1; MYD88, Myeloid differentiation primary response 88; NF-κB, Nuclear factor-kappa B; NF-κB1, Nuclear factor -kappa-B p105 subunit; NF-κB2, Nuclear factor NF-kappa-B p100 subunit; NLRX1, Nucleotide-binding oligomerization domain, leucine rich repeat containing X1; NOTCH4, Neurogenic locus notch homolog protein 4; NTCP, Sodium taurocholate cotransporting polypeptide; OAS, 2'-5'-oligoadenylate synthetase; OAS1, 2'-5'-oligoadenylate synthetase 1; OAS2, 2'-5'-oligoadenylate synthetase 2; OAS3, 2'-5'-oligoadenylate synthetase 3; OASL, 2'-5'-oligoadenylate synthetase-like protein; OCT4, Octamer-binding transcription factor 4; PAK4, P21_activated kinases 4; PAPL, Purple acid phosphatase-like protein; PDCD1, Programmed Cell Death 1; PKR, Protein kinase RNA-activated; PTEN, Phosphatase and tensin homolog; RANTES, Regulated and normal T-cell expressed and secreted; RXRA, Retinoid X Receptor Alpha; SC, Secretory component; SFTA2, Surfactant Associated 2; SLC10A1, Solute carrier family 10 member 1; SOCS3, Suppressor of cytokine signaling 3; SPP1, Secreted phosphoprotein-1; STAT1, Signal transducer and activator of transcription 1; STAT3, Signal transducer and activator of transcription 3; STAT4, Signal transducer and activator of transcription 4; TANK, Tumor necrosis factor receptor-associated factor family member-associated nuclear factor-κB (NF-κB) activator; TCF19, Transcription factor 19; TGFB3, Transforming growth factor beta 3; TGF-α, Transforming growth factor alpha; TGF-βR3, Transforming growth factor beta receptor III; THBS2, Thrombospondin 2; THBS4, Thrombospondin 4; TLR-2, Toll-like receptor 2; TLR-3, Toll-like receptor 3; TLR-4, Toll-like receptor 4; TLR-5, Toll-like receptor 5; TLR-9, Toll-like receptor 9; TMEM2, Transmembrane Protein 2; TNF, Tumor Necrosis Factor; TNFRSF1A, Tumor necrosis factor receptor super family 1A; TNFSF14, Tumor necrosis factor superfamily member 14; TNFSF15, Tumor necrosis factor superfamily member 15; TNF-α, Tumor Necrosis Factor-alpha; TNF-β, Tumor necrosis factor-beta; TP53, Tumor protein p53; TRIM22, Tripartite motif-containing 22; TRPM5, Transient receptor potential cation channel subfamily M, member 5; UBE2L3, Ubiquitin-conjugating enzyme E2 L3; VARS2, Valyl-tRNA synthetase 2; VDBP, Vitamin D-binding protein; VDR, Vitamin D receptor; VIM, Vimentin; ZNRD1, Zinc ribbon domain containing 1
