# Supplementary material for: Host Genetic Determinants of Hepatitis B Virus Infection
Source: Front Genet. 2019 Aug 13;10:696. doi: 10.3389/fgene.2019.00696 (PMC6702792; doi:10.3389/fgene.2019.00696)
Supplement: Supplementary file 2 [file Table_2.doc]

**Supplement Table S2 Selected studies on host genetic factors associated with the susceptibility/clearance of hepatitis B infection.**

| **Genes** | **Gentic determinants**  **SNP/Hap/CNVs** | **Population** | **Case^a^**  **(n)** | **Control^b^**  **(n)** | **Methods** | **Disease association** | **OR (95% CI)** | **P value** | **Reference** |
| --- | --- | --- | --- | --- | --- | --- | --- | --- | --- |
| AGO1 | rs636832 | Chinese | 332 | 352 | PCR | Yes | 0.73 (0.58-0.92) | 0.008 | Shang et al.  2014 |
| AGT | +3889 | Korean | 666 | 429 | PCR | No |  |  | Park et al.  2006 |
|  | -217 |  |  |  |  | No |  |  |  |
|  | -532 |  |  |  |  | No |  |  |  |
|  | -6 |  |  |  |  | No |  |  |  |
| APOBEC3B | deletion | Moroccan | 179 | 216 | PCR | No |  |  | Ezzikouri et al.  2013 |
| APOBEC3G | H186R |  |  |  |  | No |  |  |  |
| AQP2 | rs2878771 | Chinese | 714 | 280 | MassARRAY | No |  |  | Peng et al.  2013 |
| AZIN1 | rs2679757 |  |  |  |  | No |  |  |  |
| BAFF | rs12583006 | Chinese | 386 | 260 | PCR | No |  |  | Han et al.  2017 |
|  | rs9514828 |  |  |  |  | No |  |  |  |
|  | T-A (rs9514828-rs12583006) |  |  |  |  | Yes | 1.67 (1.14-2.46) | 0.009 |  |
| C2 | p.Glu318Asp | Chinese | 1728 | 1636 | PCR | Yes | 1.97 (1.36-2.85) | 2.78×10^-4^ | Zhao et al.  2012 |
|  | rs10947223 | Korean | 977 | 785 | PCR | Yes | 0.56 (0.44-0.71) | 2.60x10^-6^ | Namgoong et al.  2018 |
|  | rs9267673 |  |  |  |  | Yes | 1.76 (1.39-2.22) | 1.26x10^-6^ |  |
|  | rs7746553 |  |  |  |  | Yes | 1.53 (1.27-1.85) | 8.25x10^-6^ |  |
|  | rs9267665 |  |  |  |  | Yes | 2.35 (1.76-3.14) | 1.08x10^-9^ |  |
|  | rs9267677 |  |  |  |  | Yes | 1.77 (1.40-2.25) | 1.36x10^-6^ |  |
|  | rs9279450 |  |  |  |  | Yes | 0.59 (0.47-0.75) | 1.40x10^-5^ |  |
|  | rs78852381 |  |  |  |  | No |  |  |  |
|  | rs6457457 |  |  |  |  | No |  |  |  |
|  | rs117930498 |  |  |  |  | No |  |  |  |
|  | rs497239 |  |  |  |  | No |  |  |  |
|  | rs532091149 |  |  |  |  | No |  |  |  |
|  | rs609061 |  |  |  |  | No |  |  |  |
|  | rs2293751 |  |  |  |  | No |  |  |  |
|  | rs45476300 |  |  |  |  | No |  |  |  |
|  | rs654727 |  |  |  |  | No |  |  |  |
|  | rs58719596 |  |  |  |  | No |  |  |  |
|  | rs9332739 |  |  |  |  | No |  |  |  |
| CCR2 | -64 | Caucasian | 190 | 336 | PCR | No |  |  | Thio et al.  2007 |
|  |  | Korean | 607 | 350 | PCR | No |  |  | Cheong et al.  2007 |
| CCR5 | -2459 | Chinese | 361 | 304 | PCR | No |  |  | Chen et al.  2010 |
|  |  | Caucasian | 190 | 336 | PCR | No |  |  | Thio et al.  2007 |
|  |  | Korean | 607 | 350 |  | No |  |  | Cheong et al. 2007 |
|  | 59029 | Korean | 592 | 106 | MassARRAY | Yes | 1.71 (1.17-2.50) | 0.006 | Ahn et al.  2006 |
|  | 59353 |  |  |  |  | Yes | 1.71 (1.17-2.50) | 0.006 |  |
|  | Δ32 |  |  |  |  | No |  |  |  |
|  |  | Iranian | 357 | 455 | PCR | Yes | 2.3 (1.6-3.3) | < 0.001 | Abdolmohammadi et al.  2016 |
|  |  | Iranian | 60 | 60 | PCR | No |  |  | Khorramdelazad et al.  2013 |
|  |  | Caucasian | 190 | 336 | PCR | Yes | 0.53 (0.33- 0.83) | 0.006 | Thio et al.  2007 |
|  |  | Caucasian | 181 | 316 | PCR | Yes | 0.51 (0.31-0.85) | 0.009 | Thio et al.  2008 |
|  |  | India | 214 | 408 | PCR | Yes |  | 0.005 | Suneetha et al. 2006 |
| CCRL2 | -727 | Caucasian | 190 | 336 | PCR | No |  |  | Thio et al.  2007 |
|  | -502 |  |  |  |  | Yes | 0.50 (0.32-0.80) | 0.003 |  |
|  | -500 |  |  |  |  | No |  |  |  |
| CCRL2-CCR5 | 500T/Δ32 | Caucasian | 190 | 336 | PCR | Yes | 0.44 (0.25-0.78) | 0.005 |  |
|  | 502A/Δ32 |  |  |  |  | Yes | 0.47 (0.26-0.84) | 0.01 |  |
| CD40 | rs1883832 | Chinese | 9114 | 9257 | GWAS | Yes | 1.19 (1.14-1.25) | 2.95× 10^-15^ | Jiang et al.  2015 |
| CDH1 | -472 | Korean | 666 | 429 | PCR | No |  |  | Park et al.  2006 |
|  | -285 |  |  |  |  | No |  |  |  |
|  | +76003 |  |  |  |  | No |  |  |  |
|  | +84762 |  |  |  |  | No |  |  |  |
|  | +86123 |  |  |  |  | No |  |  |  |
| CFB | rs12614 | Chinese | 9114 | 9257 | GWAS | Yes | 1.89 (1.69-2.08) | 1.28 × 10^-34^ | Jiang et al.  2015 |
| COX2 | -162 | Korean | 666 | 429 | PCR | No |  |  | Park et al.  2006 |
|  | 3552 |  |  |  |  | No |  |  |  |
|  | 5789 |  |  |  |  | No |  |  |  |
| CTLA-4 | -1661 (rs4553808) | USA | 189 | 338 | PCR | No |  |  | Thio et al.  2004 |
|  | -658 |  |  |  |  | No |  |  |  |
|  | C-A (-318/+49) | Chinese | 172 | 145 | PCR | Yes | 2.18 (1.52-3.13) | <0.001 | Duan et al.  2011 |
|  | C-G (-318/+49) | Chinese | 172 | 145 | PCR | Yes | 0.64 (0.46-0.88) | 0.006 |  |
|  | +49 (rs231775) | Chinese | 361 | 304 | PCR | Yes | 0.55 (0.34-0.88) | 0.041 | Chen et al.  2010 |
|  |  | German | 323 | 202 | PCR | No |  |  | Schott et al.  2007 |
|  |  | Chinese | 172 | 145 | PCR | Yes | 1.91 (1.01-3.64) | 0.045 | Zhang et al. 2012 |
|  |  | Asian | 1075 | 1321 | MA | Yes | 1.67 (1.30-2.15) | < 0.01 | Huang et al. 2013 |
|  |  | Chinese | 172 | 145 | PCR | Yes | 1.56 (1.13-2.15) | 0.007 | Duan et al. 2011 |
|  |  |  | 1076 | 1294 | MA | Yes | 0.77 (0.68-0.88) | <0.05 | Xu et al.  2013 |
|  |  | USA | 189 | 338 | PCR | Yes | 0.73 (0.56-0.95) | 0.02 | Thio et al.  2004 |
|  |  | Chinese | 1119 |  | PCR | No |  |  | Chen et al. 2014 |
|  | +6230 (rs3087243) | Chinese | 1119 |  | PCR | No |  |  |  |
|  |  | Chinese | 361 | 304 | PCR | Yes | 0.38 (0.20-0.74) | 0.023 | Chen et al.  2010 |
|  |  | USA | 189 | 338 | PCR | Yes | 1.32 (1.01-1.72) | 0.04 | Thio et al.  2004 |
|  | -318 (rs5742909) | German | 323 | 202 | PCR | Yes |  | 0.002 | Schott et al. 2007 |
|  |  | Chinese | 172 | 145 | PCR | Yes | 1.73 (1.02-2.95) | 0.042 | Zhang et al.  2012 |
|  |  | USA | 189 | 338 | PCR | No |  |  | Thio et al.  2004 |
|  |  | Chinese | 1119 |  | PCR | Yes | 1.69 (1.12-2.55) | 0.012 | Chen et al. 2014 |
|  |  | Chinese | 172 | 145 | PCR | No |  |  | Duan et al.  2011 |
|  | -1722 (rs733618) | Chinese | 361 | 304 | PCR | No |  |  | Chen et al.  2010 |
|  |  | USA(White) | 189 | 338 | PCR | Yes | 0.48 (0.25-0.92) | 0.03 | Thio et al.  2004 |
|  | -606 | Chinese | 172 | 145 | PCR | No |  |  | Zhang et al.  2012 |
|  | 8669 |  |  |  |  | Yes | 1.65 (1.15-2.36) | 0.007 |  |
|  | CC/AA (-318 /+8669) |  |  |  |  | Yes | 1.69 (1.07-2.64) | 0.023 |  |
|  | CC/GG (-318 /+8669) |  |  |  |  | Yes | 0.22 (0.059-0.79) | 0.012 |  |
|  | CT/AG (-318 /+8669) |  |  |  |  | Yes | 0.34 (0.16-0.73) | 0.004 |  |
|  | AA /AA (+49/ +8669) |  |  |  |  | Yes | 4.54 (1.69-12.23) | 0.001 |  |
|  | GG /GG (+49/ +8669) |  |  |  |  | Yes | 0.12 (0.014-0.95) | 0.016 |  |
|  | C-A-C-C-G ^1^ | USA | 378 | 676 | PCR | Yes | 0.58 (0.34-0.97) | 0.04 | Thio et al.  2004 |
|  | T/C-A-C-C-G ^2^ |  |  |  |  | Yes | 0.74 (0.57-0.98) | 0.03 |  |
|  | T-A-C-C-A ^3^ |  |  |  |  | Yes | 1.33 (1.01-1.73) | 0.04 |  |
| CXCL10 | rs4256246 | Chinese | 1191 | 273 | PCR | No |  |  | He et al.  2015 |
|  | rs4508917 |  |  |  |  |  |  |  |  |
|  | rs8878 |  |  |  |  |  |  |  |  |
| CXCR1 | S276T | Korean | 607 | 350 | PCR | No |  |  | Cheong et al.  2007 |
| CXCR4 | +2547 | Korean | 666 | 429 | PCR | No |  |  | Park et al.  2006 |
|  | I138I | Korean | 607 | 350 | PCR | No |  |  | Cheong et al. 2007 |
| CYP27B1 | 1260 (rs10877012) | Chinese | 274 | 226 | PCR | Yes | 1.79 (1.18–2.73) | 0.006 | Zhu et al.  2012 |
| DEGS1 | rs4290029 | Chinese | 714 | 280 | MassARRAY | No |  |  | Peng et al.  2013 |
| DEPDC5 | rs1012068 | Chinese | 792 | 857 | MassARRAY | No |  |  | Ma et al.  2014 |
| DGCR8 | rs3757 | Chinese | 332 | 352 | PCR | No |  |  | Shang et al.  2014 |
| EHMT2 | rs35875104 | Korean | 1046 | 2856 | Illumina | Yes | 0.53 (0.42-0.67) | 2.20 × 10^-8^ | Shin et al.  2017 |
|  | rs652888 | Korean | 1371 | 2938 | GWAS | Yes | 1.38 (1.22-1.57) | 7.07×10^-13^ | Kim et al.  2013 |
|  |  | Korean | 1046 | 2856 | Illumina | Yes | 1.58 (1.38-1.79) | 9.90 × 10^-12^ | Shin et al.  2017 |
|  |  | Thai | 685 |  | PCR | No |  |  | Posuwan et al. 2014 |
|  | rs118097312 | Korean | 1046 | 2856 | Illumina | No |  |  | Shin et al.  2017 |
|  | rs149384831 |  |  |  |  | No |  |  |  |
|  | rs114386644 |  |  |  |  | No |  |  |  |
|  | rs535586 |  |  |  |  | No |  |  |  |
|  | rs611572 |  |  |  |  | No |  |  |  |
|  | rs142338646 |  |  |  |  | No |  |  |  |
|  | rs35875104 |  |  |  |  | No |  |  |  |
|  | rs113973079 |  |  |  |  | No |  |  |  |
|  | rs115485095 |  |  |  |  | No |  |  |  |
|  | rs116027812 |  |  |  |  | No |  |  |  |
|  | rs146726232 |  |  |  |  | No |  |  |  |
|  | rs2844458 |  |  |  |  | No |  |  |  |
|  | rs589428 |  |  |  |  | No |  |  |  |
|  | rs570263 |  |  |  |  | No |  |  |  |
|  | rs146903072 |  |  |  |  | No |  |  |  |
|  | rs605203 |  |  |  |  | No |  |  |  |
|  | rs7887 |  |  |  |  | Yes | 0.90 (0.81-0.99) | 0.03 |  |
|  | rs41267090 |  |  |  |  | Yes | 1.29 (1.10-1.52) | 0.003 |  |
|  | rs652888 |  |  |  |  | Yes | 1.58 (1.38-1.79) | 9.90×10^-12^ | Shin et al.  2017 |
|  |  | Chinese | 9114 | 9257 | GWAS | Yes | 1.14 (1.08-1.19) | 9.92×10^-7^ | Jiang et al.  2015 |
| E-selectin | +98 | Han (China) | 150 | 150 | PCR | No |  |  | Cai et al.  2012 |
|  |  | Uygur (China) | 150 | 150 | PCR | No |  |  |  |
|  |  | Chinese | 200 | 200 | PCR | No |  |  | Cai et al.  2015 |
|  |  | Chinese | 367 | 281 | PCR | No |  |  | Wu et al.  2009 |
|  | +561 | Han (China) | 150 | 150 | PCR | Yes | 1.99 (1.08-3.67) | <0.05 | Cai et al.  2012 |
|  |  | Uygur (China) | 150 | 150 | PCR | Yes | 2.18 (1.10-4.32) | <0.025 |  |
|  |  | Chinese | 200 | 200 | PCR | Yes | 2.09 (1.03-4.21) | 0.037 | Cai et al.  2015 |
|  |  | Chinese | 367 | 281 | PCR | No |  |  | Wu et al.  2009 |
|  | AC/GG (+561/+98) | Chinese | 200 | 200 | PCR | Yes | 1.93 (1.04-3.56) | 0.034 | Cai et al.  2015 |
|  | G-A (+561/+98) |  |  |  |  | Yes | 0.51 (0.26-1.00) | 0.047 |  |
|  | G-C (+561/+98) |  |  |  |  | Yes | 1.97 (1.00-3.90) | 0.047 |  |
| ESR1 | 252966 | Chinese | 1277 | 1041 | PCR | No |  |  | Deng et al.  2004 |
|  | T29C |  |  |  |  | Yes | 1.41 (1.17-1.71) | <0.001 |  |
|  | XbaI | Chinese | 112 | 105 | PCR | No |  |  | Liu et al.  2014 |
|  | PvuII |  |  |  |  | Yes | 1.76 (1.32–2.83) | 0.044 |  |
| FAS | -1377 | Iran | 125 | 100 | PCR | No |  |  | Mohammadi et al.  2015 |
|  |  | Brazil | 116 | 235 | PCR | No |  |  | Santana et al.  2013 |
|  |  | Korean | 666 | 429 | PCR | No |  |  | Jung et al.  2007 |
|  | -670 | Brazil | 116 | 235 | PCR | No |  |  | Santana et al.  2013 |
|  |  | Iran | 125 | 100 | PCR | No |  |  | Mohammadi et al.  2015 |
|  |  | Korean | 666 | 429 | PCR | No |  |  | Jung et al.  2007 |
|  | GG/GG (-670/-1377) | Iran | 125 | 100 | PCR | Yes | 0.34 (0.12-0.97) | 0.03 | Mohammadi et al.  2015 |
| FASL | -844 | Iran | 125 | 100 | PCR | No |  |  |  |
|  |  | Brazil | 116 | 235 | PCR | No |  |  | Santana et al.  2013 |
|  |  | Korean | 666 | 429 | PCR | No |  |  | Jung et al.  2007 |
|  | IVS2 (-124) | Brazil | 116 | 235 | PCR | No |  |  | Santana et al.  2013 |
|  | IVS3 (-169) |  |  |  |  | No |  |  |  |
| FOXP1 | rs6789153 | Hong Kong | 216 | 231 | PCR | No |  |  | Lau et al.  2011 |
| GEMIN4 | rs7813 | Chinese | 332 | 352 | PCR | No |  |  | Shang et al.  2014 |
| GNLY | rs11127 | Chinese | 310 | 295 | PCR | No |  |  | Hou et al.  2015 |
|  | rs1866139 |  |  |  |  | No |  |  |  |
|  | rs1866139/ rs11127 |  |  |  |  | No |  |  |  |
| HLA-A | *33:03:01 | Chinese | 896 | 280 | PCR | Yes | 2.31 (1.09-4.90) | 0.025 | Miao et al.  2013 |
|  | *01 |  | 659 | 1652 | MA | No |  |  | Seshasubramanian et al.  2017 |
|  | *02 |  |  |  |  |  |  |  |  |
|  | *03 |  |  |  |  |  |  |  |  |
|  | *11 |  |  |  |  |  |  |  |  |
|  | *24 |  |  |  |  |  |  |  |  |
|  | *26 |  |  |  |  |  |  |  |  |
|  | *29 |  |  |  |  |  |  |  |  |
|  | *30 |  |  |  |  |  |  |  |  |
|  | *31 |  |  |  |  |  |  |  |  |
|  | *32 |  |  |  |  |  |  |  |  |
|  | *33 |  |  |  |  |  |  |  |  |
|  | *68 |  |  |  |  |  |  |  |  |
| HLA-B | *07 |  | 659 | 1652 | MA | Yes | 0.48 (0.29-0.79) | 0.004 |  |
|  |  | Chinese | 230 | 210 | PCR | Yes | 2.18 (1.10-4.31) | 0.023 | Li et al.  2012 |
|  | *08 |  | 659 | 1652 | MA | No |  |  | Seshasubramanian et al.  2017 |
|  | *13 |  |  |  |  | No |  |  |  |
|  | *15 |  |  |  |  | No |  |  |  |
|  | *27 |  |  |  |  | No |  |  |  |
|  | *35 |  |  |  |  | No |  |  |  |
|  | *37 |  |  |  |  | No |  |  |  |
|  | *38 |  |  |  |  | No |  |  |  |
|  | *39 |  |  |  |  | No |  |  |  |
|  | *40 |  |  |  |  | No |  |  |  |
|  | *44 |  |  |  |  | No |  |  |  |
|  | *46 |  |  |  |  | No |  |  |  |
|  | *48 |  |  |  |  | No |  |  |  |
|  |  | Chinese | 230 | 210 | PCR | Yes | 0.33 (0.12-0.92) | 0.026 | Li et al.  2012 |
|  | *51 |  |  |  |  | Yes | 0.48 (0.27-0.86) | 0.012 |  |
|  |  |  | 659 | 1652 | MA | No |  |  | Seshasubramanian et al.  2017 |
|  | *52 |  |  |  |  | No |  |  |  |
|  | *54 |  |  |  |  | No |  |  |  |
|  | *55 |  |  |  |  | No |  |  |  |
|  | *56 |  |  |  |  | No |  |  |  |
|  | *57 |  |  |  |  | No |  |  |  |
|  | *58 |  |  |  |  | Yes | 0.60 (0.38-0.95) | 0.029 |  |
|  | *13:01:01 | Chinese | 896 | 280 | PCR | Yes | 2.62 (1.51-4.54) | 0.0004 | Miao et al.  2013 |
| KIR/HLA-C | 2DL1+//C2+ | Chinese | 364 | 280 | PCR | Yes | 1.75 (1.12-2.73) | 0.013 | Gao et al.  2010 |
|  | 2DL3//C1C1 |  |  |  |  | Yes | 0.56 (0.36-0.88) | 0.011 |  |
|  | KIR2DL3:HLA-C1 homozygotes |  |  |  |  | Yes | 0.59 (0.37-0.93) | 0.024 |  |
|  | 2DL1+//C2- |  |  |  |  | Yes | 0.56 (0.36-0.87) | 0.01 |  |
| HLA-C | Leu-15 | Chinese | 951 | 937 | GWAS | Yes | 1.61 (1.34-1.93) | 3.42×10^-7^ | Zhu et al.  2016 |
|  | rs2853953 | Chinese | 9114 | 9257 | GWAS | Yes | 1.47 (1.35-1.59) | 5.06×10^-20^ | Jiang et al.  2015 |
|  | rs3130542 | Chinese | 5181 | 6610 | GWAS | Yes | 1.33 (1.23-1.44) | 9.49×10^−14^ | Hu et al.  2013 |
|  |  | Chinese | 9114 | 9257 | GWAS | Yes | 1.17 (1.10-1.24) | 8.66×10^-7^ | Jiang et al.  2015 |
|  | w*01 | Chinese | 364 | 280 | PCR | No |  |  | Gao et al.  2010 |
|  | w*02 |  |  |  |  |  |  |  |  |
|  | w*03 |  |  |  |  |  |  |  |  |
|  | w*04 |  |  |  |  |  |  |  |  |
|  | w*05 |  |  |  |  |  |  |  |  |
|  | w*06 |  |  |  |  |  |  |  |  |
|  | w*07 |  |  |  |  |  |  |  |  |
|  | w*08 |  |  |  |  |  |  |  |  |
| HLA-DOA | rs378352 | Chinese | 9114 | 9257 | GWAS | Yes | 1.26 (1.20-1.31) | 1.04× 10^-23^ | Jiang et al.  2015 |
| HLA-DP | rs9277471 | Han (China) | 200 | 200 | PCR | Yes | 0.53 (0.29-0.98) | 0.042 | Xiang et al.  2016 |
|  |  | Uygur (China) | 199 | 200 | PCR | No |  |  |  |
|  | rs9366816 | Taiwan | 1623 | 1065 | GWAS | Yes | 1.43 (1.28-1.60) | 2.58×10^-10^ | Chang et al.  2014 |
| HLA-DP/DQ | T-T-G-A-T ^4^ | Taiwan | 1623 | 1065 | GWAS | Yes | 0.77 (0.68-0.88) | 1.05×10^-4^ | Chang et al.  2014 |
|  | T-T-G-G-T ^5^ |  |  |  |  | Yes | 1.48 (1.33-1.65) | 2.47×10^-12^ |  |
|  | carrying 4-6  variant alleles | Chinese | 1344 | 1344 | PCR | Yes | 0.24 (0.17-0.34) | 3.90×10^-17^ | Hu et al.  2012 |
| HLA-DQ/DP | G-A (rs2856718- rs9275572) | Saudi Arabian | 1672 |  | PCR | Yes |  | 7.60×10^-7^ | Al-Qahtani et al.  2014 |
|  | A-G (rs2856718- rs9275572) |  |  |  |  | Yes |  | 0.0366 |  |
|  | A-A (rs2856718- rs9275572) |  |  |  |  | Yes |  | 0.0284 |  |
| HLA-DPA1 | rs9277341 | Chinese | 521 | 819 | PCR | Yes | 1.77 (1.39-2.25) | 3.99×10^-06^ | Guo et al.  2011 |
|  | rs2301220 |  |  |  |  | Yes | 0.67 (0.56-0.81) | 3.19×10^-05^ |  |
|  | rs2395309 |  |  |  |  | Yes | 0.71 (0.59-0.86) | 0.0005 |  |
|  |  | Chinese | 2805 | 1796 | PCR | Yes | 1.31 (1.17-1.45)  (South of China) | 9.63×10^-7^ | Li et al.  2011 |
|  |  |  |  |  |  | Yes | 1.20 (1.03-1.40)  (North of China) | 0.021 |  |
|  | rs3077 | Argentina | 1440 |  | PCR | Yes | 0.58 (0.43-0.79)  (Central region) | 0.00049 | Trinks et al.  2017 |
|  |  |  |  |  |  |  | 0.61 (0.47-0.79)  (North-Western) | 0.00018 |  |
|  |  | Caucasians | 201 | 235 | PCR | Yes | 5.1 (1.9-13.7) | 0.00093 | Vermehren et al.  2012 |
|  |  | Chinese | 1191 | 273 | PCR | Yes | 0.55 (0.42-0.72) | < 0.0001 | He et al.  2015 |
|  |  | Chinese | 521 | 819 | PCR | Yes | 0.64 (0.53-0.78) | 4.92×10^-06^ | Guo et al.  2011 |
|  |  | Chinese | 3632 | 3232 | MA | Yes | 0.29 (0.13-0.62) | 0.0017 | Yan et al.  2012 |
|  |  | Chinese | 3063 | 1342 | PCR | Yes | 0.72 (0.56-0.92) | 8.00 × 10^-3^ | Zhang et al.  2013 |
|  |  | Chinese | 1742 |  | PCR | Yes | 0.62 (0.47-0.83) | 0.001 | An et al.  2011 |
|  |  | Chinese | 1344 | 1344 | PCR | Yes | 0.81 (0.70-0.95) | 8.30×10^-3^ | Hu et al.  2012 |
|  |  | Chinese | 274 | 353 | PCR | Yes | 1.31 (1.05-1.64) | 0.019 | Fan et al.  2016 |
|  |  | Chinese | 207 | 143 | PCR | Yes | 1.41 (1.09-1.82) | 0.0083 | Wong et al.  2013 |
|  |  | Chinese | 437 | 396 | PCR | Yes | 0.77 (0.63-0.96) | 0.017 | Liao et al.  2014 |
|  |  | Chinese | 9114 | 9257 | GWAS | Yes | 1.45 (1.39-1.52) | 1.15× 10^-53^ | Jiang et al.  2015 |
|  |  | Zhuang  (China) | 177 | 208 | PCR | No |  |  | Wang et al.  2011 |
|  |  | Han (China) | 736 | 782 | PCR | Yes | 0.52 (0.45-0.61) | 3.714×10^-17^ |  |
|  |  | Hong Kong | 203 | 203 |  | Yes | 0.70 (0.50-0.98) | 0.035 | Seto et al.  2013 |
|  |  | Hong Kong | 216 | 231 | PCR | Yes | 1.75 (1.28-2.38) | <0.001 | Lau et al.  2011 |
|  |  | Indonesian | 219 | 226 | PCR | Yes | 0.64 (0.41-0.98) | 0.039 | Wasityastuti et al.  2016 |
|  |  | Japanese | 2086 | 4301 | GWAS | Yes | 0.56 (0.51-0.61) | 2.31×10^–38^ | Kamatani et al.  2009 |
|  |  | Japanese | 2667 | 6496 | GWAS | Yes | 1.87 (1.73-2.01) | 1.57 × 10^-61^ | Mbarek et al.  2011 |
|  |  | Japanese Korean | 1793 |  | GWAS | Yes | 0.43（0.34-0.54） | 1.89×10^-12^ | Nishida et al.  2012 |
|  |  | Korean | 1371 | 2938 | GWAS | Yes | 0.53 (0.48-0.59) | 5.25×10^-39^ | Kim et al.  2013 |
|  |  | Korean | 1046 | 2856 | Illumina | Yes | 0.53 (0.48-0.59) | 3.50×10^-31^ | Shin et al.  2017 |
|  |  | Saudi Arabian | 1672 |  | PCR | Yes | 1.20 (1.01-1.43) | 0.041 | Al-Qahtani et al.  2014 |
|  |  | Thai | 685 |  | PCR | Yes | 0.45 (0.30-0.69) | <0.001 | Posuwan et al.  2014 |
|  |  | Tibetans (China) | 840 | 970 | PCR | Yes | 0.75 (0.63-0.91) | 0.003 | Liao et al.  2015 |
|  |  | Uygurs  (China) | 384 | 470 | PCR | Yes | 0.62 (0.47-0.83) | 0.001 |  |
|  |  | Turkish | 294 | 234 | PCR | No |  |  | Akgöllü et al.  2017 |
|  |  |  | 6505 | 10564 | MA | Yes | 0.629 (0.519-0.763) | 0.001 | Zhang et al.  2013 |
|  |  |  | 18898 | 22419 | MA | Yes | 0.59 (0.55-0.62) | < 10^-5^ | Yu et al.  2015 |
| HLA-DPB1 | rs9277534 | Chinese | 342 | 342 | PCR | Yes | 0.58 (0.46-0.73) | < 0.0001 | Hu et al.  2014 |
|  |  | European- and African-American | 241 | 421 | PCR | Yes | 0.37 | 0.0001 | Thomas et al.  2012 |
|  | *0101 | European- and African-American | 241 | 421 | PCR | Yes | 1.86 (1.16-2.99) | 0.01 |  |
|  |  | Japanese | 2714 | 2450 | PCR | No |  |  | Nishida et al.  2015 |
|  | *0201 | Japanese | 2714 | 2450 | PCR | Yes | 0.71 (0.62-0.81) | 5.42×10^-7^ |  |
|  |  | Japanese, Korean, Hong Kong, Thai | 3167 |  | PCR | Yes | 0.68 (0.58-0.81) | 5.22×10^-6^ | Nishida et al.  2014 |
|  | *0202 | Japanese | 2714 | 2450 | PCR | No |  |  | Nishida et al.  2015 |
|  | *0301 | Japanese |  |  | PCR | No |  |  |  |
|  | *0401 | European- and African-American | 241 | 421 | PCR | Yes | 0.56 (0.38-0.82) | 0.003 | Thomas et al.  2012 |
|  |  | Japanese | 2714 | 2450 | PCR | Yes | 0.39 (0.29-0.53) | 4.12×10^-10^ | Nishida et al.  2015 |
|  | *0402 |  |  |  |  | Yes | 0.49 (0.39-0.62) | 3.51×10^-10^ |  |
|  | *0501 |  |  |  |  | Yes | 1.42 (1.27-1.59) | 4.38×10^-10^ |  |
|  | *0601 |  |  |  |  | No |  |  |  |
|  | *0901 |  |  |  |  | Yes | 1.56 (1.31-1.85) | 3.39×10^-7^ |  |
|  |  | Japanese, Korean, Hong Kong, Thai | 3167 |  | PCR | Yes | 1.97 (1.50-2.59) | 1.36×10^-6^ | Nishida et al.  2014 |
|  | *0201-*0401 | Japanese | 2714 | 2450 | PCR | Yes | 0.26 (0.12-0.58） | 3.96×10^-4^ | Nishida et al.  2015 |
|  | *0201-*0402 |  |  |  |  | Yes | 0.40 (0.24-0.65) | 1.36×10^-4^ |  |
|  | *0201-*0501 |  |  |  |  | Yes | 0.79 (0.64-0.97) | 2.24×10^-2^ |  |
|  | *0401-*0402 |  |  |  |  | Yes | 0.17 (0.05-0.58) | 1.28×10^-3^ |  |
|  | *0401-*0501 |  |  |  |  | Yes | 0.55 (0.34-0.88) | 1.07×10^-2^ |  |
|  | *0402-*0402 |  |  |  |  | Yes | 0.18 (0.04-0.82) | 1.26×10^-2^ |  |
|  | *0402-*0501 |  |  |  |  | Yes | 0.64 (0.46-0.88) | 6.46×10^-3^ |  |
|  | *0501-*0501 |  |  |  |  | Yes | 1.61 (1.31-1.97） | 4.90×10^-6^ |  |
|  | *0501-*0901 |  |  |  |  | Yes | 2.47 (1.89-3.25） | 2.12×10^-11^ |  |
|  | *0501*Others |  |  |  |  | Yes | 1.60 ( 1.10-2.33) | 1.35×10^-2^ |  |
|  | Positions 84-87 | Chinese | 951 | 937 | GWAS | Yes | 0.65 (0.56-0.75) | 2.03×10^-8^ | Zhu et al.  2016 |
|  | rs9277378 | Chinese | 242 | 176 | PCR | Yes | 1.61 (1.26-2.05) | 0.00011 | Wong et al.  2013 |
|  |  | Hong Kong | 203 | 203 | PCR | No |  |  | Seto et al.  2013 |
|  |  | Thai | 449 | 113 | PCR | Yes | 0.47 (0.31-0.72) | <0.001 | Posuwan et al.  2014 |
|  | rs3128917 |  |  |  |  | No |  |  |  |
|  |  | Chinese | 335 | 231 |  | Yes | 1.54 (1.23-1.93) | 0.00017 | Wong et al.  2013 |
|  |  | Hong Kong | 203 | 203 | PCR | No |  |  | Seto et al.  2013 |
|  |  | Chinese | 521 | 819 | PCR | Yes | 1.91 (1.59-2.30) | 5.71×10^-12^ | Guo et al.  2011 |
|  | rs10484569 |  |  |  |  | Yes | 1.60 (1.33-1.93) | 1.65×10^-06^ |  |
|  | rs3117222 |  |  |  |  | Yes | 0.51 (0.42-0.61) | 1.82×10^-12^ |  |
|  | rs9380343 |  |  |  |  | Yes | 0.61 (0.50-0.73) | 5.23×10^-07^ |  |
|  | rs2281388 |  |  |  |  | Yes | 1.66 (1.38-2.01) | 3.37×10^-07^ |  |
|  |  | Chinese | 3063 | 1342 | PCR | Yes | 1.35 (1.05-1.75) | 2.12×10^-2^ | Zhang et al.  2013 |
|  |  | Chinese | 274 | 353 | PCR | No |  |  | Fan et al.  2016 |
|  | rs3135021 | Chinese | 521 | 819 | PCR | Yes | 0.78 (0.64-0.94) | 0.01 | Guo et al.  2011 |
|  |  | Chinese | 3063 | 1342 | PCR | Yes | 0.79 (0.69-0.90) | 6.98×10^-4^ | Zhang et al.  2013 |
|  |  | Chinese | 274 | 353 | PCR | No |  |  | Fan et al.  2016 |
|  |  | Indonesian | 218 | 225 | PCR | No |  |  | Wasityastuti et al.  2016 |
|  | rs9277535 | Chinese | 436 | 370 | PCR | Yes | 0.63 (0.51-0.77) | <0.001 | Liao et al.  2014 |
|  |  | Brazilian | 260 | 260 | PCR | No |  |  | Pereira et al.  2017 |
|  |  | Caucasians | 201 | 235 | PCR | No |  |  | Vermehren et al.  2012 |
|  |  | Chinese | 1742 |  | PCR | Yes | 0.81 | 0.036 | An et al.  2011 |
|  |  | Chinese | 9114 | 9257 | GWAS | Yes | 1.52 (1.45-1.59) | 9.84×10^-71^ | Jiang et al.  2015 |
|  |  | Chinese | 1191 | 273 | PCR | Yes | 0.54 (0.44-0.66) | < 0.0001 | He et al.  2015 |
|  |  | Chinese | 521 | 819 | PCR | Yes | 0.56 (0.47-0.68) | 4.24×10^-9^ | Guo et al.  2011 |
|  |  | Chinese | 4527 | 4059 | PCR+ MA | Yes | 0.26 (0.12-0.54) | 0.0004 | Yan et al.  2012 |
|  |  | Chinese | 3063 | 1342 | PCR | Yes | 0.73 (0.57-0.94) | 1.62×10^-2^ | Zhang et al.  2013 |
|  |  | Chinese | 2805 | 1796 | PCR | Yes | 1.33 (1.20-1.49)  (South of China) | 1.67×10^-7^ | Li et al.  2011 |
|  |  |  |  |  |  | Yes | 1.26 (1.06-1.49)  (North of China) | 8.37×10^-3^ |  |
|  |  | Chinese | 1344 | 1344 | PCR | Yes | 0.60 (0.51-0.70) | 1.69×10^-10^ | Hu et al.  2012 |
|  |  | Chinese | 274 | 353 | PCR | Yes | 1.41 (1.13-1.77) | 0.003 | Fan et al.  2016 |
|  |  | Zhuang  (China) | 83 | 141 | PCR | Yes | 0.597 (0.434-0.822) | 0.001 | Wang et al.  2011 |
|  |  | Han (China) | 528 | 698 | PCR | Yes | 0.69 (0.60-0.80) | 8.828×10^-7^ |  |
|  |  | European- and African-American | 241 | 421 | PCR | Yes | 0.39 | 0.01 | Thomas et al.  2012 |
|  |  | Han (China) | 200 | 199 | PCR | Yes | 1.88 (1.03-3.41) | 0.04 | Xiang et al.  2016 |
|  |  | Uygur Chinese | 198 | 199 | PCR | No |  |  |  |
|  |  | Hong Kong | 216 | 231 | PCR | Yes | 2.10 (1.49-2.96) | <0.001 | Lau et al.  2011 |
|  |  | Indonesian | 686 |  | PCR | Yes | 0.60 (0.38-0.96) | 0.033 | Wasityastuti et al.  2016 |
|  |  | Japanese | 2086 | 4301 | GWAS | Yes | 0.57 (0.52-0.62) | 6.34×10^–39^ | Kamatani et al.  2009 |
|  |  | Japanese | 2667 | 6496 | GWAS | Yes | 1.77 (1.65-1.91) | 2.55 × 10^-54^ | Mbarek et al.  2011 |
|  |  | Korean | 1046 | 2856 | Illumina | Yes | 0.54 (0.48-0.60) | 2.50×10^-32^ | Shin et al.  2017 |
|  |  | Korean | 1371 | 2938 | GWAS | Yes | 0.53 (0.48-0.58) | 3.74×10^-40^ | Kim et al.  2013 |
|  |  | Saudi Arabian | 1672 |  | PCR | Yes | 1.20 (1.00-1.43) | 0.045 | Al-Qahtani et al.  2014 |
|  |  | Taiwan | 1623 | 1065 | GWAS | Yes | 1.59 (1.41–1.79) | 4.87×10^-14^ | Chang et al.  2014 |
|  |  | Tibetans Chinese | 838 | 970 | PCR | Yes | 0.72 (0.60-0.87) | 0.001 | Liao et al.  2015 |
|  |  | Uygurs chinese | 382 | 470 | PCR | Yes | 0.57 (0.42-0.76) | 1.78×10^-4^ |  |
|  |  | Turkish | 294 | 234 | PCR | Yes | 1.35 (1.00-1.82) | 0.048 | Akgöllü et al.  2017 |
|  |  |  | 22065 | 23500 | MA | Yes | 1.54 (1.43-1.66) | < 10^-5^ | Yu et al.  2015 |
|  |  |  | 6458 | 10988 | MA | Yes | 0.66 (0.60-0.73) | 0.028 | Zhang et al.  2013 |
|  | rs9277542 | Argentina | 1440 |  | PCR | Yes | 0.72 (0.55-0.94)  (Central region) | 0.01721 | Trinks et al.  2017 |
|  |  |  |  |  |  |  | 0.59 (0.45-0.78)  (North-Western) | 0.00017 |  |
|  |  | Han (China) | 200 | 200 | PCR | Yes | 0.53 (0.29-0.97) | 0.039 | Xiang et al.  2016 |
|  |  | Uygur (China) | 198 | 200 |  | No |  |  |  |
|  |  | Japanese, Korean | 1793 |  | GWAS | Yes | 0.49 (0.39-0.61） | 9.69×10^-10^ | Nishida et al.  2012 |
|  | G-A-G-A-T-T ^6^ | Chinese | 521 | 819 | PCR | Yes | 1.98 (1.61-2.44) | 1.37×10^-10^ | Guo et al.  2011 |
|  | G-G-G-G-T-C ^7^ |  |  |  |  | Yes | 1.72 (1.23-2.40) | 0.002 |  |
|  | G-A (rs3135021-rs9277535) | Indonesian | 686 |  | PCR | Yes | 0.56 (0.36-0.86) | 0.0087 | Wasityastuti et al.  2016 |
| HLA-DPA1/DPB1 | A-A (rs3077-rs9277535) | Zhuang  (China) | 34 | 69 | PCR | Yes | 0.55 (0.35-0.87) | 0.01 | Wang et al.  2011 |
|  |  | Han (China) | 318 | 438 |  | Yes | 0.53 (0.44-0.63) | 4.40×10^-13^ |  |
|  | G-A (rs3077-rs9277535) | Zhuang  (China) | 49 | 72 |  | Yes | 0.642 (0.430-0.957) | 0.03 |  |
|  |  | Han (China) | 210 | 215 |  | Yes | 0.783 (0.632-0.971) | 0.026 |  |
|  | A-G (rs3077-rs9277535) | Zhuang  (China) | 36 | 29 |  | No |  |  |  |
|  |  | Han (China) | 96 | 186 |  | Yes | 0.414 (0.317-0.540) | 3.430×10^-11^ |  |
|  | A-G (rs3077-rs9277535) | Turkish | 294 | 234 | PCR | Yes | 0.52 ( 0.34-0.80) | 0.003 | Akgöllü et al.  2017 |
|  | C-A (rs3077-rs9277535) | Indonesian | 686 |  | PCR | Yes | 0.57 (0.36-0.92) | 0.021 | Wasityastuti et al.  2016 |
|  | G-G (rs3077-rs9277535) | Saudi Arabian | 1672 |  | PCR | Yes |  | 0.012 | Al-Qahtani et al.  2014 |
|  | rs3077-rs3135021 | Indonesian | 686 |  | PCR | No |  |  | Wasityastuti et al.  2016 |
|  | rs3077-rs3135021-rs9277535* |  |  |  |  |  |  |  |  |
|  | 1. G, A-A(rs2395309-rs9277535) | Chinese | 2805 | 1796 | PCR | Yes | 1.53 (1.24-1.89)  (South of China) | 8.92×10^-5^ | Li et al.  2011 |
|  |  |  |  |  |  | Yes | 1.42 (1.05-1.92)  (North of China) | 0.021 |  |
|  | T-A-T ^8^ | Chinese | 500 | 504 | PCR | Yes | 1.64 (1.21-2.24) | 0.0013 | Wong et al.  2013 |
|  | C-A-T ^9^ |  |  |  |  | Yes | 1.98 (1.35-2.90) | 0.00041 |  |
|  | A1*0103-B1*0401 | Japanese | 2086 | 4301 | GWAS | Yes | 0.57 (0.33-0.96) | 0.002 | Kamatani et al.  2009 |
|  | A1*0103-B1*0402 |  |  |  |  | Yes | 0.52 (0.35-0.75) | 6.00×10^-8^ |  |
|  | A1*0202-B1*0301 |  |  |  |  | Yes | 2.31 (1.39-3.84) | 0.002 |  |
|  | A1*0202-B1*0501 |  |  |  |  | Yes | 1.45 (1.16-1.81) | 5.79×10^-6^ |  |
|  | A-A-C-T ^10^ | Chinese | 521 | 819 | PCR | Yes | 0.54 (0.42-0.69) | 8.73×10^-7^ | Guo et al.  2011 |
|  | G-A-T ^11^ | Hong Kong | 203 | 203 | PCR | Yes | 2.17 ( 1.06-4.45) | 0.034 | Seto et al.  2013 |
|  | A-A-C-C//A-G-T-G-C-C ^12^ | Chinese | 521 | 819 | PCR | Yes | 0.65 (0.49-0.85) | 0.002 | Guo et al.  2011 |
|  | A-A-C-T//A-G-T-G-C-C ^13^ | Chinese | 521 | 819 | PCR | Yes | 0.36 (0.27-0.49) | 3.03×10^-11^ |  |
|  | G-G-T-C//A-G-T-G-C-C ^14^ | Chinese | 521 | 819 | PCR | Yes | 0.56 (0.41-0.75) | 0.0002 |  |
| HLA-DQ | rs9275319 | Chinese | 2341 | 325 | PCR | Yes | 0.63 (0.47-0.85) | 0.003 | Ji et al.  2014 |
|  |  | Chinese | 347 | 375 | MassARRAY | Yes | 0.51 (0.36-0.74) | 0.002 | Fan et al.  2016 |
|  |  | Korean | 958 | 2880 | PCR | Yes | 1.57 (1.37-1.80) | 1.44 ×10^-10^ | Kim et al.  2015 |
|  |  |  | 28347 | 37329 | MA | Yes | 0.64 (0.54-0.76) | < 0.01 | Xu et al.  2018 |
|  | rs9275572 | Chinese | 792 | 505 | PCR | Yes | 0.63 (0.50-0.79) | 7.22×10^-5^ | Zhang et al.  2014 |
|  |  | Chinese | 310 | 295 | PCR | Yes | 1.84 (1.30-2.61) | 0.001 | Hou et al.  2015 |
|  |  | Saudi Arabian | 1672 |  | PCR | Yes | 0.78 (0.66-0.91) | 0.0018 | Al-Qahtani et al.  2014 |
|  |  |  | 28347 | 37329 | MA | Yes | 0.70 (0.62-0.79) | < 0.01 | Xu et al.  2018 |
|  | rs9277346 | Han (China) | 199 | 200 | PCR | Yes | 0.38 (0.17-0.82) | 0.014 | Xiang et al.  2016 |
|  |  | Uygur (China) | 195 | 200 | PCR | Yes | 0.73 (0.55-0.99) | 0.040 |  |
|  | A1*0101-B1*0501 | Japanese | 2667 | 6496 | GWAS | Yes | 0.39 (0.24-0.65) | 1.06 × 10^-5^ | Mbarek et al.  2011 |
|  | A1*0102-B1*0303 |  |  |  |  | Yes | 19.03 (2.53-143.39) | 8.39 × 10^-5^ |  |
|  | A1*0102-B1*0604 |  |  |  |  | Yes | 0.16 (0.09-0.29) | 3.42 × 10^-13^ |  |
|  | A1*0301-B1*0601 |  |  |  |  | Yes | 5.02 (1.87-13.45) | 7.34 × 10^-5^ |  |
|  | A1*0102-B1*0602 |  |  |  |  | Yes | 2.24 (1.28-3.92) | 3.47 × 10^-3^ |  |
|  | A1*0301-B1*0302 |  |  |  |  | Yes | 0.72 (0.50-1.02) | 1.67 × 10^-2^ |  |
|  | A1*0301-B1*0303 |  |  |  |  | Yes | 1.32 (1-1.74) | 7.50 × 10^-3^ |  |
|  | A1*0301-B1*0401 |  |  |  |  | Yes | 0.76 (0.57-1.02) | 2.98 × 10^-3^ |  |
|  | A1*0501-B1*0301 |  |  |  |  | Yes | 0.53 (0.35-0.79) | 1.52 × 10^-3^ |  |
| HLA-DQ/GNLY | rs9275572//rs1866139-rs11127 | Chinese | 310 | 295 | PCR | Yes |  | 0.006-0.007 | Hou et al.  2015 |
| HLA-DQA2 | rs9276370 | Taiwan | 1623 | 1065 | GWAS | Yes | 1.95 (1.62-2.34) | 1.90×10^-12^ | Chang et al.  2014 |
| HLA-DQB1 | *0201 |  | 815 | 731 | MA | Yes | 1.29 (1.02-1.64) | 0.0301 | Huang et al.  2016 |
|  | *0301 |  |  |  |  | Yes | 1.37 (1.12-1.69) | 0.002 |  |
|  | *0302 |  |  |  |  | No |  |  |  |
|  | *0303 | Chinese | 134 | 139 | PCR | Yes | 0.65 (0.52-0.81) | 0.05 | Lin et al.  2006 |
|  |  |  | 815 | 731 | MA | Yes | 0.77 (0.62-0.95) | 0.017 | Huang et al.  2016 |
|  | *0401 |  |  |  |  | No |  |  |  |
|  | *0402 |  |  |  |  |  |  |  |  |
|  | *0501 |  |  |  |  |  |  |  |  |
|  |  | Turkey | 289 | 66 | PCR | Yes | 3.7 (1.5-8.9) | 0.002 | Doganay et al.  2014 |
|  | *0502 | Chinese | 151 | 133 | PCR | Yes | 18 (1.8-190) | <0.05 | Zhu et al.  2007 |
|  |  |  | 815 | 731 | MA | Yes | 1.50 (1.02-2.20) | 0.04 | Huang et al.  2016 |
|  | *0503 | Chinese | 134 | 139 | PCR | Yes | 0.35 (0.21-0.57) | 0.04 | Lin et al.  2006 |
|  |  |  | 815 | 731 | MA | No |  |  | Huang et al.  2016 |
|  | *0601 |  |  |  |  | No |  |  |  |
|  | *0602 |  |  |  |  | No |  |  |  |
|  | *0603 |  |  |  |  | No |  |  |  |
|  | *0604 |  |  |  |  | Yes | 0.38 (0.20-0.74) | 0.003 |  |
|  | rs2856718 | Argentina | 1440 |  | PCR | Yes | 1.69 (1.31-2.18)  (Central region) | 0.00004 | Trinks et al.  2017 |
|  |  |  |  |  |  |  | 1.61 (1.25-2.08)  (North-Western) | 0.00022 |  |
|  |  | Chinese | 1191 | 273 | PCR | Yes | 0.60 (0.45-0.80) | 4.00× 10^-4^ | He et al.  2015 |
|  |  | Chinese | 792 | 507 | PCR | Yes | 0.64 (0.51-0.81) | 2.64×10^-4^ | Zhang et al.  2014 |
|  |  | Chinese | 1344 | 1344 | PCR | Yes | 0.75 (0.64-0.89) | 1.00×10^-3^ | Hu et al.  2012 |
|  |  | Chinese | 2393 | 321 | PCR | No |  |  | Ji et al.  2014 |
|  |  | Chinese | 274 | 353 | PCR | No |  |  | Fan et al.  2016 |
|  |  | Chinese | 9114 | 9257 | GWAS | Yes | 1.28 (1.22-1.33) | 7.35× 10^-28^ | Jiang et al.  2015 |
|  |  | Japanese | 2667 | 6496 | GWAS | Yes | 1.56 (1.45-1.67) | 3.99 × 10^-37^ | Mbarek et al.  2011 |
|  |  | Korean | 1046 | 2856 | Illumina | Yes | 1.61 (1.46-1.78) | 7.10×10^-21^ | Shin et al.  2017 |
|  |  | Korean | 1371 | 2938 | GWAS | Yes | 1.60 (1.46-1.75) | 1.78×10^-24^ | Kim et al.  2013 |
|  |  | Saudi Arabian | 1672 |  | PCR | Yes | 1.35 (1.15-1.59) | 0.0003 | Al-Qahtani et al.  2014 |
|  |  |  | 12053 | 17119 | MA | Yes | 0.75 (0.68-0.82) | < 0.001 | Xu et al.  2017 |
|  |  |  | 28347 | 37329 | MA | Yes | 0.74 (0.67-0.82) | < 0.01 | Xu et al.  2018 |
| HLA-DQB2 | rs7453920 | Argentina | 1440 |  | PCR | Yes | 0.73 (0.56-0.97)  (Central region) | 0.03021 | Trinks et al.  2017 |
|  |  |  |  |  |  | Yes | 0.52 (0.37-0.73)  (North-Western) | 0.00013 |  |
|  |  | Brazilian | 260 | 260 | PCR | No |  |  | Pereira et al.  2017 |
|  |  | Chinese | 445 | 391 | PCR | Yes | 0.55 (0.39-0.77) | <0.001 | Liao et al.  2014 |
|  |  | Chinese | 9114 | 9257 | GWAS | Yes | 2.00 (1.82-2.17) | 1.28 ×10^-60^ | Jiang et al.  2015 |
|  |  | Chinese | 1191 | 273 | PCR | Yes | 0.43 (0.30-0.62) | < 0.0001 | He et al.  2015 |
|  |  | Chinese | 1344 | 1344 | PCR | Yes | 0.60 (0.49-0.73) | 7.61×10^-7^ | Hu et al.  2012 |
|  |  | Chinese | 274 | 353 | PCR | No |  |  | Fan et al.  2016 |
|  |  | Chinese | 5181 | 6610 | GWAS | Yes | 0.53 (0.48–0.59) | 4.93 × 10^−37^ | Hu et al.  2013 |
|  |  | Japanese | 2667 | 6496 |  | Yes | 1.81 (1.62-2.01) | 5.98 × 10^-28^ | Mbarek et al.  2011 |
|  |  | Korean | 1046 | 2856 | Illumina | Yes | 0.50 (0.43-0.58) | 4.60×10^-22^ | Shin et al.  2017 |
|  |  | Korean | 1371 | 2938 | GWAS | Yes | 0.50 (0.44-0.58) | 6.71×10^-26^ | Kim et al.  2013 |
|  |  | Saudi Arabian | 1672 |  | PCR | No |  |  | Al-Qahtani et al.  2014 |
|  |  | Taiwan | 1623 | 1065 | GWAS | Yes | 2.31 (1.87-2.85) | 6.66×10^-15^ | Chang et al.  2014 |
|  |  | Tibetans (China) | 826 | 972 | PCR | Yes | 0.68 (0.52-0.87) | 0.003 | Liao et al.  2015 |
|  |  | Uygurs  (China) | 380 | 470 | PCR | Yes | 0.57 (0.41-0.80) | 0.001 |  |
|  |  |  | 11339 | 17574 | MA | Yes | 0.61 (0.51-0.72) | < 0.001 | Xu et al.  2017 |
|  |  |  | 28347 | 37329 | MA | Yes | 0.64 (0.40-1.02) | 0.06 | Xu et al.  2018 |
|  | rs7756516 | Taiwan | 1623 | 1065 | GWAS | Yes | 1.87 (1.56-2.25) | 1.48×10^-11^ | Chang et al.  2014 |
| HLA-DRB1 | *01 | Chinese | 396 | 400 | PCR | No |  |  | Han et al.  2012 |
|  |  | Brazilian | 64 | 256 | PCR | No |  |  | Correa et al.  2012 |
|  | *03 |  |  |  |  |  |  |  |  |
|  |  | Chinese | 970 | 1185 | MA | Yes | 1.94 (1.23-3.06) | 0.004 | Gao et al.  2015 |
|  |  | Chinese | 396 | 400 | PCR | No |  |  | Han et al.  2012 |
|  |  | Indian | 137 | 150 | PCR | Yes | 0.08 (0.003-0.64) | 0.007 | Fletcher et al.  2011 |
|  | *04 | Brazilian | 64 | 256 | PCR | No |  |  | Correa et al.  2012 |
|  |  | Chinese | 396 | 400 | PCR | Yes | 0.69 (0.492-0.969) | 0.033 | Han et al.  2012 |
|  |  | Indian | 137 | 150 | PCR | No |  |  | Fletcher et al.  2011 |
|  | *07 | Brazilian | 64 | 256 | PCR | No |  |  | Correa et al.  2012 |
|  |  | Chinese | 396 | 400 | PCR | No |  |  | Han et al.  2012 |
|  | *0701 | Indian | 137 | 150 | PCR | Yes | 3.76 (1.84–7.68) | <0.0001 | Fletcher et al.  2011 |
|  | *08 | Brazilian | 64 | 256 | PCR | No |  |  | Correa et al.  2012 |
|  |  | Chinese | 396 | 400 | PCR | No |  |  | Han et al.  2012 |
|  |  | Indian | 137 | 150 | PCR | No |  |  | Fletcher et al.  2011 |
|  | *09 | Brazilian | 64 | 256 | PCR | No |  |  | Correa et al.  2012 |
|  |  | Chinese | 396 | 400 | PCR | Yes | 1.78 (1.34-2.87) | 0.000 | Han et al.  2012 |
|  | *10 | Brazilian | 64 | 256 | PCR | No |  |  | Correa et al.  2012 |
|  |  | Chinese | 396 | 400 | PCR | No |  |  | Han et al.  2012 |
|  |  | Indian | 137 | 150 | PCR | No |  |  | Fletcher et al.  2011 |
|  | *11 | Brazilian | 64 | 256 | PCR | No |  |  | Correa et al.  2012 |
|  |  | Chinese | 396 | 400 | PCR | No |  |  | Han et al.  2012 |
|  |  | Indian | 137 | 150 | PCR | No |  |  | Fletcher et al.  2011 |
|  | *12 | Brazilian | 64 | 256 | PCR | No |  |  | Correa et al.  2012 |
|  |  | Chinese | 396 | 400 | PCR | No |  |  | Han et al.  2012 |
|  |  | Chinese | 230 | 210 | PCR | Yes | 0.61 (0.40-0.93) | 0.021 | Li et al.  2012 |
|  |  | Thailand | 150 | 250 | PCR | No |  |  | Kummee et al.  2007 |
|  | *13 | Brazilian | 64 | 256 | PCR | No |  |  | Correa et al.  2012 |
|  |  | Chinese | 396 | 400 | PCR | Yes | 0.58 (0.36-0.93) | 0.027 | Han et al.  2012 |
|  |  | Chinese | 951 | 937 | GWAS | Yes | 0.28 (0.17-0.47) | 6.27×10^-7^ | Zhu et al.  2016 |
|  |  | Indian | 137 | 150 | PCR | No |  |  | Fletcher et al.  2011 |
|  |  | Chinese | 230 | 210 | PCR | Yes | 3.092 (1.478-6.467) | 0.002 | Li et al.  2012 |
|  |  | Thailand | 150 | 250 | PCR | Yes | 0.04 ( 0.00-0.26) | 0.0008 | Kummee et al.  2007 |
|  | *14 | Brazilian | 64 | 256 | PCR | No |  |  | Correa et al.  2012 |
|  |  | Chinese | 396 | 400 | PCR | No |  |  | Han et al.  2012 |
|  |  | Indian | 137 | 150 | PCR | No |  |  | Fletcher et al.  2011 |
|  | *15 | Brazilian | 64 | 256 | PCR | No |  |  | Correa et al.  2012 |
|  |  | Chinese | 396 | 400 | PCR | No |  |  | Han et al.  2012 |
|  |  | Indian | 137 | 150 | PCR | No |  |  | Fletcher et al.  2011 |
|  | *16 | Brazilian | 64 | 256 | PCR | No |  |  | Correa et al.  2012 |
|  |  | Chinese | 396 | 400 | PCR | No |  |  | Han et al.  2012 |
| HLA-DR13 | rs11752643 | Chinese | 521 | 819 | PCR | No |  |  | Guo et al.  2011 |
| HLA-E | *01:01-*01:03 | Tunisian | 93 | 245 | PCR | No |  |  | Zidi et al.  2016 |
|  | *01:01-*01:03 | Tunisian | 252 | 240 | PCR | No |  |  | Laaribi et al.  2018 |
| HLA-F | *01:02 |  |  |  |  | Yes | 0.07 (0.004-1.28) | 0.01 |  |
|  | *01:03 |  |  |  |  | No |  |  |  |
|  | *01:04 |  |  |  |  | No |  |  |  |
| HLA-G | 14-bp Del | Iranian | 199 | 197 | PCR | Yes | 0.68 (0.51-0.92) | 0.015 | E. Eskandari et al.  2017 |
|  | 14-bp INS/Del |  |  |  |  | Yes | 0.43 (0.28-0.65) | 0.001 |  |
|  |  | Tunisia | 263 | 246 | PCR | No |  |  | Laaribi et al.  2015 |
|  | 14-bp deletion | Brazil | 196 | 202 | PCR | No |  |  | Ferreira et al.  2017 |
| HLA-J | rs400488 | Chinese | 951 | 937 | GWAS | Yes | 1.84 (1.50-2.25) | 3.84×10^-9^ | Zhu et al.  2016 |
| IFN-α1 | rs1332190 | Thailand | 180 | 313 | PCR | Yes | 1.61 (1.12-2.33) | 0.0073 | Kimkong et al.  2013 |
| IFN- α2 | p.Ala120Thr | Chinese | 1728 | 1636 | PCR | Yes | 4.08 (1.85-8.99) | 2.76×10^-5^ | Zhao et al.  2012 |
|  | the deletion from position -305 to-300 | Vietnamese | 344 | 293 | PCR | Yes | 1.9 (1.4-2.7) | < 0.001 | Song et al.  2006 |
| IFN-α5 | rs3758236 | Thailand | 180 | 313 | PCR | No |  |  | Kimkong et al.  2013 |
| PAK4 | rs9676717 | Chinese | 792 | 857 | MassARRAY | No |  |  | Ma et al.  2014 |
| IFNL4 | rs368234815 | Chinese | 397 | 434 | MassARRAY | No |  |  | Fan et al.  2016 |
|  | rs12971396 |  | 390 | 421 |  |  |  |  |  |
|  | rs12979860 |  | 385 | 430 |  |  |  |  |  |
|  | rs8099917 |  | 383 | 418 |  |  |  |  |  |
|  | rs12971396-rs12979860-rs8099917* |  | 383 | 418 |  | No |  |  |  |
| IFN4/HLA-DQ | rs12971396-rs9275319 | Chinese | 1069 |  | MassARRAY | Yes |  | 0.015 | Fan et al.  2016 |
|  | rs12971396-rs12979860-rs9275319 |  |  |  |  | Yes |  | 0.014 |  |
| IFNLR1 | rs4649203 | Chinese | 3128 |  | MassARRAY | Yes | 1.386(1.133-1.697) | 0.001 | Ma et al.  2018 |
|  | rs7525481 |  |  |  |  | Yes | 1.20 (1.02-1.42) | 0.029 |  |
| IFNα5/IFNα1 | A-G (rs3758236-rs1332190) | Thailand | 180 | 313 | PCR | Yes | 0.579 | 0.0378 | Kimkong et al.  2013 |
|  | T-G (rs3758236-rs1332190) | Thailand | 180 | 313 | PCR | Yes | 0.331 | 0.0004 |  |
|  | T-A (rs3758236-rs1332190) | Thailand | 180 | 313 | PCR | Yes | 1.690 | 0.0065 |  |
| IFN-αR1 | -17470 | Chinese | 160 | 124 | PCR | Yes | 2.06 (1.03-4.14) | <0.05 | Xiang et al.  2014 |
|  | -568 | Chinese  Caucasians | 320 | 410 | PCR | Yes | 0.69 (0.52-0.91) | 0.009 | Zhou et al.  2007 |
|  | -408 |  |  |  |  | Yes | 0.56 (0.39-0.79) | 0.001 |  |
|  | (GCC_M_) |  |  |  |  | Yes | 0.69 (0.52-0.91) | 0.009 |  |
|  | GCC_14_ |  |  |  |  | Yes | 0.68 (0.47-0.99) | 0.044 |  |
|  | CTT_L_ |  |  |  |  | Yes | 1.79 (1.27-2.54) | 0.001 |  |
|  | 19158 | Chinese | 328 | 278 | PCR | Yes |  | 0.0067 | Zhou et al.  2009 |
|  | -568 ⁄ 19158 |  |  |  |  | Yes |  | 0.0034 |  |
|  | rs1012335 | Vietnamese | 458 | 160 | PCR | Yes | 2.6 (1.46-4.72) | < 0.001 | Song et al.  2008 |
|  | rs2257167 |  |  |  |  | Yes | 0.54 (0.35-0.84) | 0.004 |  |
| IFN-αR2 | codon 8 | Chinese | 361 | 304 | PCR | No |  |  | Chen et al.  2010 |
|  | rs2229207 | Thailand | 227 | 320 | PCR | Yes | 3.31 (2.11-5.21) | 6.214× 10^-9^ | Romporn et al.  2013 |
|  | rs1051393 | Chinese | 3128 |  | MassARRAY | Yes | 1.22 (1.03-1.45) | 0.024 | Ma et al.  2018 |
|  | rs12233338 |  |  |  |  | Yes | 0.23 (0.069-0.77) | 0.009 |  |
| IFNαR2- IL10RB | C-A (rs2229207-rs2834167) | Thailand | 227 | 320 | PCR | Yes | 6.84 | 0.014 | Romporn et al.  2013 |
|  | C-G (rs2229207-rs2834167) |  |  |  |  | Yes | 3.75 | 0.002 |  |
| IFN-γ | -155 | Chinese | 157 | 210 | PCR | No |  |  | Qi et al.  2005 |
|  | +874 | Chinese | 181 | 272 | PCR | Yes | 2.25 (1.69-2.99) | <0.0001 | Liu et al.  2006 |
|  |  | Chinese | 340 | 100 | PCR | No |  |  | Peng et al.  2007 |
|  |  | Korea | 413 | 201 | PCR | No |  |  | Cheong et al.  2006 |
|  |  |  | 2607 | 2896 | MA | Yes | 1.49 (1.20-1.85) | 0.000 | Sun et al.  2015 |
|  |  | India | 232 | 76 | PCR | No |  |  | Srivastava et al.  2014 |
|  | +2109 | Chinese | 181 | 272 | PCR | No |  |  | Liu et al.  2006 |
|  | –183 | Chinese | 157 | 210 | PCR | Yes | 4.50 (2.23-9.09) | <0.01 | Qi et al.  2005 |
|  | rs2069705 | Chinese | 1191 | 273 | PCR | No |  |  | He et al.  2015 |
|  | A-G (+874/+2109) | Chinese | 181 | 272 | PCR | Yes | 8.14 (4.98-13.30) | <0.0001 | Liu et al.  2006 |
| IFN-γR1 | -611 | Iranian | 200 | 200 | PCR | Yes | 0.16 (0.046-0.58) | 0.006 | Khanizadeh et al.  2012 |
|  | -56 | Korean | 413 | 201 | PCR | No |  |  | Cheong et al.  2006 |
|  |  | Iranian | 200 | 200 | PCR | Yes | 0.47 (0.29-0.75) | 0.002 | Khanizadeh et al.  2012 |
|  | +95 | Korean | 413 | 201 | PCR | No |  |  | Cheong et al.  2006 |
|  | rs10457655 | Chinese | 1191 | 273 | PCR | No |  |  | He et al.  2015 |
|  | rs3799488 |  |  |  |  | Yes | 1.48 (1.13-1.95) | 0.0048 |  |
| IFN-γR2 | Gln64Arg | Korean | 413 | 201 | PCR | No |  |  | Cheong et al.  2006 |
|  | rs2834211 | Chinese | 1191 | 273 | PCR | No |  |  | He et al.  2015 |
|  | rs1059293 |  |  |  |  | Yes | 0.27 (0.10-0.71) | 0.011 |  |
| IRF5 | rs10488630 | Vietnam | 379 | 242 | PCR | No |  |  | Sy et al.  2018 |
|  | rs2280714 |  |  |  |  |  |  |  |  |
|  | rs13242262 |  |  |  |  |  |  |  |  |
|  | rs77416878 |  |  |  |  |  |  |  |  |
| IL-1A | rs3783553 | Chinese | 2011 | 302 | PCR | No |  |  | Du et al.  2014 |
| IL-2 | rs2069772 | Chinese | 1191 | 273 | PCR | No |  |  | He et al.  2015 |
| IL-10 | -1082 | Egyptian | 118 | 119 | PCR | Yes | 0.41 (0.21-0.81) | < 0.01 | Talaat et al.  2014 |
|  |  | Chinese | 361 | 304 | PCR | No |  |  | Chen et al.  2010 |
|  |  | Korean | 412 | 204 | PCR | No |  |  | Cheong et al.  2006 |
|  |  | India | 232 | 76 | PCR | No |  |  | Srivastava et al.  2014 |
|  | -819 | Korean | 412 | 204 | PCR | No |  |  | Cheong et al.  2006 |
|  |  | Egyptian | 118 | 119 | PCR | No |  |  | Talaat et al.  2014 |
|  |  | India | 232 | 76 | PCR | No |  |  | Srivastava et al.  2014 |
|  | -592 | Korean | 412 | 204 | PCR | Yes | 0.40 (0.22-0.73) | 0.003 | Cheong et al.  2006 |
|  |  | Chinese | 361 | 304 | PCR | Yes | 0.67 (0.51-0.94) | 0.048 | Chen et al.  2010 |
|  |  | Chinese | 160 | 124 | PCR | Yes | 2.77 (1.13-4.57) | <0.05 | Xiang et al.  2014 |
|  |  | India | 232 | 76 | PCR | No |  |  | Srivastava et al.  2014 |
|  | A-T (-1082/-819) | Egyptian | 118 | 119 | PCR | Yes | 0.58 (0.35-0.99) | <0.05 | Talaat et al.  2014 |
|  | A-T-A ^15^ | Korean | 412 | 204 | PCR | Yes | 0.40 (0.22-0.73) | 0.003 | Cheong et al.  2006 |
|  | rs1800872 | Chinese | 1191 | 273 | PCR | No |  |  | He et al.  2015 |
|  | rs1800896 |  |  |  |  |  |  |  |  |
| IL-10RB | codon 47 | Chinese | 361 | 304 | PCR | Yes | 1.39 (1.17-1.74) | 0.026 | Chen et al.  2010 |
|  | rs2834167 | Thailand | 227 | 320 | PCR | Yes | 0.45 (0.24-0.83) | 0.006 | Romporn et al.  2013 |
|  |  | Chinese | 792 | 857 | MassARRAY | Yes | 0.61 (0.38-0.86) | 0.02 | Ma et al.  2014 |
| IL-12 | +1188 | Indian | 403 |  | PCR | Yes | 3.30 (1.3-8) | <0.01 | Saxena et al.  2014 |
| IL-12A | rs568408 | Chinese | 293 | 686 | PCR | No |  |  | Tan et al.  2016 |
|  | rs3212227 |  |  |  |  | No |  |  |  |
|  | rs2243115 |  |  |  |  | No |  |  |  |
|  |  | Chinese | 1191 | 273 | PCR | No |  |  | He et al.  2015 |
|  | rs568408 |  |  |  |  |  |  |  |  |
|  | rs2243115-rs568408 | Chinese | 293 | 686 | PCR | No |  |  | Tan et al.  2016 |
| IL-12B | rs3212227 | Chinese | 1191 | 273 | PCR | Yes | 1.38 (1.05-1.81) | 0.021 | He et al.  2015 |
| IL-13 | rs1800925 | Chinese | 206 | 192 | PCR | No |  |  | Deng et al.  2015 |
|  | rs20541 |  |  |  |  | No |  |  |  |
| IL-15 | rs3806798 | Chinese | 234 | 150 | MassARRAY | Yes | 1.70 (1.04-2.76) | 0.033 | Zhang et al.  2014 |
|  | rs12508866 |  |  |  |  | No |  |  |  |
|  | rs1519551 |  |  |  |  | No |  |  |  |
|  | rs12510514 |  |  |  |  | No |  |  |  |
|  | rs6819823 |  |  |  |  | No |  |  |  |
|  | rs2857261 |  |  |  |  | No |  |  |  |
|  | rs10519612 |  |  |  |  | No |  |  |  |
|  | rs10519613 |  |  |  |  | No |  |  |  |
|  | rs1057972 |  |  |  |  | No |  |  |  |
|  | rs10833 |  |  |  |  | No |  |  |  |
|  | rs10519613 | Chinese | 1191 | 273 | PCR | No |  |  | He et al.  2015 |
|  | rs10833 |  |  |  |  | No |  |  |  |
|  | A-T-A-G-G ^16^ | Chinese | 234 | 150 | MassARRAY | Yes |  | 0.022 | Zhang et al.  2014 |
|  | rs10519612-rs10519613  -rs1057972* |  |  |  |  | No |  |  |  |
| IL-16 | rs1131445 | Iranian | 262 | 269 | PCR | Yes | 0.70 (0.49-1.00) | 0.048 | Behelgardi et al.  2015 |
|  | rs11556218 | Chinese | 206 | 270 | PCR | Yes | 0.46 (0.29-0.73) | 0.001 | Li et al.  2011 |
|  |  | Iran | 350 | 394 | PCR | Yes | 1.57 (1.25-1.98) | 0.000 | Romani et al.  2014 |
|  | rs4072111 | Chinese | 206 | 270 | PC | No |  |  | Li et al.  2011 |
|  |  | Iran | 350 | 394 | PCR | Yes | 1.47 (1.04-2.08) | 0.029 | Romani et al.  2014 |
|  | rs4778889 | Chinese | 206 | 270 | PCR | No |  |  | Li et al.  2011 |
|  |  | Iran | 350 | 394 | PCR | No |  |  | Romani et al.  2014 |
| IL-17A | rs2275913 | Chinese | 596 | 612 | PCR | Yes | 0.65 (0.55-0.78) | 2.000×10^-6^ | Ren et al.  2017 |
|  |  | Chinese | 395 | 249 | PCR | No |  |  | Li et al.  2014 |
|  | rs8193036 |  |  |  |  | Yes | 1.88 (1.30-2.72) | 0.001 |  |
|  | 1. A   (rs8193036-rs2275913) |  |  |  |  | Yes | 1.73 (1.04-2.88) | 0.032 |  |
|  | T-G  (rs8193036-rs2275913) |  |  |  |  | Yes | 1.89 (1.13-3.18) | 0.014 |  |
| IL-17F | rs763780 | Chinese | 596 | 612 | PCR | Yes | 0.51 (0.37-0.69) | 1.50×10^-5^ | Ren et al.  2017 |
| IL-18 | -607 | Chinese | 231 | 300 | PCR | No |  |  | Zhang et al.  2005 |
|  |  | Chinese | 129 | 160 | PCR | No |  |  | Lu et al.  2015 |
|  |  | Indian | 271 | 280 | PCR | Yes | 0.71 (0.56-0.90) | 0.005 | Karra et al.  2015 |
|  |  |  | 1200 | 934 | MA | No |  |  | Xia et al.  2014 |
|  | -137 | Chinese | 231 | 300 | PCR | Yes |  | 0.005 | Zhang et al.  2005 |
|  |  | Indian | 271 | 280 | PCR | Yes | 1.36 (1.04-1.77) | 0.025 | Karra et al.  2015 |
|  |  | Chinese | 276 | 254 | PCR | Yes |  | 0.003 | Jiang et al.  2014 |
|  |  | Chinese | 129 | 160 | PCR | Yes | 0.56 (0.35-0.91) | 0.018 | Lu et al.  2015 |
|  |  |  | 699 | 633 | MA | No |  |  | Xia et al.  2014 |
|  | A-C (-607/-137) | Chinese | 258 | 320 | PCR | Yes | 0.51 (0.29-0.88) | 0.015 | Lu et al.  2015 |
|  | rs3882891 | Chinese | 274 | 353 | PCR | No |  |  | Fan et al.  2017 |
|  | rs1946518 | Chinese | 501 | 301 | PCR | Yes | 1.28 (1.05-1.57) | 0.017 | Li et al.  2012 |
|  | rs574424 |  |  |  |  | No |  |  |  |
|  | rs1946518- rs574424 |  |  |  |  | No |  |  |  |
| IL-1β | -511 | Chinese | 190 | 249 | PCR | No |  |  | Zhang et al.  2004 |
|  |  | Indian | 395 | 144 | PCR | No |  |  | Biswas et al.  2013 |
|  | rs1143627 | Chinese | 1191 | 273 | PCR | No |  |  | He et al.  2015 |
|  | rs1143623 |  |  |  |  | No |  |  |  |
|  | rs16944 |  |  |  |  | Yes | 0.67 (0.49-0.92) | 0.016 |  |
|  |  | Chinese | 274 | 353 | PCR | No |  |  | Fan et al.  2017 |
| IL-1R1 | rs13019803 |  |  |  |  | Yes | 0.68 (0.47-0.98) | 0.040 |  |
|  | rs3917267 |  |  |  |  | Yes | 1.74 (1.09-2.77) | 0.02 |  |
|  | rs3732131 |  |  |  |  | No |  |  |  |
|  | rs3917332 |  |  |  |  |  |  |  |  |
|  | rs13019803-rs3917267  -rs3732131-rs3917332* |  |  |  |  | No |  |  |  |
| IL-1RN | allele 2 | Chinese | 190 | 249 | PCR | Yes |  | 0.029 | Zhang et al.  2004 |
|  | genotype1/2 |  |  |  | PCR | Yes |  | 0.025 |  |
|  | VNTR | Indian | 395 | 144 | PCR | No |  |  | Biswas et al.  2013 |
| IL-2 | -330 | Indian | 403 |  | PCR | No |  |  | Saxena et al.  2014 |
| IL-4 | -590 | Indian | 403 |  | PCR | Yes | 0.21 (0.06-0.7) | <0.01 |  |
|  |  | Chinese | 154 | 285 | PCR | No |  |  | Lu et al.  2014 |
|  | -33 |  |  |  |  | No |  |  |  |
|  | rs2227282 |  |  |  | MA | No |  |  | Cui et al.  2013 |
|  | rs2243248 |  |  |  |  |  |  |  |  |
|  | rs2243250 |  |  |  |  |  |  |  |  |
|  | rs2070874 |  |  |  |  |  |  |  |  |
|  | rs2227284 |  |  |  |  |  |  |  |  |
| IL-6 | -572 | Chinese | 219 | 212 | PCR | Yes | 0.61 (0.45-0.84) | 0.002 | Lu et al.  2014 |
|  | - 597 |  |  |  |  | No |  |  |  |
|  | rs1800796 | Chinese | 1191 | 273 | PCR | No |  |  | He et al.  2015 |
| IL-21 | rs12508721 | Chinese | 115 | 127 | PCR | Yes | 0.45 (0.22-0.92) | 0.026 | Zhang et al.  2016 |
|  | rs13143866 | Chinese | 366 | 208 | PCR | No |  |  | Yao et al.  2015 |
|  | rs2221903 | Chinese | 395 | 249 | PCR | Yes | 0.42 (0.26-0.69) | <0.001 | Li et al.  2013 |
|  |  | Chinese | 115 | 127 | PCR | Yes | 1.90 (1.04-3.47) | 0.036 | Zhang et al.  2016 |
|  |  | Chinese | 366 | 208 | PCR | No |  |  | Yao et al.  2015 |
|  | rs907715 | Chinese | 395 | 249 | PCR | No |  |  | Li et al.  2013 |
|  |  | Chinese | 366 | 208 | PCR | No |  |  | Yao et al.  2015 |
|  |  | Chinese | 115 | 127 | PCR | No |  |  | Zhang et al.  2016 |
|  | A-G (rs907715-rs2221903) | Chinese | 395 | 249 | PCR | Yes | 0.41 (0.20-0.87) | 0.017 | Li et al.  2013 |
|  | rs13143866-rs222190-rs907715* | Chinese | 366 | 208 | PCR | No |  |  | Yao et al.  2015 |
| IL-21R | rs3093301 | Chinese | 395 | 249 | PCR | No |  |  | Li et al.  2013 |
|  | T-83C |  |  |  |  | No |  |  |  |
| IL-21/IL-21R | AG/CT+TT ^17^ |  |  |  |  | Yes | 0.24 (0.096-0.58) | 0.001 |  |
| IL-27 | -964 | Chinese | 112 | 105 | PCR | No |  |  | Peng et al.  2013 |
|  | 2905 |  |  |  | PCR | No |  |  |  |
| IL-27B | rs4905 | Chinese | 1191 | 273 | PCR | No |  |  | He et al.  2015 |
|  | rs6613 |  |  |  |  |  |  |  |  |
| IL-28B | rs10903034 | Chinese | 792 | 857 | MassARRAY | No |  |  | Ma et al.  2014 |
|  | rs1188122 | Chinese | 482 | 122 | PCR | No |  |  | Karataylı et al.  2015 |
|  | rs12979860 |  |  |  |  | No |  |  |  |
|  |  | Chinese | 203 | 406 | PCR | No |  |  | Li et al.  2011 |
|  |  | Chinese | 493 | 460 | PCR | No |  |  | Song et al.  2017 |
|  |  | Chinese | 1191 | 273 | PCR | No |  |  | He et al.  2015 |
|  |  | Hong Kong | 203 | 203 | PCR | No |  |  | Seto et al.  2013 |
|  |  | Tibetans (China) | 914 | 972 | PCR | No |  |  | Liao et al.  2015 |
|  |  | Uygurs (China) | 396 | 470 | PCR | No |  |  |  |
|  |  |  | 4028 | 2327 | MA | No |  |  | Lee et al.  2014 |
|  | rs12980275 | Chinese | 203 | 406 | PCR | No |  |  | Li et al.  2011 |
|  |  | Chinese | 493 | 460 | PCR | No |  |  | Song et al.  2017 |
|  |  | Tibetans (China) | 914 | 972 | PCR | No |  |  | Liao et al.  2015 |
|  |  | Uygurs (China) | 396 | 470 | PCR | No |  |  |  |
|  |  | Turkey | 482 | 122 | PCR | Yes | 0.61 (0.40-0.94) | 0.025 | Karataylı et al.  2015 |
|  |  |  | 4028 | 2327 | MA | No |  |  | Lee et al.  2014 |
|  | rs12980602 | Chinese | 792 | 857 | MassARRAY | No |  |  | Ma et al.  2014 |
|  | rs8099917 | Chinese | 482 | 122 | PCR | No |  |  | Karataylı et al.  2015 |
|  |  | Chinese | 203 | 406 | PCR | No |  |  | Li et al.  2011 |
|  |  | Chinese | 493 | 460 | PCR | No |  |  | Song et al.  2017 |
|  |  | Chinese | 792 | 857 | MassARRAY | No |  |  | Ma et al.  2014 |
|  |  | Chinese | 1191 | 273 | PCR | No |  |  | He et al.  2015 |
|  |  | Hong Kong | 203 | 203 | PCR | No |  |  | Seto et al.  2013 |
|  |  | Tibetans (China) | 914 | 972 | PCR | No |  |  | Liao et al.  2015 |
|  |  | Uygurs (China) | 396 | 470 | PCR | No |  |  |  |
|  |  |  | 4028 | 2327 | MA | No |  |  | Lee et al.  2014 |
|  | rs8105790 | Turkey | 482 | 122 | PCR | No |  |  | Karataylı et al.  2015 |
|  | rs12979860-rs8099917-rs12980275* | Chinese | 493 | 460 | PCR | No |  |  | Song et al.  2017 |
|  | C-G(rs12979860-rs8099917) | Hong Kong | 203 | 203 | PCR | Yes | 10.5 (1.33-82.5) | 0.026 | Seto et al.  2013 |
| IL-29 | rs30461 | Chinese | 1191 | 273 | PCR | No |  |  | He et al.  2015 |
| IRAK1 | rs1059702 | Chinese | 1191 | 273 | PCR | No |  |  |  |
|  | rs1059703 |  |  |  |  |  |  |  |  |
| INF-αR1 | -408/-3 C/T | Caucasians | 92 | 91 | PCR | Yes | 1.73 (1.08-2.77) | 0.022 | Karamitros et al.  2015 |
|  | -77 (GT)n |  |  |  |  | Yes |  | <0.001 |  |
|  | -568 |  |  |  |  | Yes | 2.52 (1.65-3.85) | <0.001 |  |
|  | -77GTn ≤8/≤8 |  |  |  |  | Yes | 7.14 (2.70-18.85) | <0.001 |  |
|  | -77 (GT)n≤8/≤8_-568GC |  |  |  |  | Yes | 11.69 | 0.005 |  |
|  | -77(GT)n≤8/≤8_-568CC |  |  |  |  | Yes | 7.56 | 0.001 |  |
| INST10 | rs7000921 | Chinese | 5156 | 4413 | GWAS | Yes | 0.78 | 3.2 × 10^-12^ | Li et al.  2016 |
| IRF-1 | -410 | Korean | 413 | 201 | PCR | No |  |  | Cheong et al.  2006 |
|  | -388 |  |  |  |  | No |  |  |  |
| IRF-3 | rs10415576 | Chinese | 985 | 294 | PCR | No |  |  | Yan et al  2012 |
|  | rs2304204 |  |  |  |  | No |  |  |  |
|  | rs2304206 |  |  |  |  | No |  |  |  |
| KIF1B | rs17401966 | Saudi Arabian | 660 | 584 | PCR | No |  |  | Ahmed Al-Qahtani et al.  2012 |
|  | rs12734551 |  |  |  |  | No |  |  |  |
|  | rs3748578 |  |  |  |  | No |  |  |  |
|  | A-T-A ^18^ | Saudi Arabian | 660 | 584 | PCR | Yes |  | 0.038 |  |
| KIR | AH | Chinese | 401 | 412 | PCR | Yes |  | 0.004 | Lu et al.  2008 |
|  | M |  |  |  |  | Yes |  | 0.004 |  |
|  | G |  |  |  |  | Yes |  | 0.048 |  |
|  | AF |  |  |  |  | Yes |  | 0.014 |  |
|  | AJ |  |  |  |  | Yes |  | 0.006 |  |
|  | FZ1 |  |  |  |  | Yes |  | 0.003 |  |
|  | rs17401966 | Chinese | 1344 | 1344 | PCR | No |  |  | Hu et al.  2012 |
| MBL2 | -550 | Brazil | 102 | 232 | PCR | No |  |  | Filho et al.  2010 |
|  |  | U.S | 189 | 338 | PCR | No |  |  | Thio et al.  2005 |
|  | -221 | Indian | 137 | 150 | PCR | No |  |  | Fletcher et al.  2010 |
|  |  | Brazil | 102 | 232 | PCR | No |  |  | Filho et al.  2010 |
|  |  | USA | 189 | 338 | PCR | Yes | 1.38 (1.01-1.89) | 0.04 | Thio et al.  2005 |
|  |  | Hong Kong | 407 | 484 | PCR | No |  |  | Chong et al.  2005 |
|  | -211 | Chinese | 361 | 304 | PCR | No |  |  | Chen et al.  2010 |
|  | Codon 52 | USA | 189 | 338 | PCR | No |  |  | Thio et al.  2005 |
|  |  | Indian | 137 | 150 | PCR | Yes | 0.25 (0.08-0.80) | 0.02 | Fletcher et al.  2010 |
|  | codon 54 | Korean | 372 | 126 | PCR | No |  |  | Chong et al.  2005 |
|  |  | Chinese | 361 | 304 | PCR | No |  |  | Chen et al.  2010 |
|  |  | Indian | 137 | 150 | PCR | No |  |  | Fletcher et al.  2010 |
|  |  | USA | 189 | 338 | PCR | No |  |  | Thio et al.  2005 |
|  | codon 57 |  |  |  |  | No |  |  |  |
|  |  | Indian | 137 | 150 | PCR | No |  |  | Fletcher et al.  2010 |
|  | codons 52/54/57 | Brazil | 102 | 232 | PCR | No |  |  | Filho et al.  2010 |
|  | rs2120131 | Chinese | 996 | 301 | PCR | No |  |  | Zhang et al.  2013 |
|  | rs4935047 |  |  |  |  |  |  |  |  |
|  | rs7095891 |  |  |  |  |  |  |  |  |
|  | G-C-G-G ^19^ | USA | 189 | 338 | PCR | Yes | 0.73 (0.56-0.94) | 0.02 | Thio et al.  2005 |
|  | C-C-G-G ^20^ |  |  |  |  | Yes | 1.40 (1.03-1.92) | 0.03 |  |
|  | -550/-221/ Exon 1 | Brazil | 204 | 464 | PCR | No | 1.42 (1.00-2.01) | 0.04 | Filho et al.  2010 |
| MBL | -221/codon 52, 54/ 57 | Indian | 137 | 150 | PCR | Yes | 2.28 (1.04-4.99) | 0.035 | Fletcher et al.  2010 |
| MCP1 | -2076 | Korean | 666 | 429 | PCR | No |  |  | Park et al.  2006 |
|  | -2518 |  |  |  |  | Yes | 1.78 (1.20-2.63) | 0.004 |  |
|  |  | Korean | 607 | 350 | PCR | No |  |  | Cheong et al.  2007 |
|  |  | Chinese | 361 | 304 | PCR | Yes | 1.41 (1.13-1.75) | 0.033 | Chen et al.  2010 |
|  | -2518/ -2076 (-/ht2) | Korean | 666 | 429 | PCR | Yes | 1.92 (1.19-3.08) | 0.007 | Park et al.  2006 |
|  | -2518/ -2076 (ht2/ht2) |  |  |  |  | Yes | 1.34 (1.05-1.71) | 0.02 |  |
| MDR1 | +69219 |  |  |  |  | No |  |  |  |
|  | +91191 |  |  |  |  | No |  |  |  |
| MIF | 185 bp | Iranian | 221 | 200 | PCR | No |  |  | Moudi et al.  2016 |
|  | 95 bp |  |  |  |  | No |  |  |  |
|  | rs755622 |  |  |  |  | Yes | 1.55 (1.11-2.16) | 0.009 |  |
|  |  | Chinese | 596 | 612 | PCR | Yes | 0.80 (0.65-0.98) | 0.032 | Wang et al.  2015 |
| miR-26a-1 | rs7372209 | Saudi Arabian | 1352 | 600 | PCR | No |  |  | Al-Qahtani et al.  2017 |
| miR-30a | rs1358379 |  |  |  |  | Yes | 2.082(1.409-3.077) | 0.0002 |  |
| miR-34b/c | rs4938723 | Asian | 6042 | 6834 | MA | No |  |  | Zhou et al.  2015 |
| miR-101-1 | rs7536540 | Korean | 1035 | 404 | PCR | No |  |  | Bae et al.  2012 |
| miR-101-2 | rs12375841 |  |  |  |  | Yes | 1.24 (1.03-1.48) | 0.02 |  |
|  | rs17803780 |  |  |  |  | No |  |  |  |
|  | T-C  (rs12375841-rs17803780) | Korean | 1035 | 404 | PCR | Yes | 1.23 (1.02-1.48) | 0.03 |  |
| MiR-106b-25 | rs999885 | Chinese | 3988 |  | PCR | Yes | 0.79 (0.67-0.93) | 0.004 | Liu et al.  2012 |
|  |  | Asian | 6042 | 6834 | MA | Yes | 1.18 (1.02-1.35) | 0.021 | Zhou et al.  2015 |
| miR-122 | rs3783553 | Asian | 6042 | 6834 | MA | Yes | 1.23 (1.10-1.38) | <0.01 |  |
|  | rs4309483 | Chinese | 1344 | 1344 | PCR | Yes | 0.82 (0.70-0.97) | 0.017 | Liu et al.  2014 |
|  | rs4503880 |  |  |  |  | No |  |  |  |
| miR-146a | rs2910164 | Saudi Arabian | 1352 | 600 | PCR | Yes | 0.65 (0.55-0.78) | <0.0001 | Al-Qahtani et al.  2017 |
|  |  | Asian | 6042 | 6834 | MA | No |  |  | Zhou et al.  2015 |
| miR-149 | rs2292832 |  |  |  |  |  |  |  |  |
|  |  | Saudi Arabian | 1352 | 600 | PCR | No |  |  | Al-Qahtani et al.  2017 |
| miR-196a-2 | rs11614913 | Asian | 6042 | 6834 | MA | Yes | 1.11 (1.03-1.21) | 0.01 | Zhou et al.  2015 |
|  |  | Saudi Arabian | 1352 | 600 | PCR | Yes | 0.72 (0.59-0.88) | 0.001 | Al-Qahtani et al.  2017 |
|  |  | Korean | 1035 | 404 | PCR | No |  |  | Kim et al.  2014 |
|  | rs12304647 | Korean | 1035 | 404 | PCR | No |  |  |  |
|  | rs11614913-rs12304647 | Korean | 1035 | 404 | PCR | No |  |  |  |
| miR-218 | rs11134527 | Asian | 6042 | 6834 | MA | No |  |  | Zhou et al.  2015 |
| miR-219-1 | rs107822 | Korea | 1035 | 404 | PCR | Yes | 0.79 (0.67-0.94) | 0.008 | Cheong et al.  2013 |
|  | rs213210 |  |  |  |  | Yes | 0.83 (0.70-0.98) | 0.03 |  |
|  | rs421446 |  |  |  |  | Yes | 0.73 (0.62-0.87) | 0.0005 |  |
|  | C-A-C ^21^ |  |  |  |  | Yes | 0.83 (0.70-0.98) | 0.03 |  |
|  | T-G-T ^22^ |  |  |  |  | Yes | 0.73 (0.61-0.87) | 0.0004 |  |
|  | C-A-T ^23^ |  |  |  |  | No |  |  |  |
| miR-323b | rs56103835 | Korean | 1035 | 404 | PCR | Yes | 1.29 (1.07-1.56) | 0.009 | Yu et al.  2014 |
| miR-338 | rs62073058 | Korean | 1035 | 404 | PCR | No |  |  | Bae et al.  2012 |
| miR-423 | rs6505162 | Saudi Arabian | 1352 | 600 | PCR | Yes | 1.39 (1.17-1.64) | <0.0001 | Al-Qahtani et al.  2017 |
| miR-492 | rs2289030 |  |  |  |  | Yes | 0.48 (0.33-0.71) | <0.0001 |  |
| miR-499 | rs3746444 |  |  |  |  | No |  |  |  |
|  |  | Asian | 6042 | 6834 | MA | No |  |  | Zhou et al.  2015 |
| miR-604 | rs2368392 | Korea | 1035 | 404 | PCR | Yes | 1.19 (1.00-1.42) | 0.05 | Cheong et al.  2014 |
|  |  | Saudi Arabian | 1352 | 600 | PCR | No |  |  | Al-Qahtani et al.  2017 |
| miR-608 | rs4919510 |  |  |  |  | No |  |  |  |
| miR-let-7c | rs6147150 | Asian | 6042 | 6834 | MA | Yes | 1.35 (1.03-1.77) | 0.031 | Zhou et al.  2015 |
| MxA | -88 | Chinese | 340 | 100 | PCR | Yes | 0.55 (0.76-0.39) | 0.001 | Peng et al.  2007 |
|  |  | Moroccan | 234 | 137 | PCR | No |  |  | Rebbani et al.  2017 |
|  |  | Chinese | 312 | 317 | PCR | No |  |  | Cao et al.  2009 |
|  | -123 | Moroccan | 234 | 137 | PCR | Yes | 2.08 (1.23-3.27) | 0.002 | Rebbani et al.  2017 |
|  |  | Chinese | 312 | 317 | PCR | Yes | 1.63 (1.16-2.27) | 0.004 | Cao et al.  2009 |
|  | G-C (-88/-123) |  |  |  |  | Yes | 0.40 (0.26-0.64) | <0.001 |  |
|  | G-A (-88/-123) |  |  |  |  | Yes | 2.84 (1.12-7.21) | 0.028 |  |
|  | T-A (-88/-123) | Moroccan | 234 | 137 | PCR | Yes |  | <0.001 | Rebbani et al.  2017 |
| MX1 | rs1557370 | Chinese | 1191 | 273 | PCR | No |  |  | He et al.  2015 |
|  | rs2070229 |  |  |  |  |  |  |  |  |
|  | rs469390 |  |  |  |  |  |  |  |  |
|  | rs467960 |  |  |  |  | Yes | 0.68 (0.49-0.94) | 0.022 |  |
| MYD88 | rs7744 | Chinese | 1191 | 273 | PCR | No |  |  |  |
| NF-κB | rs2233406 | Chinese | 3976 | 1669 | PCR | Yes |  | <0.05 | Zhang et al.  2014 |
|  | rs28362491 |  |  |  |  | No |  |  |  |
|  | rs3138053 |  |  |  |  | Yes |  | 0.01 |  |
|  | rs696 |  |  |  |  | No |  |  |  |
| NF-κB1 | rs4648068 | Chinese | 1191 | 273 | PCR | No |  |  | He et al.  2015 |
| NF-κB2 | rs7897947 | Chinese | 1191 | 273 | PCR | No |  |  |  |
| NLRX1 | p.Arg707Cys | Chinese | 1728 | 1636 | PCR | Yes | 2.34 (1.45-3.78) | 5.08×10^-5^ | Zhao et al.  2012 |
| NOTCH4 | rs422951 | Chinese | 9114 | 9257 | GWAS | Yes | 1.27 (1.20-1.35) | 5.33× 10^-16^ | Jiang et al.  2015 |
| NTCP | CNVs | Chinese | 2550 | 2124 | GWAS | No |  |  | Zhang et al.  2017 |
|  | rs17556915 | Chinese | 1221 | 1232 | PCR | No |  |  | Wang et al.  2017 |
|  | rs148467625 | Chinese | 2550 | 2124 | GWAS | No |  |  | Zhang et al.  2017 |
|  | rs2296651 | Chinese | 244 | 189 | PCR | Yes | 2.79 (1.07-7.31) | 0.029 | Li et al.  2014 |
|  |  | Chinese | 2550 | 2124 | GWAS | No |  |  | Zhang et al.  2017 |
|  |  | Chinese | 1619 |  | PCR | Yes | 2.04 (1.39-3.01) | <0.001 | Wu et al.  2018 |
|  |  | Chinese | 1221 | 1232 | PCR | Yes | 0.61 (0.39-0.95) | 0.028 | Wang et al.  2017 |
|  |  | Taiwan | 10017 |  | Affymetrix | Yes | 0.77 (0.59-1.00) | <0.05 | Nfor et al.  2018 |
|  |  | Chinese | 1302 |  | PCR | No |  |  | Su et al.  2016 |
|  |  | Chinese | 648 | 179 | PCR | Yes | 0.32 (0.15-0.68) | 0.003 | An et al.  2018 |
|  |  | Taiwan | 3801 | 3801 | PCR | Yes | 0.13 (0.05-0.34) | <0.001 | Hu et al.  2016 |
|  |  | Chinese | 1899 | 1828 | Mass Array | Yes | 0.36 (0.29-0.44) | 5.7×10^-23^ | Peng et al.  2015 |
|  |  | Chinese | 3650 |  | PCR | Yes | 1.58 (1.05-2.35） | 0.021 | Yang et al.  2016 |
|  |  | Chinese | 14591 | 12396 | MA | Yes | 0.53 (0.29-0.99) | 0.028 | Wang et al.  2017 |
|  |  | Moroccans | 286 | 244 | PCR | No |  |  | Ezzikouri et al.  2017 |
|  | rs4646285 | Chinese | 3650 |  | PCR | No |  |  | Yang et al.  2016 |
|  |  | Chinese | 1619 |  | PCR | Yes | 2.22 (1.24-3.99) | 0.008 | Wu et al.  2018 |
|  | rs4646287 | Chinese | 3650 |  | PCR | Yes | 0.68 (0.51-0.79) | 0.006 | Yang et al.  2016 |
|  |  | Chinese | 581 | 352 | PCR | No |  |  | Su et al.  2014 |
|  |  | Chinese | 14591 | 12396 | MA | No |  |  | Wang et al.  2017 |
|  |  | Chinese | 1302 |  | PCR | No |  |  | Su et al.  2016 |
|  | rs4646296 | Chinese | 581 | 352 | PCR | No |  |  | Su et al.  2014 |
|  |  | Chinese | 1221 | 1232 | PCR | No |  |  | Wang et al.  2017 |
|  | rs7154439 | Chinese | 581 | 352 | PCR | Yes | 0.33 (0.15-0.75) | 0.008 | Su et al.  2014 |
|  |  | Chinese | 14591 | 12396 | MA | No |  |  | Wang et al.  2017 |
|  |  | Chinese | 1302 |  | PCR | No |  |  | Su et al.  2016 |
|  | rs8011311 | Chinese | 1023 | 1467 | PCR | No |  |  | Chen et al.  2016 |
|  | rs7154439 |  |  |  |  | No |  |  |  |
|  | rs111409076 |  |  |  |  | No |  |  |  |
|  | rs9323529 | Chinese | 1221 | 1232 | PCR | No |  |  | Wang et al.  2017 |
|  | rs943276 |  |  |  |  | No |  |  |  |
|  | rs943277 |  |  |  |  | Yes | 2.66 (1.17-6.01) | 0.014 |  |
|  | rs28437822 | Chinese | 3650 |  | PCR | No |  |  | Yang et al.  2016 |
|  | rs11624523 |  |  |  |  | No |  |  |  |
| OCT4 | rs1265163 | Korean | 1046 | 2856 | Illumina | Yes | 1.46 (1.31-1.63) | 4.78×10^-12^ | Shin et al.  2017 |
|  | rs885952 |  |  |  |  | Yes | 1.37 (1.23-1.52) | 4.63×10^-9^ |  |
|  | rs879882 |  |  |  |  | Yes | 1.25 (1.13-1.39) | 2.88×10^-5^ |  |
|  | rs3094191 |  |  |  |  | No |  |  |  |
|  | rs1108746 |  |  |  |  | No |  |  |  |
|  | rs41257954 |  |  |  |  | No |  |  |  |
|  | rs1062630 |  |  |  |  | No |  |  |  |
|  | rs56752756 |  |  |  |  | No |  |  |  |
|  | rs76364340 |  |  |  |  | No |  |  |  |
|  | rs117265349 |  |  |  |  | Yes | 1.67 (1.27-2.19) | 0.0003 |  |
|  | rs3094193 |  |  |  |  | Yes | 0.77 (0.69-0.86) | 7.96×10^-6^ |  |
|  | rs3130503 |  |  |  |  | Yes | 0.72 (0.63-0.82) | 1.00×10^-6^ |  |
|  | rs3130501 |  |  |  |  | Yes | 0.83 (0.73-0.93) | 0.002 |  |
|  | rs3130931 |  |  |  |  | Yes | 0.84 (0.74-0.94) | 0.003 |  |
|  | rs9263800 |  |  |  |  | Yes | 0.65 (0.52-0.82) | 0.0003 |  |
|  | rs3132526 |  |  |  |  | Yes | 0.74 (0.67-0.83) | 1.17×10^-7^ |  |
|  | rs3757349 |  |  |  |  | Yes | 0.76 (0.62-0.92) | 0.006 |  |
|  | rs2394882 |  |  |  |  | Yes | 0.77 (0.69-0.86) | 4.54×10^-6^ |  |
|  | rs13409 |  |  |  |  | Yes | 1.39 (1.25-1.54) | 2.97×10^-10^ |  |
| PAPL | rs423058 | Chinese | 792 | 857 | Mass Array | Yes | 0.73 (0.61-0.87) | 0.0005 | Ma et al.  2014 |
| PTEN | rs34421660 | Iran | 213 | 198 | PCR | No |  |  | Ebrahim Eskandari et al.  2017 |
| RANTES | -403 | Korean | 592 | 106 | MassARRAY | No |  |  | Ahn et al.  2006 |
|  |  | Korean | 607 | 350 | PCR | No |  |  | Cheong et al.  2007 |
|  |  | Caucasian | 181 | 316 | PCR | No |  |  | Thio et al.  2008 |
|  |  | Saudi | 484 | 473 | PCR | Yes |  | 0.001 | Al-Qahtani et al.  2012 |
|  |  | Korean | 666 | 429 | PCR | No |  |  | Park et al.  2006 |
|  | -28 | Korean | 592 | 106 | MassARRAY | No |  |  | Ahn et al.  2006 |
|  |  | Saudi | 484 | 473 | PCR | No |  |  | Al-Qahtani et al.  2012 |
|  |  | Korean | 666 | 429 | PCR | No |  |  | Park et al.  2006 |
|  |  | Caucasian | 181 | 316 | PCR | No |  |  | Thio et al.  2008 |
|  | 524 | Caucasian | 181 | 316 | PCR | No |  |  |  |
|  | In1.1 |  |  |  |  |  |  |  |  |
|  | -403/-28/In1.1/524 |  |  |  |  |  |  |  |  |
| RANTES//CCR5 | -403G//Δ32 |  |  |  |  | Yes | 0.53 (0.31-0.89) | 0.02 |  |
|  | -403A//Δ32 |  |  |  |  | Yes | 0.36 (0.16-0.83) | 0.02 |  |
|  | -28C//Δ32 |  |  |  |  | Yes | 0.55 (0.33-0.91) | 0.02 |  |
|  | In1.1 T//Δ32 |  |  |  |  | Yes | 0.53 (0.32-0.88) | 0.01 |  |
|  | 524T//Δ32 |  |  |  |  | Yes | 0.53 (0.32-0.88) | 0.02 |  |
| SFTA2 | rs3131787 | Korean | 1046 | 2856 | Illumina | No |  |  | Cheong et al.  2015 |
| SOCS3 | rs111033850 | Vietnamese | 878 | 272 | PCR | Yes | 0.6 (0.4-0.8) | <0.0001 | Hoan et al.  2017 |
|  | rs12953258 |  |  |  |  | Yes | 1.3 (1.1-1.6) | 0.03 |  |
|  | C-C (rs111033850-rs12953258) |  |  |  |  | Yes | 0.5 (0.4-0.8) | 0.001 |  |
| SPP1 | -1800 | Korean | 641 | 428 | PCR | Yes | 1.33 (1.02-1.72) | 0.03 | Shin et al.  2007 |
|  | -1627 |  |  |  |  | No |  |  |  |
|  | 4645 |  |  |  |  | No |  |  |  |
|  | 6139 |  |  |  |  | No |  |  |  |
|  | T-T-C-T-A ^24^ |  |  |  |  | Yes | 1.28 (1.04-1.58) | 0.02 |  |
| STAT3 | rs1053004 | Chinese | 567 | 267 | PCR | Yes | 1.40 (1.07-1.85) | 0.019 | Li et al.  2018 |
|  | rs1053005 |  |  |  |  | Yes | 2.52 (1.11-5.74) | 0.046 |  |
|  | T-G (rs1053004-rs1053005) |  |  |  |  | Yes | 0.24 (0.22-0.27) | < 0.001 |  |
|  | C-A (rs1053004-rs1053005) |  |  |  |  | Yes | 1.32 (1.28-1.37) | 0.034 |  |
| STAT4 | rs10168266 | Chinese | 1610 | 1423 | PCR | Yes | 1.19 (1.04-1.36) | 0.013 | Jiang et al.  2016 |
|  | rs11889341 |  |  |  |  | Yes | 1.18 (1.03-1.34) | 0.018 |  |
|  |  | Chinese | 288 | 288 | PCR | Yes | 0.75 (0.59-0.96) | 0.023 | Lu et al.  2015 |
|  | rs3821236 | Chinese | 1610 | 1423 | PCR | No |  |  | Jiang et al.  2016 |
|  | rs1517352 |  |  |  |  | No |  |  |  |
|  | rs4274624 |  |  |  |  | No |  |  |  |
|  | rs11685878 |  |  |  |  | No |  |  |  |
|  | rs7572482 | Chinese | 288 | 288 | PCR | No |  |  | Lu et al.  2015 |
|  |  | Chinese | 1610 | 1423 | PCR | No |  |  | Jiang et al.  2016 |
|  | rs897200 |  |  |  |  | No |  |  |  |
|  | rs4583497 |  |  |  |  | No |  |  |  |
|  | rs1551442 |  |  |  |  | No |  |  |  |
|  | rs2356350 |  |  |  |  | No |  |  |  |
|  | rs7574865 | Korean | 958 | 2880 | PCR | Yes | 1.25 (1.11-1.41) | 0.0002 | Kim et al.  2015 |
|  |  | Tibetans (China) | 786 | 954 | PCR | No |  |  | Liao et al.  2015 |
|  |  | Uygurs (China) | 396 | 419 | PCR | No |  |  |  |
|  |  | Chinese | 1312 |  | PCR | No |  |  | Liao et al.  2014 |
|  |  | Chinese | 1610 | 1423 | PCR | Yes | 1.15 (1.00-1.31) | 0.046 | Jiang et al.  2016 |
|  |  | Chinese | 288 | 288 | PCR | Yes | 0.76 (0.59-0.98) | 0.035 | Lu et al.  2015 |
|  | rs7582694 |  |  |  |  | Yes | 0.69 (0.54-0.88) | 0.011 |  |
|  | rs8179673 | Chinese | 1610 | 1423 | PCR | Yes | 1.14 (0.99-1.30) | 0.047 | Jiang et al.  2016 |
|  |  | Chinese | 288 | 288 | PCR | Yes | 0.67 (0.52-0.86) | 0.002 | Lu et al.  2015 |
|  | G-G-G-C-T ^25^ |  |  |  |  | Yes | 1.37 (1.03-1.82) | 0.031 |  |
|  | C-T-C-T-T ^26^ | Chinese | 1610 | 1423 | PCR | Yes | 0.87 (0.76-0.99) | 0.022 | Jiang et al.  2016 |
| TCF19 | rs1419881 | Korean | 1371 | 2938 | GWAS | Yes | 0.73 (0.66-0.81) | 1.26×10^-18^ | Kim et al.  2013 |
|  |  | Thai | 685 |  | PCR | No |  |  | Posuwan et al.  2014 |
|  |  | Korean | 1046 | 2856 | Illumina | Yes | 0.71 (0.64-0.78) | 2.50×10^-11^ | Shin et al.  2017 |
|  |  | Chinese | 9114 | 9257 | GWAS | Yes | 1.12 (1.08-1.18) | 2.88 ×10^-7^ | Jiang et al.  2015 |
| TGF-α | 88344 | Korean | 653 | 430 | PCR | No |  |  | Kim et al.  2009 |
|  | 106151 |  |  |  |  | Yes | 0.81 (0.67-0.99) | 0.04 |  |
|  | +102906 |  |  |  |  | No |  |  |  |
|  | +103432 |  |  |  |  | No |  |  |  |
|  | +103437 |  |  |  |  | No |  |  |  |
|  | +103461 |  |  |  |  | Yes | 1.48 (1.07-2.03) | 0.02 |  |
|  | +104261 |  |  |  |  | No |  |  |  |
|  | +104802 |  |  |  |  | No |  |  |  |
|  | +106318 |  |  |  |  | No |  |  |  |
|  | A-T-G-T-T-T-T-C-T ^27^ | Korean | 653 | 430 | PCR | Yes | 0.39 (0.22-0.70) | 0.001 |  |
| TGF-β1 | -509 | Indian | 403 |  | PCR | Yes | 10.70 (1.3-85.4) | <0.001 | Saxena et al.  2014 |
|  |  | Iran | 194 | 246 | PCR | No |  |  | Razavi et al.  2014 |
|  | +915 |  |  |  |  | No |  |  |  |
|  | C-C-C ^28^ | Iranian | 178 | 163 | PCR | Yes | 0.66 (0.45-0.96) | 0.028 | Eskandari et al.  2017 |
|  | T-T-T ^29^ |  |  |  |  | Yes | 4.25 (1.49-12.16) | 0.003 |  |
|  | rs1800469 |  |  |  |  | No |  |  |  |
|  | rs1800470 |  |  |  |  | Yes | 0.71 (0.56-0.98) | 0.038 |  |
|  | rs1800472 |  |  |  |  | Yes | 2.53 (1.38-4.61) | 0.002 |  |
| TGF-βR3 | rs1805113 | Korean | 637 | 428 | PCR | Yes | 0.70 (0.54-0.92) | 0.009 | Kim et al.  2011 |
|  | rs1805117 |  |  |  |  | Yes | 0.70 (0.54-0.91) | 0.008 |  |
|  | rs17576372 |  |  |  |  | No |  |  |  |
|  | rs1805110 |  |  |  |  | No |  |  |  |
|  | rs2810904 |  |  |  |  | No |  |  |  |
|  | rs2306888 |  |  |  |  | No |  |  |  |
|  | rs1805112 |  |  |  |  | No |  |  |  |
|  | rs284878 |  |  |  |  | No |  |  |  |
|  | rs1804506 |  |  |  |  | No |  |  |  |
| THBS2 | +36412 | Korean | 666 | 429 | PCR | No |  |  | Park et al.  2006 |
| THBS4 | +30275 |  |  |  |  | No |  |  |  |
| TLR-3 | rs3775291 |  | 944 | 944 | MA | Yes | 1.56 (1.30-1.88) | <0.01 | Geng et al.  2016 |
|  | rs1879026 | Chinese | 172 | 186 | PCR | No |  |  | Huang et al.  2015 |
|  |  | Saudi Arabian | 707 | 600 | PCR | Yes | 0.81 (0.66-1.00) | 0.048 | Al-Qahtani et al.  2012 |
|  | rs3775290 | Chinese | 172 | 186 | PCR | Yes | 0.71 (0.56-0.90) | 0.004 | Huang et al.  2015 |
|  | rs3775296 | Chinese | 1191 | 273 | PCR | No |  |  | He et al.  2015 |
|  | rs5743311 | Saudi Arabian | 707 | 600 | PCR | No |  |  | Al-Qahtani et al.  2012 |
|  | rs5743312 |  |  |  |  | No |  |  |  |
|  | rs5743313 |  |  |  |  | No |  |  |  |
|  | rs5743314 |  |  |  |  | No |  |  |  |
|  | rs5743315 |  |  |  |  | No |  |  |  |
|  | rs111611328 |  |  |  |  | No |  |  |  |
|  | rs78726532 |  |  |  |  | No |  |  |  |
|  | rs184322913 |  |  |  |  | No |  |  |  |
|  | G-C-G-A ^30^ |  |  |  |  | Yes |  | 0.0339 |  |
|  | 1. T   (rs1879026-rs3775290) | Chinese | 344 | 372 | PCR | Yes | 0.65 (0.47-0.90) | 0.01 | Huang et al.  2015 |
| TLR-4 | rs1927911 | Chinese | 714 | 280 | MassARRAY | No |  |  | Peng et al.  2013 |
|  | rs960312 |  |  |  |  | No |  |  |  |
|  | rs11536889 | Chinese | 1191 | 273 | PCR | No |  |  | He et al.  2015 |
|  | rs1927907 |  |  |  |  | No |  |  |  |
|  | rs4986790 | Brazil | 49 | 299 | PCR | No |  |  | Pires-Neto et al.  2015 |
|  | rs4986791 |  |  |  |  | No |  |  |  |
| TLR-5 | rs2072493 | Chinese | 636 | 273 | PCR | No |  |  | Cao et al.  2017 |
|  | rs5744174 |  |  |  |  | No |  |  |  |
|  |  | Chinese | 1191 | 273 | PCR | No |  |  | He et al.  2015 |
| TLR-7 | CNVs | Chinese | 623 | 300 | AccuCopy | Yes | 0.33 ( 0.23-0.47) | < 0.001 | Li et al.  2017 |
|  | rs179009 | Chinese | 612 | 293 | PCR | No |  |  | Zhu et al.  2017 |
|  | rs179010 |  |  |  |  | Yes | 0.82 (0.68-0.98) | 0.029 |  |
|  | rs2074109 |  |  |  |  | No |  |  |  |
|  | C-T-A ^31^ |  |  |  |  | Yes | 1.74 (1.28-2.36) | 0.000 |  |
|  | T-T-A ^32^ |  |  |  |  | Yes | 0.50 (0.36-0.70) | <0.0001 |  |
| TLR-9 | rs352140 | Chinese | 1191 | 273 | PCR | Yes | 0.70 (0.53-0.91) | 0.0088 | He et al.  2015 |
| TMEM2 | p.Ser1254Asn | Chinese | 1728 | 1636 | PCR | Yes | 2.45 (1.89-3.16) | <1.0×10^-7^ | Zhao et al.  2012 |
| TMEM2/IFNA2/NLRX1/C2 | p.Ser1254-Asn//p.Ala120Thr// p.Arg707Cys// p.Glu318Asp | Chinese | 1487 | 1611 | PCR | Yes |  | <2.0×10^-16^ |  |
| TNF | rs1799964 | Indian | 137 | 150 | PCR | Yes | 1.57 (1.09-2.27) | 0.01 | Fletcher et al.  2011 |
|  | rs1800630 |  |  |  |  | Yes | 1.99 (1.30-3.05) | <0.01 |  |
|  | rs361525 |  |  |  |  | No |  |  |  |
|  | rs1800629 |  |  |  |  | No |  |  |  |
|  | rs1799724 |  |  |  |  | No |  |  |  |
|  | C-A-C-G-G ^33^ |  |  |  |  | Yes |  | 0.0004 |  |
| TNF-α | rs3091256 | India | 214 | 408 | PCR | No |  |  | Suneetha et al.  2006 |
|  | -1031 | Chinese | 196 | 143 | PCR | No |  |  | Du et al.  2006 |
|  |  | Korean | 1109 | 291 | PCR | No |  |  | Kim et al.  2003 |
|  | -863 | Chinese | 196 | 143 | PCR | Yes | 1.79 (1.00-3.21) | 0.04 | Du et al.  2006 |
|  |  | Thailand | 150 | 250 | PCR | Yes | 0.54 (0.35-0.84) | 0.03 | Kummee et al.  2007 |
|  |  | Korean | 1109 | 291 | PCR | Yes | 1.52 (1.16-2.00) | 0.003 | Kim et al.  2003 |
|  |  |  | 5245 | 3181 | MA | No |  |  | Zheng et al.  2012 |
|  |  | Chinese | 171 | 227 | PCR | No |  |  | Xu et al.  2013 |
|  | -857 | Chinese | 361 | 304 | PCR | No |  |  | Chen et al.  2010 |
|  |  | Chinese | 196 | 143 | PCR | Yes | 0.48 (0.24-0.96) | 0.02 | Du et al.  2006 |
|  |  | Korean | 1109 | 291 | PCR | No |  |  | Kim et al.  2003 |
|  |  | Asian | 4929 | 2702 | MA | Yes | 0.82 ( 0.71-0.95) | 0.008 | Shi et al.  2012 |
|  | -308 | Chinese | 361 | 304 | PCR | Yes | 1.76 (1.16-2.67) | 0.029 | Chen et al.  2010 |
|  |  | Chinese | 160 | 124 | PCR | No |  |  | Xiang et al.  2014 |
|  |  | Thailand | 150 | 250 | PCR | No |  |  | Kummee et al.  2007 |
|  |  | Korean | 1109 | 291 | PCR | Yes | 0.57 (0.37-0.89) | 0.01 | Kim et al.  2003 |
|  |  | Iranian | 100 | 180 | PCR | No |  |  | Somi et al.  2006 |
|  |  | Chinese | 171 | 227 | PCR | Yes | 0.43 (0.19-0.92) | 0.03 | Xu et al.  2013 |
|  |  | Korean | 412 | 204 | PCR | Yes | 0.58 (0.34-0.97) | 0.039 | Cheong et al.  2006 |
|  |  | Chinese | 196 | 143 | PCR | No |  |  | Du et al.  2006 |
|  | -238 | Chinese | 361 | 304 | PCR | No |  |  | Chen et al.  2010 |
|  |  | Thailand | 150 | 250 | PCR | No |  |  | Kummee et al.  2007 |
|  |  | Korean | 1109 | 291 | PCR | No |  |  | Kim et al.  2003 |
|  |  | Korean | 412 | 204 | PCR | No |  |  | Cheong et al.  2006 |
|  |  | Chinese | 196 | 143 | PCR | Yes | 3.61 (1.01-13.98) | 0.02 | Du et al.  2006 |
|  |  | Asian | 5245 | 3181 | MA | No |  |  | Zheng et al.  2012 |
|  |  | European | 5245 | 3181 | MA | Yes | 2.22 (1.07-4.58) | 0.032 | Zheng et al.  2012 |
|  | -163 | Korean | 1109 | 291 | PCR | No |  |  | Kim et al.  2003 |
|  | G-G (-308/-238) | Korean | 412 | 204 | PCR | Yes | 0.53 (0.34-0.82) | 0.005 | Cheong et al.  2006 |
|  | A-C (-308/-863) | Chinese | 171 | 227 | PCR | No |  |  | Xu et al.  2013 |
|  | G-C (-308/-863) |  |  |  |  | No |  |  |  |
|  | G-A (-308/-863) |  |  |  |  | No |  |  |  |
|  | T-C-C-G-G-G ^34^ | Korean | 1109 | 291 | PCR | Yes | 0.78 (0.67-0.92) | 0.003 | Kim et al.  2003 |
|  | C-A-C-G-G-G ^35^ |  |  |  |  | Yes | 1.42 (1.09-1.87) | 0.01 |  |
|  | G-G-C-C-T ^36^ | Chinese | 196 | 143 | PCR | Yes | 0.59 (0.36-0.96) | 0.03 | Du et al.  2006 |
|  | G-G-C-A-T ^37^ |  |  |  |  | Yes | 2.85 (1.57-5.19) | 0.0001 |  |
|  | G-G-T-A-T ^38^ |  |  |  |  | Yes | 4.15 (1.35-13.54) | 0.004 |  |
| TNF-β | rs909253 | India | 214 | 408 | PCR | No |  |  | Suneetha et al.  2006 |
| TP53 | 16-bp INS/Del | Iran | 213 | 198 | PCR | No |  |  | Ebrahim Eskandari et al.  2017 |
| TRIM22 | SPRY domain-7C/T | Chinese | 293 | 472 | PCR | No |  |  | Zhao et al.  2014 |
|  | -364 |  |  |  |  | Yes | 1.97 (1.10-3.75) | 0.0049 |  |
|  | -336 |  |  |  |  | No |  |  |  |
| TRPM5 | rs886277 | Chinese | 714 | 280 | MassARRAY | No |  |  | Peng et al.  2013 |
| UBE2L3 | rs2266959 | Chinese | 1560 | 1344 | PCR | Yes | 0.80 (0.68-0.93) | <0.05 | Liu et al.  2018 |
|  | rs4821116 | Chinese | 5181 | 6610 | GWAS | Yes | 0.82 (0.77-0.87) | 1.71×10^−12^ | Hu et al.  2013 |
|  |  | Chinese | 1560 | 1344 | PCR | Yes | 0.80 (0.69-0.93) | <0.05 | Liu et al.  2018 |
|  |  | Chinese | 9114 | 9257 | GWAS | No |  |  | Jiang et al.  2015 |
| VARS2 | rs1043483 | Korean | 1046 | 2856 | Illumina | Yes | 0.69 (0.60-0.79) | 1.7×10^-7^ | Cheong et al.  2015 |
|  | rs1264295 |  |  |  |  | Yes | 0.69 (0.55-0.87) | 0.002 |  |
|  | rs1264300 |  |  |  |  | No |  |  |  |
|  | rs140184279 |  |  |  |  | No |  |  |  |
|  | rs184436050 |  |  |  |  | No |  |  |  |
|  | rs2074506 |  |  |  |  | No |  |  |  |
|  | rs2249464 |  |  |  |  | Yes | 0.71 (0.62-0.80) | 1.3×10^-7^ |  |
|  | rs6926224 |  |  |  |  | No |  |  |  |
|  | rs9357103 |  |  |  |  | No |  |  |  |
|  | rs9394021 |  |  |  |  | Yes | 0.78 (0.70-0.86) | 1.7×10^-6^ |  |
|  | rs2517459 |  |  |  |  | Yes | 0.56 (0.47-0.67) | 1.7×10^-10^ |  |
|  | rs2532929 |  |  |  |  | No |  |  |  |
|  | rs2532932 |  |  |  |  | Yes | 0.57 (0.48-0.69) | 2.9×10^-9^ |  |
| VDR | Codon 352 | Chinese | 361 | 304 | PCR | No |  |  | Chen et al.  2010 |
|  | rs7975232 | India | 214 | 408 | PCR | No |  |  | Suneetha et al.  2006 |
|  | Taq I | India | 214 | 408 | PCR | No |  |  | Suneetha et al.  2006 |
|  |  | Chinese | 274 | 226 | PCR | No |  |  | Zhu et al.  2012 |
|  |  |  | 4218 | 2298 | MA | No |  |  | He et al.  2018 |
|  | ApaI |  |  |  |  | No |  |  |  |
|  | BsmI |  |  |  |  | No |  |  |  |
|  | FokI |  |  |  |  | Yes | 1.24(1.12-1.38) | <0.01 |  |
| VIM | -510 | Korean | 666 | 429 | PCR | No |  |  | Park et al.  2006 |
|  | +6750 |  |  |  |  | No |  |  |  |
| ZNRD1 | rs3757328 | Chinese | 1344 | 1344 | MassARRAY | Yes | 0.81 (0.70-0.95) | 0.008 | Wen et al.  2015 |
|  | rs6940552 |  |  |  |  | No |  |  |  |
|  | rs9261204 |  |  |  |  | No |  |  |  |
|  | G-G-A ^39^ |  |  |  |  | Yes | 0.83 (0.71-0.96) | 0.013 |  |

**Note:**

1. Case: number of chronic HBV-infected patients included in the studies; b. Control: healthy controls (HBsAg negative, Anti-HBc negative or positive); in a minority of the studies, the numbers of HBV infected patients and controls were not separately given. For these studies, the total number of subjects included were given in the case column.; SNP, single nucleotide polymorphism; CNVs, copy number variations; HBV, hepatitis B virus; OR (95%CI), odds ratio (95% confidence interval); Yes, positive result reported; No, not statistical significance; GWAS, genome-wide association study; PCR, polymerase chain reaction-based research methods; MALDI-TOF-MS, Matrix-Assisted Laser Desorption/ Ionization Time of Flight Mass Spectrometry; MA, Meta-Analysis; Population, including race or region; VNTR, variable nucleotide tandem repeats.

**Haplotypes**

1. C-A-C-C-G, -1722 /-1661/-658/-319 /+49

2.T/C-A-C-C-G, -1722 /-1661/-658/-319 /+49

3.T-A-C-C-A, -1722 /-1661/-658/-319 /+49

4.T-T-G-A-T, rs9276370-rs7756516-rs7453920-rs9277535-rs9366816

5.T-T-G-G-T, rs9276370-rs7756516-rs7453920-rs9277535-rs9366816

6.G-A-G-A-T-T, rs9277535-rs10484569-rs3128917-rs2281388-rs3117222-rs9380343

7.G-G-G-G-T-C, rs9277535-rs10484569-rs3128917-rs2281388-rs3117222-rs9380343

8.T-A-T, rs3077-rs9277378-rs3128917

9.C-A-T, rs3077-rs9277378-rs3128917

10.A-A-C-T, rs2395309-rs3077-rs2301220-rs9277341

11.G-A-T, rs3077-rs9277378-rs3128917

12.A-A-C-C//A-G-T-G-C-C, rs2395309-rs3077-rs2301220-rs9277341//rs9277535-rs10484569-rs3128917-rs2281388-rs3117222-rs9380343

13.A-A-C-T//A-G-T-G-C-C, rs2395309-rs3077-rs2301220-rs9277341//rs9277535-rs10484569-rs3128917-rs2281388-rs3117222-rs9380343

14.G-G-T-C//A-G-T-G-C-C, rs2395309-rs3077-rs2301220-rs9277341//rs9277535-rs10484569-rs3128917-rs2281388-rs3117222-rs9380343

15.A-T-A, -1082/-819/-592

16.A-T-A-G-G, rs3806798-rs12508866-rs1519551-rs6819823-rs2857261

17.AG/CT+TT, rs2221903-rs3093301

18.A-T-A , rs17401966-rs12734551-rs3748578

19.G-C-G-G, rs7096206-rs5030737-rs1800450-rs1800451

20.C-C-G-G, rs7096206-rs5030737-rs1800450-rs1800451

21.C-A-C, rs421446-rs107822-rs213210

22.T-G-T, rs421446-rs107822-rs213210

23.C-A-T, rs421446-rs107822-rs213210

24.T-T-C-T-A, -1800/-1627/+4645/+5806/+6139

25.G-G-G-C-T, rs7574865-rs7572482-rs7582694- rs11889341-rs8179673

26.C-T-C-T-T, rs8179673-rs7574865-rs4274624-rs11889341-rs10168266

27.A-T-G-T-T-T-T-C-T, +88344/+102906/+103432/+103437/+103461/+104261/+104802/+106151/+106318

28.C-C-C, +869/-509/+11929

29.T-T-T, +869/-509/+11929

30.G-C-G-A, rs1879026- rs5743313-rs5743314-rs5743315

31.C-T-A, rs179010- rs2074109-rs179009

32.T-T-A, rs179010-rs2074109-rs179009

33.C-A-C-G-G, rs1799964-rs1800630-rs1799724-rs1800629-rs361525

34.T-C-C-G-G-G, -1031/-863/-857/-308/-238/-163

35.C-A-C-G-G-G, -1031/-863/-857/-308/-238/-163

36.G-G-C-C-T, -238/-308/-857-863/-1031

37.G-G-C-A-T, -238/-308/-857-863/-1031

38.G-G-T-A-T, -238/-308/-857-863/-1031

39.G-G-A, rs3757328-rs6940552-rs9261204

**References:**

Abdolmohammadi, R., Shahbazi, A. S., Khosravi, A. and Shahbazi, M. (2016). CCR5 Polymorphism as a Protective Factor for Hepatocellular Carcinoma in Hepatitis B Virus-Infected Iranian Patients. *Asian Pac J Cancer Prev* 17, 4643-4646. doi:10.7314/APJCP.2016.17.10.4643.

Ahn, S. H., Kim, D. Y., Chang, H. Y., Hong, S. P., Shin, J., Kim, Y. S. et al. (2006). Association of genetic variations in CCR5 and its ligand, RANTES with clearance of hepatitis B virus in Korea. *Journal of Medical Virology* 78, 1564-1571. doi:10.1002/jmv.20739.

Akgöllü, E., Bilgin, R., Akkız, H., Ülger, Y., Kaya, B. Y., Karaoğullarından, Ü. et al. (2017). Association between chronic hepatitis B virus infection and HLA-DP gene polymorphisms in the Turkish population. *Virus Research* 232, 6-12. doi:10.1016/j.virusres.2017.01.017.

Al-Qahtani, A., Al-Ahdal, M., Abdo, A., Sanai, F., Al-Anazi, M., Khalaf, N. et al. (2012). Toll-like receptor 3 polymorphism and its association with hepatitis B virus infection in Saudi Arabian patients. *Journal of Medical Virology* 84, 1353-1359. doi:10.1002/jmv.23271.

Al-Qahtani, A., Al-Anazi, M., Viswan, N. A., Khalaf, N., Abdo, A. A., Sanai, F. M. et al. (2012). Role of Single Nucleotide Polymorphisms of KIF1B Gene in HBV-Associated Viral Hepatitis. *PLoS ONE* 7, e45128. doi:10.1371/journal.pone.0045128.

Al-Qahtani, A., Alarifi, S., Al-Okail, M., Hussain, Z., Abdo, A., Sanai, F. et al. (2012). RANTES gene polymorphisms (-403G>A and -28C>G) associated with hepatitis B virus infection in a Saudi population. *Genetics and Molecular Research* 11, 855-862. doi:10.4238/2012.April.10.1.

Al-Qahtani, A. A., Al-Anazi, M. R., Abdo, A. A., Sanai, F. M., Al-Hamoudi, W., Alswat, K. A. et al. (2014). Association between HLA variations and chronic hepatitis B virus infection in Saudi Arabian patients. *PLoS One* 9, e80445. doi:10.1371/journal.pone.0080445.

Al-Qahtani, A. A., Al-Anazi, M. R., Nazir, N., Wani, K., Abdo, A. A., Sanai, F. M. et al. (2017). Association of single nucleotide polymorphisms in microRNAs with susceptibility to hepatitis B virus infection and HBV-related liver complications: A study in a Saudi Arabian population. *Journal of Viral Hepatitis* 24, 1132-1142. doi:10.1111/jvh.12749.

An P, Z. Z. W. C. (2018). The loss-of-function S267F variant in HBV receptor NTCP reduces human risk to HBV infection and disease progression. *The Journal of Infectious Diseases* doi:10.1093/infdis/jiy355/5037696.

An, P., Winkler, C., Guan, L., O'Brien, S. J. and Zeng, Z. (2011). A Common HLA–DPA1 Variant is a Major Determinant of Hepatitis B Virus Clearance in Han Chinese. *The Journal of Infectious Diseases* 203, 943-947. doi:10.1093/infdis/jiq154.

Bae, J. S., Kim, J., Pasaje, C. F. A., Cheong, H. S., Lee, T. H., Koh, I. S. et al. (2012). Association study of genetic variations in microRNAs with the risk of hepatitis B-related liver diseases. *Digestive and Liver Disease* 44, 849-854. doi:10.1016/j.dld.2012.04.021.

Behelgardi, A., Hosseini, S. M., Mohebbi, S. R., Azimzadeh, P., Derakhshani, S., Karimi, K. et al. (2015). A Study on Genetic Association of Interleukin-16 Single Nucleotide Polymorphism (rs1131445) With Chronic Hepatitis B Virus Infection in Iranian Patients. *Jundishapur Journal of Microbiology* 8, doi:10.5812/jjm.23411.

Biswas, A., Panigrahi, R., Pal, M., De, B. K., Chakrabarti, S., Ghosh, M. K. et al. (2013). Association of Interleukin-1β and Gene Polymorphisms with Liver Pathogenesis in Hepatitis B Virus Infection among Eastern Indian Population. *Journal of Clinical and Experimental Hepatology* 3, 281-287. doi:10.1016/j.jceh.2013.11.006.

Cai, W., Yin, L., Wang, S., Wei, Y., Cao, W., Cheng, J. (2015). Correlation between polymorphisms of the E-selectin gene, hepatitis B virus DNA copies, pre-S1 antigen and clinical outcomes during chronic hepatitis B. *International journal of clinical and experimental medicine* 8, 2893.

CAI, W. J., YIN, L., ZHOU, D. I., CAO, W. J., ZHENG, W. W., SHENG, L. et al. (2012). Association between polymorphisms of the E-selectin gene, hepatitis B virus DNA copies and preS1 antigen in patients with chronic hepatitis B infection. *Molecular Medicine Reports* 6, 1069-1074. doi:10.3892/mmr.2012.1035.

Cao, B., Liu, X., Hou, F., Li, W., Han, Z., Zhang, Q. et al. (2009). The haplotype of the MxA gene promoter is associated with hepatitis B virus infection in a Chinese population. *Liver Int* 29, 1383-8. doi:10.1111/j.1478-3231.2009.02053.x.

Cao, L., Zhang, T., Zhu, J., Li, A., Zheng, K., Zhang, N. et al. (2017). Polymorphism of TLR5 rs5744174 is associated with disease progression in Chinese patients with chronic HBV infection. *APMIS* 125, 708-716. doi:10.1111/apm.12707.

Chang, S. W., Fann, C. S., Su, W. H., Wang, Y. C., Weng, C. C., Yu, C. J. et al. (2014). A genome-wide association study on chronic HBV infection and its clinical progression in male Han-Taiwanese. *PLoS One* 9, e99724. doi:10.1371/journal.pone.0099724.

Chen, D., Zeng, Y., Zhou, J., Yang, L., Jiang, S., Huang, J. et al. (2010). Association of candidate susceptible loci with chronic infection with hepatitis B virus in a Chinese population. *Journal of Medical Virology* 82, 371-378. doi:10.1002/jmv.21716.

CHEN, M., CHANG, Y., TANG, F., XIE, Q., LI, J., YANG, H. et al. (2014). Influence of cytotoxic T lymphocyte-associated antigen 4 polymorphisms on the outcomes of hepatitis B virus infection. *Molecular Medicine Reports* 9, 645-652. doi:10.3892/mmr.2013.1825.

Cheong, H. S., Lee, J., Yu, S. J., Yoon, J., Lee, H., Cheong, J. Y. et al. (2015). Association ofVARS2-SFTA2 polymorphisms with the risk of chronic hepatitis B in a Korean population. *Liver International* 35, 1934-1940. doi:10.1111/liv.12740.

Cheong, J. Y., Shin, H. D., Kim, Y. J. and Cho, S. W. (2013). Association of polymorphism in MicroRNA 219-1 with clearance of hepatitis B virus infection. *Journal of Medical Virology* 85, 808-814. doi:10.1002/jmv.23551.

Cheong, J. Y., Shin, H. D., Cho, S. W. and Kim, Y. J. (2014). Association of Polymorphism in MicroRNA 604 with Susceptibility to Persistent Hepatitis B Virus Infection and Development of Hepatocellular Carcinoma. *Journal of Korean Medical Science* 29, 1523. doi:10.3346/jkms.2014.29.11.1523.

Cheong, J. Y., Cho, S. W., Choi, J. Y., Lee, J. A., Kim, M. H., Lee, J. E. et al. (2007). RANTES, MCP-1, CCR2, CCR5, CXCR1 and CXCR4 Gene Polymorphisms are not Associated with the Outcome of Hepatitis B Virus Infection: Results from a Large Scale Single Ethnic Population. *Journal of Korean Medical Science* 22, 529. doi:10.3346/jkms.2007.22.3.529.

Cheong, J. Y., Cho, S. W., Hwang, I. L., Yoon, S. K., Lee, J. H., Park, C. S. et al. (2006). Association between chronic hepatitis B virus infection and interleukin-10, tumor necrosis factor-alpha gene promoter polymorphisms. *Journal of Gastroenterology and Hepatology* 21, 1163-1169. doi:10.1111/j.1440-1746.2006.04304.x.

Cheong, J. Y., Cho, S. W., Chung, S. G., Lee, J. A., Yeo, M., Wang, H. J. et al. (2006). Genetic Polymorphism of Interferon-γ, Interferon-γ Receptor, and Interferon Regulatory Factor-1 Genes in Patients with Hepatitis B Virus Infection. *Biochemical Genetics* 44, 246-255. doi:10.1007/s10528-006-9029-y.

Chong, W. P., To, Y. F., Ip, W. K., Yuen, M. F., Poon, T. P., Wong, W. H. S. et al. (2005). Mannose-binding lectin in chronic hepatitis B virus infection. *Hepatology* 42, 1037-1045. doi:10.1002/hep.20891.

Correa, B. M., Lopes, E. P., Albuquerque, M. F. and Dourado, L. (2012). Association between HLA-DRB1* polymorphisms and hepatitis B infection in a brazilian population. *Rev Assoc Med Bras (1992)* 58, 537-42.

Cui, W., Sun, C., Deng, B. and Liu, P. (2013). Association of polymorphisms in the interleukin-4 gene with response to hepatitis B vaccine and susceptibility to hepatitis B virus infection: A meta-analysis. *Gene* 525, 35-40. doi:10.1016/j.gene.2013.04.065.

Deng, G., Zhou, G., Zhai, Y., Li, S., Li, X., Li, Y. et al. (2004). Association of estrogen receptor ? polymorphisms with susceptibility to chronic hepatitis B virus infection. *Hepatology* 40, 318-326. doi:10.1002/hep.20318.

Deng, Y., Xie, M., Xie, L., Wang, J., Li, T., He, Y. et al. (2015). Association between Polymorphism of the Interleukin-13 Gene and Susceptibility to Hepatocellular Carcinoma in the Chinese Population. *PLOS ONE* 10, e0116682. doi:10.1371/journal.pone.0116682.

Doganay, L. (2014). Association of human leukocyte antigen DQB1 and DRB1 alleles with chronic hepatitis B. *World Journal of Gastroenterology* 20, 8179. doi:10.3748/wjg.v20.i25.8179.

Du, T., Guo, X. H., Zhu, X. L., Li, J. H., Lu, L. P., Gao, J. R. et al. (2006). Association of TNF-alpha promoter polymorphisms with the outcomes of hepatitis B virus infection in Chinese Han population. *Journal of Viral Hepatitis* 13, 618-624. doi:10.1111/j.1365-2893.2006.00731.x.

Du, Y., Han, X., Pu, R., Xie, J., Zhang, Y., Cao, G. (2014). Association of miRNA-122-binding site polymorphism at the interleukin-1 α gene and its interaction with hepatitis B virus mutations with hepatocellular carcinoma risk. *Frontiers of Medicine* 8, 217-226. doi:10.1007/s11684-014-0326-2.

Duan, S., Zhang, G., Han, Q., Li, Z., Liu, Z., Chen, J. et al. (2011). CTLA-4 exon 1 +49 polymorphism alone and in a haplotype with −318 promoter polymorphism may confer susceptibility to chronic HBV infection in Chinese Han patients. *Molecular Biology Reports* 38, 5125-5132. doi:10.1007/s11033-010-0660-7.

Eskandari, E., Dahmardeh, T., Dahmardeh, F., Pahlevani, E. and Metanat, M. (2017). Lack of relationship between PTEN 32-bp and TP53 16-bp Ins/Del polymorphisms and chronic hepatitis B virus infection. *VirusDisease* 28, 289-294. doi:10.1007/s13337-017-0391-7.

Eskandari, E., Metanat, M., Pahlevani, E. and Nakhzari-Khodakheir, T. (2017). Association between TGFβ1 polymorphisms and chronic hepatitis B infection in an Iranian population. *Revista da Sociedade Brasileira de Medicina Tropical* 50, 301-308. doi:10.1590/0037-8682-0266-2016.

Eskandari, E., Dahmardeh, T., Safdari, V., Khosravi, S. and Pahlevani, E. (2017). HLA-G gene 14-bp deletion variant protects Iranian subjects against chronic hepatitis B infection. *International Journal of Immunogenetics* 44, 322-327. doi:10.1111/iji.12337.

Ezzikouri, S., Chihab, H., Elhabazi, A., Wakrim, L. and Benjelloun, S. (2017). Lack of Ser267Phe variant of sodium taurocholate cotransporting polypeptide among Moroccans regardless of hepatitis B virus infection status. *BMC Infectious Diseases* 17, doi:10.1186/s12879-017-2214-2.

Ezzikouri, S., Kitab, B., Rebbani, K., Marchio, A., Wain-Hobson, S., Dejean, A. et al. (2013). Polymorphic APOBEC3 modulates chronic hepatitis B in Moroccan population. *Journal of Viral Hepatitis* 20, 678-686. doi:10.1111/jvh.12042.

Fan, J., Hou, S., Qing-Ling, L., Hu, J., Peng, H., Guo, J. (2016). Association of HLA-DQ and IFNL4 polymorphisms with susceptibility to hepatitis B virus infection and clearance. *Annals of hepatology* 15, 532. doi:10.5604/16652681.1202946.

Fan, J., Huang, X., Chen, J., Cai, Y., Xiong, L., Mu, L. et al. (2016). Host Genetic Variants in HLA Loci Influence Risk for Hepatitis B Virus Infection in Children. *Hepatitis Monthly* 16, doi:10.5812/hepatmon.37786.

Fan, J., Cai, Y., Huang, X., Wang, Y., Mu, L., Zhou, L. (2017). Variations in IL-1R1 Gene Influence Risk for Hepatitis B Virus Infection of Children in a Han Chinese population. *International Journal of Infectious Diseases* 55, 45-50. doi:10.1016/j.ijid.2016.12.021.

Ferreira, S. D. C., Chachá, S. G. F., Souza, F. F., Teixeira, A. C., Santana, R. D. C., Deghaide, N. H. S. et al. (2017). The HLA-G 14-base pair deletion allele and the deletion/deletion genotype are associated with persistent HBe antigenemia in chronic hepatis B infection. *Human Immunology* 78, 166-171. doi:10.1016/j.humimm.2016.12.011.

Filho, R. M., Carmo, R. F., Catsman, C., Souza, C., Silva, A., Moura, P. et al. (2010). High Frequency of Variant Alleles of the Mannose-Binding Lectin 2 (MBL2) Gene Are Associated with Patients Infected by Hepatitis B Virus. *Viral Immunology* 23, 449-453. doi:10.1089/vim.2009.0105.

Fletcher, G. J., Samuel, P., Christdas, J., Gnanamony, M., Ismail, A. M., Anantharam, R. et al. (2011). Association of HLA and TNF polymorphisms with the outcome of HBV infection in the South Indian population. *Genes & Immunity* 12, 552-558. doi:10.1038/gene.2011.32.

Fletcher, G. J., Gnanamony, M., Samuel, P., Ismail, A. M., Kannangai, R., Daniel, D. et al. (2010). Association of mannose-binding lectin polymorphisms and HBV outcome in a South Indian population. *International Journal of Immunogenetics* 37, 177-184. doi:10.1111/j.1744-313X.2010.00908.x.

Gao, F., Zhang, Y., Wang, L., Wei, Y., Wang, J., Wang, C. et al. (2015). A Meta-Analysis of the Correlation Between the HLA-DRB1*03 Allele and Chronic Hepatitis B in the Han Chinese Population. *Genetic Testing and Molecular Biomarkers* 19, 218-221. doi:10.1089/gtmb.2014.0096.

Gao, X., Jiao, Y., Wang, L., Liu, X., Sun, W., Cui, B. et al. (2010). Inhibitory KIR and specific HLA-C gene combinations confer susceptibility to or protection against chronic hepatitis B. *Clinical Immunology* 137, 139-146. doi:10.1016/j.clim.2010.05.011.

Geng, P., Song, L., An, H., Huang, J., Li, S., Zeng, X. (2016). Toll-Like Receptor 3 is Associated With the Risk of HCV Infection and HBV-Related Diseases. *Medicine* 95, e2302. doi:10.1097/MD.0000000000002302.

Guo, X., Zhang, Y., Li, J., Ma, J., Wei, Z., Tan, W. et al. (2011). Strong influence of human leukocyte antigen (HLA)-DP gene variants on development of persistent chronic hepatitis B virus carriers in the Han Chinese population. *Hepatology* 53, 422-428. doi:10.1002/hep.24048.

Han, Q., Yang, C., Li, N., Li, F., Sang, J., Lv, Y. et al. (2017). Association of genetic variation in B-cell activating factor with chronic hepatitis B virus infection. *Immunology Letters* 188, 53-58. doi:10.1016/j.imlet.2017.06.005.

He, D., Tao, S., Guo, S., Li, M., Wu, J., Huang, H. et al. (2015). Interaction of TLR-IFN and HLA polymorphisms on susceptibility of chronic HBV infection in Southwest Han Chinese. *Liver International* 35, 1941-1949. doi:10.1111/liv.12756.

He, Q., Huang, Y., Zhang, L., Yan, Y., Liu, J., Song, X. et al. (2018). Association between vitamin D receptor polymorphisms and hepatitis B virus infection susceptibility: A meta-analysis study. *Gene* 645, 105-112. doi:10.1016/j.gene.2017.12.027.

Hoan, N. X., Van Tong, H., Giang, D. P., Cuong, B. K., Toan, N. L., Wedemeyer, H. et al. (2017). *SOCS3* genetic variants and promoter hypermethylation in patients with chronic hepatitis B. *Oncotarget* 8, 17127-17139. doi:10.18632/oncotarget.15083.

Hou, S., Hu, J., Zhang, Y., Li, Q. and Guo, J. (2015). Effects of interaction between genetic variants in human leukocyte antigen DQ and granulysin genes in Chinese Han subjects infected with hepatitis B virus. *Microbiology and Immunology* 59, 209-218. doi:10.1111/1348-0421.12239.

Hu, H., Liu, J., Lin, Y., Luo, W., Chu, Y., Chang, C. et al. (2016). The rs2296651 (S267F) variant on NTCP (SLC10A1) is inversely associated with chronic hepatitis B and progression to cirrhosis and hepatocellular carcinoma in patients with chronic hepatitis B. *Gut* 65, 1514-1521. doi:10.1136/gutjnl-2015-310686.

Hu, L., Zhai, X., Liu, J., Chu, M., Pan, S., Jiang, J. et al. (2012). Genetic variants in human leukocyte antigen/DP-DQ influence both hepatitis B virus clearance and hepatocellular carcinoma development. *Hepatology* 55, 1426-1431. doi:10.1002/hep.24799.

Hu, Z., Yang, J., Xiong, G., Shi, H., Yuan, Y., Fan, L. et al. (2014). HLA-DPB1 Variant Effect on Hepatitis B Virus Clearance and Liver Cirrhosis Development Among Southwest Chinese Population. *Hepatitis Monthly* 14, doi:10.5812/hepatmon.19747.

Hu, Z., Liu, Y., Zhai, X., Dai, J., Jin, G., Wang, L. et al. (2013). New loci associated with chronic hepatitis B virus infection in Han Chinese. *Nature Genetics* 45, 1499-1503. doi:10.1038/ng.2809.

HUANG, J., XIONG, L., WANG, J., LIU, Y., ZHU, Q., LEI, J. et al. (2016). Association between the HLA-DQB1 polymorphisms and the susceptibility of chronic hepatitis B: A comprehensive meta-analysis. *Biomedical Reports* 4, 557-566. doi:10.3892/br.2016.632.

Huang, R., Hao, Y., Fan, Y., Yang, C., Wu, K., Cao, S. et al. (2013). Association Between Cytotoxic T-Lymphocyte-Associated Antigen 4 +49A/G Polymorphism and Persistent Hepatitis B Virus Infection in the Asian Population: Evidence from the Current Studies. *Genetic Testing and Molecular Biomarkers* 17, 601-606. doi:10.1089/gtmb.2013.0069.

Huang, X., Li, H., Wang, J., Huang, C., Lu, Y., Qin, X. et al. (2015). Genetic polymorphisms in Toll-like receptor 3 gene are associated with the risk of hepatitis B virus-related liver diseases in a Chinese population. *Gene* 569, 218-224. doi:10.1016/j.gene.2015.05.054.

Ji, X., Zhang, Q., Li, B., Du, Y., Yin, J., Liu, W. et al. (2014). Impacts of human leukocyte antigen DQ genetic polymorphisms and their interactions with hepatitis B virus mutations on the risks of viral persistence, liver cirrhosis, and hepatocellular carcinoma. *Infection, Genetics and Evolution* 28, 201-209. doi:10.1016/j.meegid.2014.09.032.

Jiang, D., Ma, X., Yu, H., Cao, G., Ding, D., Chen, H. et al. (2015). Genetic variants in five novel loci includingCFB andCD40 predispose to chronic hepatitis B. *Hepatology* 62, 118-128. doi:10.1002/hep.27794.

JIANG, H., CAO, H., LIU, G., HUANG, Q. and LI, Y. (2014). IL-18 promoter -137G/C polymorphism correlates with chronic hepatitis B and affects the expression of interleukins. *Acta virologica* 58, 28-33. doi:10.4149/av_2014_01_28.

Jiang, X., Su, K., Tao, J., Fan, R., Xu, Y., Han, H. et al. (2016). Association of STAT4 polymorphisms with hepatitis B virus infection and clearance in Chinese Han population. *Amino Acids* 48, 2589-2598. doi:10.1007/s00726-016-2283-3.

Jung, Y. J., Kim, Y. J., Kim, L. H., Lee, S. O., Park, B. L., Shin, H. D. et al. (2007). Putative Association of *Fas* and *FasL* Gene Polymorphisms with Clinical Outcomes of Hepatitis B Virus Infection. *Intervirology* 50, 369-376. doi:10.1159/000109751.

Kamatani, Y., Wattanapokayakit, S., Ochi, H., Kawaguchi, T., Takahashi, A., Hosono, N. et al. (2009). A genome-wide association study identifies variants in the HLA-DP locus associated with chronic hepatitis B in Asians. *Nature Genetics* 41, 591-595. doi:10.1038/ng.348.

Karamitros, T., Papatheodoridis, G., Dimopoulou, E., Papageorgiou, M., Paraskevis, D., Magiorkinis, G. et al. (2015). The interferon receptor-1 promoter polymorphisms affect the outcome of Caucasians with HBeAg-negative chronic HBV infection. *Liver International* 35, 2506-2513. doi:10.1111/liv.12859.

Karataylı, S. C., Bozdayı, M., Karataylı, E., Öztürk, T., Husseini, A. A., Albayrak, R. et al. (2015). Interleukin-28 gene polymorphisms may contribute to HBsAg persistence and the development of HBeAg-negative chronic hepatitis B. *Liver International* 35, 846-853. doi:10.1111/liv.12595.

Karra, V. K., Gumma, P. K., Chowdhury, S. J., Ruttala, R., Polipalli, S. K., Chakravarti, A. et al. (2015). IL-18 polymorphisms in hepatitis B virus related liver disease. *Cytokine* 73, 277-282. doi:10.1016/j.cyto.2015.02.015.

Khanizadeh, S., Ravanshad, M., Mohebbi, S. R., Naghoosi, H., Tahaei, M. E., Mousavi Nasab, S. D. et al. (2012). Polymorphisms Within the Promoter Region of the Gamma Interferon (IFN-γ) Receptor1 Gene are Associated With the Susceptibility to Chronic HBV Infection in an Iranian Population. *Hepatitis Monthly* 12, doi:10.5812/hepatmon.7283.

Khorramdelazad, H., Hakimizadeh, E., Hassanshahi, G., Rezayati, M., Sendi, H., Arababadi, M. K. (2013). CCR5 Δ 32 mutation is not prevalent in Iranians with chronic HBV infection. *Journal of Medical Virology* 85, 964-968. doi:10.1002/jmv.23510.

Kim, H. Y., Yoon, J., Lee, H., Cheong, J. Y., Cho, S. W., Shin, H. D. et al. (2014). MicroRNA-196A-2 polymorphisms and hepatocellular carcinoma in patients with chronic hepatitis B. *Journal of Medical Virology* 86, 446-453. doi:10.1002/jmv.23848.

Kim, J., Yu, S. J., Park, B., Cheong, H. S., Pasaje, C. F. A., Bae, J. S. et al. (2011). *TGFBR3* Polymorphisms and Its Haplotypes Associated with Chronic Hepatitis B Virus Infection and Age of Hepatocellular Carcinoma Occurrence. *Digestive Diseases* 29, 278-283. doi:10.1159/000327559.

Kim, L. H., Cheong, H. S., Namgoong, S., Kim, J. O., Kim, J., Park, B. L. et al. (2015). Replication of genome wide association studies on hepatocellular carcinoma susceptibility loci of STAT4 and HLA-DQ in a Korean population. *Infection, Genetics and Evolution* 33, 72-76. doi:10.1016/j.meegid.2015.04.013.

Kim, Y. J. (2003). Association of TNF-  promoter polymorphisms with the clearance of hepatitis B virus infection. *Human Molecular Genetics* 12, 2541-2546. doi:10.1093/hmg/ddg262.

Kim, Y. J., Kim, H. Y., Kim, J. S., Lee, J. H., Yoon, J. H., Kim, C. Y. et al. (2009). Putative association of transforming growth factor-α polymorphisms with clearance of hepatitis B virus and occurrence of hepatocellular carcinoma in patients with chronic hepatitis B virus infection. *Journal of Viral Hepatitis* doi:10.1111/j.1365-2893.2009.01205.x.

Kim, Y. J., Kim, H. Y., Lee, J., Yu, S. J., Yoon, J., Lee, H. et al. (2013). A genome-wide association study identified new variants associated with the risk of chronic hepatitis B. *Human Molecular Genetics* 22, 4233-4238. doi:10.1093/hmg/ddt266.

Kimkong, I., Tangkijvanich, P. and Hirankarn, N. (2013). Association of interferon-alpha gene polymorphisms with chronic hepatitis B virus infection. *International Journal of Immunogenetics* 40, 476-481. doi:10.1111/iji.12055.

Kummee, P., Tangkijvanich, P., Poovorawan, Y. and Hirankarn, N. (2007). Association of HLA-DRB1*13 and TNF-? gene polymorphisms with clearance of chronic hepatitis B infection and risk of hepatocellular carcinoma in Thai population. *Journal of Viral Hepatitis* 070727121914001-??? doi:10.1111/j.1365-2893.2007.00880.x.

Laaribi, A. B., Hannachi, N., Ben Yahia, H., Marzouk, M., Mehri, A., Belhadj, M. et al. (2018). Human leukocyte antigen (HLA-F) polymorphism is associated with chronic HBV infection. *3 Biotech* 8, doi:10.1007/s13205-017-1079-9.

Laaribi, A. B., Zidi, I., Hannachi, N., Ben Yahia, H., Chaouch, H., Bortolotti, D. et al. (2015). Association of an HLA-G 14-bp Insertion/Deletion polymorphism with high HBV replication in chronic hepatitis. *Journal of Viral Hepatitis* 22, 835-841. doi:10.1111/jvh.12395.

Lau, K., Lam, C., Law, C., Lai, S., Tsang, T., Siu, C. W. et al. (2011). Non-invasive screening of HLA-DPA1 and HLA-DPB1 alleles for persistent hepatitis B virus infection: Susceptibility for vertical transmission and toward a personalized approach for vaccination and treatment. *Clinica Chimica Acta* 412, 952-957. doi:10.1016/j.cca.2011.01.030.

Lee, D. H., Lee, J. H., Kim, Y. J., Park, N. H., Cho, Y., Lee, Y. B. et al. (2014). Relationship between polymorphisms near theIL28B gene and spontaneous HBsAg seroclearance: a systematic review and meta-analysis. *Journal of Viral Hepatitis* 21, 163-170. doi:10.1111/jvh.12193.

Li, F., Li, X., Zou, G., Gao, Y. and Ye, J. (2017). Association betweenTLR7 copy number variations and hepatitis B virus infection outcome in Chinese. *World Journal of Gastroenterology* 23, 1602. doi:10.3748/wjg.v23.i9.1602.

Li, J., Yang, D., He, Y., Wang, M., Wen, Z., Liu, L. et al. (2011). Associations of HLA-DP Variants with Hepatitis B Virus Infection in Southern and Northern Han Chinese Populations: A Multicenter Case-Control Study. *PLoS ONE* 6, e24221. doi:10.1371/journal.pone.0024221.

Li, M., Li, F., Li, N., Sang, J., Fan, X., Deng, H. et al. (2018). Association of polymorphism rs1053005 in STAT3 with chronic hepatitis B virus infection in Han Chinese population. *BMC Medical Genetics* 19, doi:10.1186/s12881-018-0569-x.

Li, N. (2012). Relationship between interleukin 18 polymorphisms and susceptibility to chronic hepatitis B virus infection. *World Journal of Hepatology* 4, 105. doi:10.4254/wjh.v4.i3.105.

Li, N., Zhu, Q., Li, Z., Han, Q., Zhang, G., Chen, J. et al. (2014). IL17A gene polymorphisms, serum IL-17A and IgE levels, and hepatocellular carcinoma risk in patients with chronic hepatitis B virus infection. *Molecular Carcinogenesis* 53, 447-457. doi:10.1002/mc.21992.

Li, N., Zhang, P., Yang, C., Zhu, Q., Li, Z., Li, F. et al. (2014). Association of Genetic Variation of Sodium Taurocholate Cotransporting Polypeptide with Chronic Hepatitis B Virus Infection. *Genetic Testing and Molecular Biomarkers* 18, 425-429. doi:10.1089/gtmb.2013.0491.

Li, N., Zhu, Q., Li, Z., Han, Q., Chen, J., Lv, Y. et al. (2013). IL21 and IL21R polymorphisms and their interactive effects on serum IL-21 and IgE levels in patients with chronic hepatitis B virus infection. *Human Immunology* 74, 567-573. doi:10.1016/j.humimm.2013.01.005.

Li, S., Deng, Y., Chen, Z., Huang, S., Liao, X., Lin, L. et al. (2011). Genetic polymorphism of interleukin-16 influences susceptibility to HBV-related hepatocellular carcinoma in a Chinese population. *Infection, Genetics and Evolution* 11, 2083-2088. doi:10.1016/j.meegid.2011.09.025.

Li, W., Jiang, Y., Jin, Q., Shi, X., Jin, J., Gao, Y. et al. (2011). Expression and gene polymorphisms of interleukin 28B and hepatitis B virus infection in a Chinese Han population. *Liver International* 31, 1118-1126. doi:10.1111/j.1478-3231.2011.02507.x.

Li, X., Liu, W., Wang, H., Jin, X., Fang, S., Shi, Y. et al. (2012). The influence of HLA alleles and HBV subgenotyes on the outcomes of HBV infections in Northeast China. *Virus Research* 163, 328-333. doi:10.1016/j.virusres.2011.10.020.

Li, Y., Si, L., Zhai, Y., Hu, Y., Hu, Z., Bei, J. et al. (2016). Genome-wide association study identifies 8p21.3 associated with persistent hepatitis B virus infection among Chinese. *Nature Communications* 7, 11664. doi:10.1038/ncomms11664.

Liao, Y., Cai, B., Li, Y., Chen, J., Tao, C., Huang, H. et al. (2014). Association of HLA-DP/DQ and STAT4 Polymorphisms with HBV Infection Outcomes and a Mini Meta-Analysis. *PLoS ONE* 9, e111677. doi:10.1371/journal.pone.0111677.

Liao, Y., Cai, B., Li, Y., Chen, J., Ying, B., Tao, C. et al. (2015). Association of HLA-DP/DQ, STAT4 and IL-28B variants with HBV viral clearance in Tibetans and Uygurs in China. *Liver International* 35, 886-896. doi:10.1111/liv.12643.

Liu, M., Cao, B., Zhang, H., Dai, Y., Liu, X., Xu, C. (2006). Association of interferon-gamma gene haplotype in the Chinese population with hepatitis B virus infection. *Immunogenetics* 58, 859-864. doi:10.1007/s00251-006-0161-y.

Liu, Y., Song, C., Ni, H., Jiao, W., Gan, W., Dong, X. et al. (2018). UBE2L3, a susceptibility gene that plays oncogenic role in hepatitis B-related hepatocellular carcinoma. *Journal of Viral Hepatitis* doi:10.1111/jvh.12963.

Liu, Y., Xie, K., Wen, J., Deng, M., Li, J., Hu, Z. (2014). A genetic variant in microRNA-122 regulatory region confers risk for chronic hepatitis B virus infection and hepatocellular carcinoma in Han Chinese. *Journal of Medical Virology* 86, 1669-1674. doi:10.1002/jmv.23996.

Liu, Y., Zhang, Y., Wen, J., Liu, L., Zhai, X., Liu, J. et al. (2012). A Genetic Variant in the Promoter Region of miR-106b-25 Cluster and Risk of HBV Infection and Hepatocellular Carcinoma. *PLoS ONE* 7, e32230. doi:10.1371/journal.pone.0032230.

Liu, Y., Liu, Y., Huang, X., Sui, J., Mo, C., Wang, J. et al. (2014). Association of PvuII and XbaI polymorphisms in estrogen receptor alpha gene with the risk of hepatitis B virus infection in the Guangxi Zhuang population. *Infection, Genetics and Evolution* 27, 69-76. doi:10.1016/j.meegid.2014.07.002.

Lu, Y., Peng, J., Wang, C., Zhu, Y., Wang, F., Sun, Z.. (2014). IL-6 promoter functional polymorphism -572C/G affects spontaneous clearance of hepatitis B virus infection. *Clin Lab.* 11, 1903-7.

Lu, Y., Bao, J., Deng, Y., Rong, C., Liu, Y., Huang, X. et al. (2015). Role of IL-18 Gene Promoter Polymorphisms, Serum IL-18 Levels, and Risk of Hepatitis B Virus-related Liver Disease in the Guangxi Zhuang Population: a Retrospective Case-Control Study. *Asian Pacific Journal of Cancer Prevention* 16, 6019-6026. doi:10.7314/APJCP.2015.16.14.6019.

Lu, Y., Zhu, Y., Peng, J., Wang, X., Wang, F., Sun, Z. (2015). STAT4 genetic polymorphisms association with spontaneous clearance of hepatitis B virus infection. *Immunologic Research* 62, 146-152. doi:10.1007/s12026-015-8645-1.

Lu, Y., Wu, Z., Peng, Q., Ma, L., Zhang, X., Zhao, J. et al. (2014). Role of IL-4 gene polymorphisms in HBV-related hepatocellular carcinoma in a Chinese population. *PLoS One* 9, e110061. doi:10.1371/journal.pone.0110061.

Lu, Z., Zhang, B., Chen, S., Gai, Z., Feng, Z., Liu, X. et al. (2008). Association of KIR Genotypes and Haplotypes with Susceptibility to Chronic Hepatitis B Virus Infection in Chinese Han Population. *Cellular & Molecular Immunology* 5, 457-463. doi:10.1038/cmi.2008.57.

Ma, N., Zhang, X., Yang, L., Zhou, J., Liu, W., Gao, X. et al. (2018). Role of FunctionalIFNL4 ,IFNLR1 ,IFNA, IFNAR2 Polymorphisms in Hepatitis B virus-related liver disease in Han Chinese population. *Journal of Viral Hepatitis* 25, 306-313. doi:10.1111/jvh.12817.

Ma, N., Zhang, X., Yu, F., Gao, P., Fan, Q., Liu, L. et al. (2014). Role ofIFN-λs ,IFN-λs related genes and theDEPDC5 gene in Hepatitis B virus-related liver disease. *Journal of Viral Hepatitis* 21, e29-e38. doi:10.1111/jvh.12235.

Mbarek, H., Ochi, H., Urabe, Y., Kumar, V., Kubo, M., Hosono, N. et al. (2011). A genome-wide association study of chronic hepatitis B identified novel risk locus in a Japanese population. *Human Molecular Genetics* 20, 3884-3892. doi:10.1093/hmg/ddr301.

Miao, F., Sun, H., Pan, N., Xu, J., Qiu, J., Shen, Y. et al. (2013). Association of Human Leukocyte Antigen Class I Polymorphism with Spontaneous Clearance of Hepatitis B Surface Antigen in Qidong Han Population. *Clinical and Developmental Immunology* 2013, 1-7. doi:10.1155/2013/145725.

Mohammadi, A., Tajik, N., Shah-Hosseini, A., Alavian, S. M., Sharifi, Z., Jarahi, L. (2015). FAS and FAS-Ligand Promoter Polymorphisms in Hepatitis B Virus Infection. *Hepatitis Monthly* 15, doi:10.5812/hepatmon.26490.

Moudi, B., Heidari, Z., Mahmoudzadeh-Sagheb, H. and Hashemi, M. (2016). Gene polymorphisms of macrophage migration inhibitory factor affect susceptibility to chronic hepatitis B virus infection in an Iranian cohort. *Microbiology and Immunology* 60, 390-396. doi:10.1111/1348-0421.12382.

Namgoong, S., Shin, J., Cheong, H. S., Kim, L. H., Kim, J. O., Seo, J. Y. et al. (2018). Genetic association of complement component 2 variants with chronic hepatitis B in a Korean population. *Liver International* 38, 1576-1582. doi:10.1111/liv.13675.

Nfor, O. N., Wu, M. F., Debnath, T., Lee, C. T., Lee, W., Liu, W. H. et al. (2018). Hepatitis B virus infection in Taiwan: The role of NTCP rs2296651 variant in relation to sex. *Journal of Viral Hepatitis* doi:10.1111/jvh.12912.

Nishida, N., Ohashi, J., Sugiyama, M., Tsuchiura, T., Yamamoto, K., Hino, K. et al. (2015). Effects of HLA-DPB1 genotypes on chronic hepatitis B infection in Japanese individuals. *Tissue Antigens* 86, 406-412. doi:10.1111/tan.12684.

Nishida, N., Sawai, H., Matsuura, K., Sugiyama, M., Ahn, S. H., Park, J. Y. et al. (2012). Genome-Wide Association Study Confirming Association of HLA-DP with Protection against Chronic Hepatitis B and Viral Clearance in Japanese and Korean. *PLoS ONE* 7, e39175. doi:10.1371/journal.pone.0039175.

Nishida, N., Sawai, H., Kashiwase, K., Minami, M., Sugiyama, M., Seto, W. et al. (2014). New Susceptibility and Resistance HLA-DP Alleles to HBV-Related Diseases Identified by a Trans-Ethnic Association Study in Asia. *PLoS ONE* 9, e86449. doi:10.1371/journal.pone.0086449.

Park, B. L., Kim, Y. J., Cheong, H. S., Kim, L. H., Choi, Y. H., Lee, H. et al. (2006). Association of common promoter polymorphisms of MCP1 with hepatitis B virus clearance. *Experimental & Molecular Medicine* 38, 694-702. doi:10.1038/emm.2006.82.

Peng, L., Guo, J., Zhang, Z., Liu, L., Cao, Y., Shi, H. et al. (2013). A Candidate Gene Study for the Association of Host Single Nucleotide Polymorphisms with Liver Cirrhosis Risk in Chinese Hepatitis B Patients. *Genetic Testing and Molecular Biomarkers* 17, 681-686. doi:10.1089/gtmb.2013.0058.

Peng, L., Zhao, Q., Li, Q., Li, M., Li, C., Xu, T. et al. (2015). The p.Ser267Phe variant inSLC10A1 is associated with resistance to chronic hepatitis B. *Hepatology* 61, 1251-1260. doi:10.1002/hep.27608.

Peng, Q., Qin, X., He, Y., Chen, Z., Deng, Y., Li, T. et al. (2013). Association of IL27 gene polymorphisms and HBV-related hepatocellular carcinoma risk in a Chinese population. *Infection, Genetics and Evolution* 16, 1-4. doi:10.1016/j.meegid.2013.01.015.

Peng, X. M., Lei, R. X., Gu, L., Ma, H. H., Xie, Q. F., Gao, Z. L. (2007). Influences of MxA gene ?88 G/T and IFN-gamma +874 A/T on the natural history of hepatitis B virus infection in an endemic area. *International Journal of Immunogenetics* 34, 341-346. doi:10.1111/j.1744-313X.2007.00696.x.

Pereira, V. R. Z. B., Wolf, J. M., Stumm, G. Z., Boeira, T. R., Galvan, J., Simon, D. et al. (2017). Lack of association between human leukocyte antigen polymorphisms rs9277535 and rs7453920 and chronic hepatitis B in a Brazilian population. *Genetics and molecular research : GMR* 16,

Pires-Neto, O. D. S., de Sá, K. S. G., Santana, B. B., Tatielle Monteiro Gomes, S., Da Silva Graça Amoras, E., Da Silva Conde, S. R. et al. (2015). Lack of Association between Polymorphisms of theTLR4 Gene and Infection with the Hepatitis B and C Viruses. *Mediators of Inflammation* 2015, 1-7. doi:10.1155/2015/150673.

Posuwan, N., Payungporn, S., Tangkijvanich, P., Ogawa, S., Murakami, S., Iijima, S. et al. (2014). Genetic Association of Human Leukocyte Antigens with Chronicity or Resolution of Hepatitis B Infection in Thai Population. *PLoS ONE* 9, e86007. doi:10.1371/journal.pone.0086007.

Qi, S., Cao, B., Jiang, M., Xu, C., Dai, Y., Li, K. et al. (2005). Association of the −183 polymorphism in the IFN-γ gene promoter with hepatitis B virus infection in the Chinese population. *Journal of Clinical Laboratory Analysis* 19, 276-281. doi:10.1002/jcla.20090.

Razavi, A. H., Azimzadeh, P., Mohebbi, S. R., Hosseini, S. M., Romani, S., Khanyaghma, M. et al. (2014). Lack of Association Between Transforming Growth Factor Beta 1-509C/T and +915G/C Polymorphisms and Chronic Hepatitis B in Iranian Patients. *Hepatitis Monthly* 14, doi:10.5812/hepatmon.13100.

Rebbani, K., Ababou, M., Nadifi, S., Kandil, M., Marchio, A., Pineau, P. et al. (2017). Myxovirus resistance 1 gene polymorphisms and outcomes of viral hepatitis B and C infections in Moroccan patients. *Journal of Medical Virology* 89, 647-652. doi:10.1002/jmv.24642.

Ren, W., Wu, Z., Ma, R., Liu, Z., Wang, Y., Wu, L. et al. (2017). Polymorphisms in the IL-17 Gene (rs2275913 and rs763780) Are Associated with Hepatitis B Virus Infection in the Han Chinese Population. *Genetic Testing and Molecular Biomarkers* 21, 286-291. doi:10.1089/gtmb.2016.0177.

Romani, S., Hosseini, S. M., Mohebbi, S. R., Kazemian, S., Derakhshani, S., Khanyaghma, M. et al. (2014). Interleukin-16 Gene Polymorphisms Are Considerable Host Genetic Factors for Patients’ Susceptibility to Chronic Hepatitis B Infection. *Hepatitis Research and Treatment* 2014, 1-5. doi:10.1155/2014/790753.

Romporn, S., Hirankarn, N., Tangkijvanich, P. and Kimkong, I. (2013). Association ofIFNAR2 andIL10RB genes in chronic hepatitis B virus infection. *Tissue Antigens* 82, 21-25. doi:10.1111/tan.12133.

Santana, B. B., Viégas, M. L. C., Conde, S. R. S. S., Ishak, M. O. G., Ishak, R., Vallinoto, A. C. R. (2013). FAS andFASL Gene Polymorphisms Are Not Associated with Hepatitis B Virus Infection Based on a Case-Control Study in a Brazilian Population. *Disease Markers* 35, 741-746. doi:10.1155/2013/964145.

Saxena, R., Chawla, Y. K., Verma, I. and Kaur, J. (2014). Effect ofIL-12B ,IL-2 ,TGF-β1 , andIL-4 Polymorphism and Expression on Hepatitis B Progression. *Journal of Interferon & Cytokine Research* 34, 117-128. doi:10.1089/jir.2013.0043.

Schott, E., Witt, H., Pascu, M., van Boemmel, F., Weich, V., Bergk, A. et al. (2007). Association of CTLA4 single nucleotide polymorphisms with viral but not autoimmune liver disease. *European Journal of Gastroenterology & Hepatology* 19, 947-951. doi:10.1097/MEG.0b013e3282efa240.

Seshasubramanian, V., Soundararajan, G. and Ramasamy, P. (2017). Human leukocyte antigen A, B and Hepatitis B infection outcome: A meta-analysis. *Infect Genet Evol* doi:10.1016/j.meegid.2017.07.027.

Seto, W. K., Wong, D. K., Kopaniszen, M., Proitsi, P., Sham, P. C., Hung, I. F. et al. (2013). HLA-DP and IL28B polymorphisms: influence of host genome on hepatitis B surface antigen seroclearance in chronic hepatitis B. *Clin Infect Dis* 56, 1695-703. doi:10.1093/cid/cit121.

Shang, M., Huang, Y., Hu, X., Wang, J., Song, X., Zhou, Y. et al. (2014). Association between SNPs in miRNA-machinery genes and chronic hepatitis B in the Chinese Han population. *Infection, Genetics and Evolution* 28, 113-117. doi:10.1016/j.meegid.2014.09.015.

Shi, K. Q., Cai, X. H., Xiao, D. D., Wu, S. J., Peng, M. M., Lin, X. F. et al. (2012). Tumour necrosis factor-α-857T allele reduces the risk of hepatitis B virus infection in an Asian population. *Journal of Viral Hepatitis* 19, e66-e72. doi:10.1111/j.1365-2893.2011.01540.x.

Shin, H. D., Park, B. L., Cheong, H. S., Yoon, J. H., Kim, Y. J., Lee, H. S. (2007). SPP1 polymorphisms associated with HBV clearance and HCC occurrence. *International Journal of Epidemiology* 36, 1001-1008. doi:10.1093/ije/dym093.

Shin, J., Cheong, H. S., Kim, J. Y., Lee, J., Yu, S. J., Yoon, J. et al. (2017). Identification of additional EHMT2 variant associated with the risk of chronic hepatitis B by GWAS follow-up study. *Genes & Immunity* doi:10.1038/s41435-017-0004-x.

Shin, J., Cheong, H. S., Lee, K., Ju, B., Lee, J., Yu, S. J. et al. (2017). Identification of novelOCT4 genetic variant associated with the risk of chronic hepatitis B in a Korean population. *Liver International* 37, 354-361. doi:10.1111/liv.13245.

Somi, M. H., Najafi, L., Noori, B. N., Alizadeh, A. H., Aghah, M. R., Shavakhi, A. et al. (2006). Tumor necrosis factor-alpha gene promoter polymorphism in Iranian patients with chronic hepatitis B. *Indian J Gastroenterol* 25, 14-5.

Song, L. H., Toan, N. L., Xuan, N. T., Uhlemann, A., Boldt, A. B. W., Duy, D. N. et al. (2006). A promoter polymorphism in the interferon α-2 gene is associated with the clinical presentation of hepatitis B. *Mutation Research/Fundamental and Molecular Mechanisms of Mutagenesis* 601, 137-143. doi:10.1016/j.mrfmmm.2006.06.011.

Song, L. H., Xuan, N. T., Toan, N. L., Binh, V. Q., Boldt, A. B., Kremsner, P. G. et al. (2008). Association of two variants of the interferon-alpha receptor-1 gene with the presentation of hepatitis B virus infection. *Eur Cytokine Netw* 19, 204-10. doi:10.1684/ecn.2008.0137.

Song, Y., Shen, Y., Xia, X. and Zhang, A. (2017). Association between genetic polymorphisms of theIL28B gene and leukomonocyte in Chinese hepatitis B virus-infected individuals. *PeerJ* 5, e4149. doi:10.7717/peerj.4149.

Srivastava, M., Ranjan, A., Choudhary, J. K., Tripathi, M. K., Verma, S., Dixit, V. K. et al. (2014). Role of Proinflammatory Cytokines (Interferon Gamma) and Anti-Inflammatory Cytokine (Interleukin-10) Gene Polymorphisms in Chronic Hepatitis B Infection: An Indian Scenario. *Journal of Interferon & Cytokine Research* 34, 547-551. doi:10.1089/jir.2013.0054.

Su, Z., Li, Y., Liao, Y., Cai, B., Chen, J., Zhang, J. et al. (2016). Polymorphisms in sodium taurocholate cotransporting polypeptide are not associated with hepatitis B virus clearance in Chinese Tibetans and Uygurs. *Infection, Genetics and Evolution* 41, 128-134. doi:10.1016/j.meegid.2016.03.039.

Su, Z., Li, Y., Liao, Y., Cai, B., Chen, J., Zhang, J. et al. (2014). Association of the gene polymorphisms in sodium taurocholate cotransporting polypeptide with the outcomes of hepatitis B infection in Chinese Han population. *Infection, Genetics and Evolution* 27, 77-82. doi:10.1016/j.meegid.2014.07.001.

Sun, Y., Lu, Y., Li, T., Xie, L., Deng, Y., Li, S. et al. (2015). Interferon Gamma +874T/A Polymorphism Increases the Risk of Hepatitis Virus-Related Diseases: Evidence from a Meta-Analysis. *PLOS ONE* 10, e0121168. doi:10.1371/journal.pone.0121168.

Suneetha, P. V., Sarin, S. K., Goyal, A., Kumar, G. T., Shukla, D. K., Hissar, S. (2006). Association between vitamin D receptor, CCR5, TNF-α and TNF-β gene polymorphisms and HBV infection and severity of liver disease. *Journal of Hepatology* 44, 856-863. doi:10.1016/j.jhep.2006.01.028.

Sy, B. T., Hoan, N. X., Tong, H. V., Meyer, C. G., Toan, N. L., Song, L. H. et al. (2018). Genetic variants of interferon regulatory factor 5 associated with chronic hepatitis B infection. *World J Gastroenterol* 24, 248-256. doi:10.3748/wjg.v24.i2.248.

Talaat, R. M., Dondeti, M. F., El-Shenawy, S. Z. and Khamiss, O. A. (2014). Association Between IL-10 Gene Promoter Polymorphism and Hepatitis B Viral Infection in an Egyptian Population. *Biochemical Genetics* 52, 387-402. doi:10.1007/s10528-014-9655-8.

Tan, A., Gao, Y., Yao, Z., Su, S., Jiang, Y., Xie, Y. et al. (2016). Genetic variants in IL12 influence both hepatitis B virus clearance and HBV-related hepatocellular carcinoma development in a Chinese male population. *Tumor Biology* 37, 6343-6348. doi:10.1007/s13277-015-4520-x.

Thio, C. L., Astemborski, J., Thomas, R., Mosbruger, T., Witt, M. D., Goedert, J. J. et al. (2008). Interaction between RANTES promoter variant and CCR5Delta32 favors recovery from hepatitis B. *J Immunol* 181, 7944-7.

Thio, C. L., Mosbruger, T., Astemborski, J., Greer, S., Kirk, G. D., O'Brien, S. J. et al. (2005). Mannose Binding Lectin Genotypes Influence Recovery from Hepatitis B Virus Infection. *Journal of Virology* 79, 9192-9196. doi:10.1128/JVI.79.14.9192-9196.2005.

Thio, C. L., Mosbruger, T. L., Kaslow, R. A., Karp, C. L., Strathdee, S. A., Vlahov, D. et al. (2004). Cytotoxic T-Lymphocyte Antigen 4 Gene and Recovery from Hepatitis B Virus Infection. *Journal of Virology* 78, 11258-11262. doi:10.1128/JVI.78.20.11258-11262.2004.

Thio, C. L., Astemborski, J., Bashirova, A., Mosbruger, T., Greer, S., Witt, M. D. et al. (2007). Genetic Protection against Hepatitis B Virus Conferred by CCR5 32: Evidence that CCR5 Contributes to Viral Persistence. *J Virol* 81, 441-445. doi:10.1128/JVI.01897-06.

Thomas, R., Thio, C. L., Apps, R., Qi, Y., Gao, X., Marti, D. et al. (2012). A Novel Variant Marking HLA-DP Expression Levels Predicts Recovery from Hepatitis B Virus Infection. *Journal of Virology* 86, 6979-6985. doi:10.1128/JVI.00406-12.

Trinks, J., Nishida, N., Hulaniuk, M. L., Caputo, M., Tsuchiura, T., Marciano, S. et al. (2017). Role of HLA-DP and HLA-DQ on the clearance of hepatitis B virus and the risk of chronic infection in a multiethnic population. *Liver International* 37, 1476-1487. doi:10.1111/liv.13405.

Vermehren, J., Lötsch, J., Susser, S., Wicker, S., Berger, A., Zeuzem, S. et al. (2012). A Common HLA-DPA1 Variant Is Associated with Hepatitis B Virus Infection but Fails to Distinguish Active from Inactive Caucasian Carriers. *PLoS ONE* 7, e32605. doi:10.1371/journal.pone.0032605.

Wang, L., Wu, X., Zhang, W., Zhu, D., Wang, Y., Li, Y. et al. (2011). Evaluation of Genetic Susceptibility Loci for Chronic Hepatitis B in Chinese: Two Independent Case-Control Studies. *PLoS ONE* 6, e17608. doi:10.1371/journal.pone.0017608.

Wang, P., Mo, R., Lai, R., Xu, Y., Lu, J., Zhao, G. et al. (2017). Genetic variations of NTCP are associated with susceptibility to HBV infection and related hepatocellular carcinoma. *Oncotarget* 8, 105407-105424. doi:10.18632/oncotarget.22211.

Wang, Z. S., Yin, C. C., Han, S., Jiang, F. L., Guo, W. G., Wu, L. Q. et al. (2015). -173G/C polymorphism in the promoter of MIF is associated with hepatitis B virus infection in a Chinese Han population. *Genet Mol Res* 14, 8532-8. doi:10.4238/2015.July.28.23.

Wasityastuti, W., Yano, Y., Ratnasari, N., Triyono, T., Triwikatmani, C., Indrarti, F. et al. (2016). Protective effects of HLA-DPA1/DPB1 variants against Hepatitis B virus infection in an Indonesian population. *Infection, Genetics and Evolution* 41, 177-184. doi:10.1016/j.meegid.2016.03.034.

Wen, J., Liu, Y., Liu, J., Liu, L., Song, C., Han, J. et al. (2015). Expression quantitative trait loci in long non-coding RNA ZNRD1-AS1 influence both HBV infection and hepatocellular carcinoma development. *Molecular Carcinogenesis* 54, 1275-1282. doi:10.1002/mc.22200.

Wong, D. K., Watanabe, T., Tanaka, Y., Seto, W., Lee, C., Fung, J. et al. (2013). Role of HLA-DP Polymorphisms on Chronicity and Disease Activity of Hepatitis B Infection in Southern Chinese. *PLoS ONE* 8, e66920. doi:10.1371/journal.pone.0066920.

Wu, S., Zhou, X., Yang, H., Yin, J., Cai, C., Zheng, F. (2009). Polymorphisms and plasma soluble levels of E-selectin in patients with chronic hepatitis B virus infection. *Clinical Chemistry and Laboratory Medicine* 47, doi:10.1515/CCLM.2009.035.

Wu, W., Zeng, Y., Lin, J., Wu, Y., Chen, T., Xun, Z. et al. (2018). Genetic variants inNTCP exon gene are associated with HBV infection status in a Chinese Han population. *Hepatology Research* 48, 364-372. doi:10.1111/hepr.13007.

Xia, P., Zhou, M., Dong, D. S., Xing, Y. and Bai, Y. (2014). Association of polymorphisms in interleukin-18 and interleukin-28B genes with outcomes of hepatitis B virus infections: a meta-analysis. *Tumor Biology* 35, 1129-1137. doi:10.1007/s13277-013-1151-y.

Xiang, X., Guo, Y., Yang, L., Ge, Q., Mijit, S., Xu, F. (2016). Association of human leukocyte antigen DP/DQ gene polymorphisms with chronic hepatitis B in Chinese Han and Uygur populations. *Infection, Genetics and Evolution* 43, 407-411. doi:10.1016/j.meegid.2016.06.022.

Xiang, Y., Huang, S. F., Xia, J. R., Ye, D. Q., Chen, P., Yang, S. S. et al. (2014). Association of the IFNAR1-17470 and IL-10-592 cytokine variants with susceptibility to chronic hepatitis B viral infections in a Chinese population. *Genetics and molecular research : GMR* 13, 9187.

Xi-Lin, Z., Te, D., Jun-Hong, L., Liang-Ping, L., Xin-Hui, G., Ji-Rong, G. et al. (2006). Analysis of HLA-DQB1 gene polymorphisms in asymptomatic HBV carriers and chronic hepatitis B patients in the Chinese Han population. *International Journal of Immunogenetics* 33, 249-254. doi:10.1111/j.1744-313X.2006.00607.x.

Xu, H., Zhao, M., He, J. and Chen, Z. (2013). Association between cytotoxic T-lymphocyte associated protein 4 gene +49 A/G polymorphism and chronic infection with hepatitis B virus: A meta-analysis. *Journal of International Medical Research* 41, 559-567. doi:10.1177/0300060513483387.

Xu, J., Zhang, S., Zhang, Z., Fu, L., Zheng, Q., Wang, J. et al. (2013). TNF-alpha promoter region polymorphisms affect HBV virus clearance in southern Chinese. *Clinica Chimica Acta* 425, 90-92. doi:10.1016/j.cca.2013.07.015.

Xu, T., Sun, M. and Wang, H. (2017). Relationship between HLA-DQ Gene Polymorphism and Hepatitis B Virus Infection. *BioMed Research International* 2017, 1-11. doi:10.1155/2017/9679843.

Xu, T., Zhu, A., Sun, M., Lv, J., Qian, Z., Wang, X. et al. (2018). Quantitative assessment of HLA-DQ gene polymorphisms with the development of hepatitis B virus infection, clearance, liver cirrhosis, and hepatocellular carcinoma. *Oncotarget* 9, 96-109. doi:10.18632/oncotarget.22941.

Chen, X., Wang, Y., Chen, X., Cheng, K., Li, J., Lou, J.,et al. (2016). Genetic variants in the regulatory region of SLC10A1 are not associated with the risk of hepatitis B virus infection and clearance. *Infect Genet Evol 44,* 495-500.

Yan, F. (2012). No association betweenIRF3 polymorphism and susceptibility to hepatitis B virus infection in Chinese patients. *World Journal of Gastroenterology* 18, 388. doi:10.3748/wjg.v18.i4.388.

Yan, Z., Tan, S., Dan, Y., Sun, X., Deng, G., Wang, Y. (2012). Relationship between HLA-DP gene polymorphisms and clearance of chronic hepatitis B virus infections: Case–control study and meta-analysis. *Infection, Genetics and Evolution* 12, 1222-1228. doi:10.1016/j.meegid.2012.03.026.

Yang, J., Yang, Y., Xia, M., Wang, L., Zhou, W., Yang, Y. et al. (2016). A genetic variant of the NTCP gene is associated with HBV infection status in a Chinese population. *BMC Cancer* 16, doi:10.1186/s12885-016-2257-6.

Yao, J.Y., Chao, K., Li, M.R., Wu, Y.Q., Zhong, B.H., (2015). Interleukin-21 gene polymorphisms and chronic hepatitis B infection in a Chinese population. *World J Gastroenterol* 21, 4232. doi:10.3748/wjg.v21.i14.4232.

Han, Y., Jiang, ZY., Jiao, LX., Yao, C., Lin, QF., Ma, N. et al. (2012). Association of human leukocyte antigen-DRB1 alleles with chronic hepatitis B virus infection in the Han Chinese of Northeast China.*Mol Med Rep* 5, 1347-1351.

Yu, L., Cheng, Y., Cheng, M., Yao, Y., Zhang, Q., Zhao, X. et al. (2015). Quantitative assessment of common genetic variations in HLA-DP with hepatitis B virus infection, clearance and hepatocellular carcinoma development. *Scientific Reports* 5, 14933. doi:10.1038/srep14933.

Yu, S. J., Kim, J. W., Lee, J. H., Yoon, J. H., Lee, H. S., Cheong, J. Y. et al. (2014). Association of a microRNA-323b polymorphism with the persistence of hepatitis B virus infection by the enhancement of viral replication. *Journal of Viral Hepatitis* 21, 853-859. doi:10.1111/jvh.12215.

Zhang, G., Han, Q., Duan, S., Li, Z., Li, N., Zhu, Q. et al. (2012). PDCD1 polymorphism amplifies the predisposing effect conferred by CTLA4 polymorphism in chronic hepatitis B virus infection. *Human Immunology* 73, 421-425. doi:10.1016/j.humimm.2012.01.013.

Zhang, P. A., Li, Y., Xu, P. and Wu, J. M. (2004). Polymorphisms of interleukin-1B and interleukin-1 receptor antagonist genes in patients with chronic hepatitis B. *World J Gastroenterol* 10, 1826-9.

Zhang, P. A., Wu, J. M., Li, Y. and Yang, X. S. (2005). Association of polymorphisms of interleukin-18 gene promoter region with chronic hepatitis B in Chinese Han population. *World J Gastroenterol* 11, 1594-8.

Zhang, Q., Yin, J., Zhang, Y., Deng, Y., Ji, X., Du, Y. et al. (2013). HLA-DP Polymorphisms Affect the Outcomes of Chronic Hepatitis B Virus Infections, Possibly through Interacting with Viral Mutations. *Journal of Virology* 87, 12176-12186. doi:10.1128/JVI.02073-13.

Zhang, Q., Ji, X. W., Hou, X. M., Lu, F. M., Du, Y., Yin, J. H. et al. (2014). Effect of functional nuclear factor-kappaB genetic polymorphisms on hepatitis B virus persistence and their interactions with viral mutations on the risk of hepatocellular carcinoma. *Annals of Oncology* 25, 2413-2419. doi:10.1093/annonc/mdu451.

Zhang, Q. X., Li, S. L., Yao, Y. Q. and Li, T. J. (2016). Association between interleukin-21 gene polymorphisms (rs12508721) and HBV-related hepatocellular carcinoma. *International Journal of Immunogenetics* 43, 151-158. doi:10.1111/iji.12263.

Zhang, T., Liu, W., Liu, X., Pan, F., Gao, Y., Yan, F. et al. (2013). Single nucleotide polymorphisms rs2120131, rs4935047, and rs7095891 in theMBL2 gene show no association with susceptibility to chronic hepatitis B in a Chinese Han population. *Journal of Medical Virology* 85, 602-607. doi:10.1002/jmv.23514.

Zhang, X., Sun, X. H., Li, M., Zhou, Z. H. and Gao, Y. Q. (2014). A promoter polymorphism (rs3806798) of interleukin-15 gene is associated with chronic hepatitis B virus infection in the Chinese Han population. *International Journal of Immunogenetics* 41, 298-305. doi:10.1111/iji.12120.

Zhang, X., Jia, J., Dong, J., Yu, F., Ma, N., Li, M. et al. (2014). HLA-DQ polymorphisms with HBV infection: different outcomes upon infection and prognosis to lamivudine therapy. *Journal of Viral Hepatitis* 21, 491-498. doi:10.1111/jvh.12159.

Zhang, X., Ni, X., Jia, J., Dong, J., Yu, F., Ma, N. et al. (2013). Association of the rs3077 and rs9277535 polymorphisms inHLA-DP with hepatitis B virus infection and spontaneous clearance: A meta-analysis. *Scandinavian Journal of Gastroenterology* 48, 736-744. doi:10.3109/00365521.2013.787643.

Zhang, Y., Li, Y., Wu, M., Cao, P., Liu, X., Ren, Q. et al. (2017). Comprehensive assessment showed no associations of variants at the SLC10A1 locus with susceptibility to persistent HBV infection among Southern Chinese. *Scientific Reports* 7, doi:10.1038/srep46490.

Zhao, N., Wang, X., Gu, Q., Huang, F., Zheng, W., Li, Z. (2014). Tripartite Motif-Containing 22 Gene -364T/C Polymorphism Associated With Hepatitis B Virus Infection in Chinese Han Population. *Hepatitis Monthly* 14, doi:10.5812/hepatmon.12110.

Zhao, Q., Peng, L., Huang, W., Li, Q., Pei, Y., Yuan, P. et al. (2012). Rare inborn errors associated with chronic hepatitis B virus infection*. *Hepatology* 56, 1661-1670. doi:10.1002/hep.25850.

Zheng, M. H., Xiao, D. D., Lin, X. F., Wu, S. J., Peng, M. M., Yu, X. Y. et al. (2012). The tumour necrosis factor-α-238A allele increases the risk of chronic HBV infection in European populations. *Journal of Viral Hepatitis* 19, e11-e17. doi:10.1111/j.1365-2893.2011.01491.x.

Zhou, G. Q., Meng, H., Wang, J. R., Sun, F. X., Wang, X. J., Wang, R. B. et al. (2015). Functional polymorphisms in microRNA gene and hepatitis B risk among Asian population: a meta-analysis. *Genetics and Molecular Research* 14, 4767-4777. doi:10.4238/2015.May.11.9.

Zhou, J., Smith, D. K., Lu, L., Poon, V. K. M., Ng, F., Chen, D. Q. et al. (2009). A non-synonymous single nucleotide polymorphism in IFNAR1 affects susceptibility to chronic hepatitis B virus infection. *Journal of Viral Hepatitis* 16, 45-52. doi:10.1111/j.1365-2893.2008.01040.x.

Zhou, J., Lu, L., Yuen, M., Lam, T., Chung, C., Lam, C. et al. (2007). Polymorphisms of type I interferon receptor 1 promoter and their effects on chronic hepatitis B virus infection. *Journal of Hepatology* 46, 198-205. doi:10.1016/j.jhep.2006.08.017.

Zhu, J., Zhang, T., Cao, L., Li, A., Zheng, K., Zhang, N. et al. (2017). Toll like receptor7 polymorphisms in relation to disease susceptibility and progression in Chinese patients with chronic HBV infection. *Scientific Reports* 7, doi:10.1038/s41598-017-12698-5.

Zhu, M., Dai, J., Wang, C., Wang, Y., Qin, N., Ma, H. et al. (2016). Fine mapping the MHC region identified four independent variants modifying susceptibility to chronic hepatitis B in Han Chinese. *Hum Mol Genet* 25, 1225-32. doi:10.1093/hmg/ddw003.

Zhu, Q., Li, N., Han, Q., Li, Z., Zhang, G., Li, F. et al. (2012). Single-Nucleotide Polymorphism atCYP27B1-1260, but NotVDR TaqI, Is Possibly Associated with Persistent Hepatitis B Virus Infection. *Genetic Testing and Molecular Biomarkers* 16, 1115-1121. doi:10.1089/gtmb.2012.0148.

Zhu, X. L., Du T., Li, J. H., Lu, L. P., Guo, X. H., Gao, J. R. et al. (2007). Association of HLA-DQB1 gene polymorphisms with outcomes of HBV infection in Chinese Han population. *Swiss Med Wkly* 137, 114-20. doi:2007/07/smw-11428.

Zidi, I., Laaribi, A. B., Bortolotti, D., Belhadj, M., Mehri, A., Yahia, H. B. et al. (2016). HLA-E polymorphism and soluble HLA-E plasma levels in chronic hepatitis B patients. *HLA* 87, 153-159. doi:10.1111/tan.12767.
