# Supplementary material for: Host Genetic Determinants of Hepatitis B Virus Infection
Source: Front Genet. 2019 Aug 13;10:696. doi: 10.3389/fgene.2019.00696 (PMC6702792; doi:10.3389/fgene.2019.00696)
Supplement: Supplementary file 3 [file Table_3.doc]

**Supplement Table S3. Selected studies on host genetic factors associated with susceptibility to intrauterine hepatitis B infection.**

| **Genes** | **Gentic determinants**  **SNP/Hap/CNVs** | **Population**  **/Region** | **Case^a^**  **(n)** | **Control^b^**  **(n)** | **Methods** | **Disease association** | **OR (95% CI)** | **P value** | **Reference** |
| --- | --- | --- | --- | --- | --- | --- | --- | --- | --- |
| APOBEC3B | rs2076109 | Chinese | 69 | 138 | PCR | No |  |  | Liu et al. 2018 |
| CD274 | rs822336 |  |  |  |  | No |  |  |  |
|  | rs2297136 |  |  |  |  | No |  |  |  |
|  | rs4143815 |  |  |  |  | No |  |  |  |
| CD40 | rs1883832 |  |  |  |  | No |  |  |  |
|  | rs1800686 |  |  |  |  | No |  |  |  |
| CD40LG | rs1126535 |  |  |  |  | No |  |  |  |
| CXCL13 | rs355687 | Chinese | 44 | 662 | PCR | Yes | 0.25 ( 0.08-0.82) | 0.022 | Wan et al. 2016 |
| CXCR5 | rs3922 |  |  |  |  | No |  |  |  |
| DC-SIGN | 7/5 | Chinese | 29 | 54 | PCR | Yes |  | 0.038 | Liu et al. 2018 |
| DC-SIGNR | Non 7/5 genotype |  |  |  |  | No |  |  |  |
| HLA-C | rs3130542 | Chinese | 44 | 662 | PCR | No |  |  | Wan et al. 2016 |
| HLA-DP | rs3128917 |  |  |  |  | No |  |  |  |
| HLA-DRB1 | *07 | Chinese | 24 | 48 | PCR | Yes | 6.66 (1.77-24.59) | 0.004 | Xu et al. 2008 |
|  | *01 |  |  |  |  | No |  |  |  |
|  | *03 |  |  |  |  | No |  |  |  |
|  | *04 |  |  |  |  | No |  |  |  |
|  | *08 |  |  |  |  | No |  |  |  |
|  | *09 |  |  |  |  | No |  |  |  |
|  | *10 |  |  |  |  | No |  |  |  |
|  | *11 |  |  |  |  | No |  |  |  |
|  | *12 |  |  |  |  | No |  |  |  |
|  | *14 |  |  |  |  | No |  |  |  |
|  | *15 |  |  |  |  | No |  |  |  |
|  | *16 |  |  |  |  | No |  |  |  |
| HLA-DR | B3* |  |  |  |  | No |  |  |  |
|  | B4* |  |  |  |  | No |  |  |  |
|  | B5* |  |  |  |  | No |  |  |  |
| IFN-γ | (CA12)+/(CA12)^+^ | Chinese | 46 | 73 | PCR | Yes |  | 0.018 | Yu et al. 2006b |
|  | +874 |  |  |  |  | Yes | 2.23 (1.24-3.99) | 0.024 |  |
|  |  | Chinese | 46 | 251 | PCR | Yes |  | <0.05 | Zhu et al. 2005 |
| IL-10 | -1082 |  |  |  |  | Yes |  | < 0.001 |  |
| IL-4 | -590 |  |  |  |  | No |  |  |  |
| LTA | rs1041981 | Chinese | 69 | 138 | PCR | No |  |  | Liu et al. 2018 |
|  | rs1800630 |  |  |  |  | No |  |  |  |
|  | rs2239704 |  |  |  |  | No |  |  |  |
| LTBR | rs2364480 |  |  |  |  | No |  |  |  |
|  | s3759333 |  |  |  |  | No |  |  |  |
| NTCP | rs2296651 | Chinese | 44 | 662 | PCR | No |  |  | Wan et al. 2016 |
|  | rs7154439 |  |  |  |  | No |  |  |  |
| PDCD1 | rs2227981 | Chinese | 69 | 138 | PCR | Yes |  | 0.009 | Liu et al. 2018 |
| TLR-3 | rs3775290 | Chinese | 399 |  | PCR | Yes | 0.55 (0.34-0.91) | 0.02 | Gao et al. 2015 |
|  | rs3775291 | Chinese | 44 | 662 | PCR | No |  |  | Wan et al. 2016 |
| TLR-4 | rs1927914 | Chinese | 44 | 662 | PCR | No |  |  | Wan et al. 2016 |
| TLR-9 | rs352140 | Chinese | 399 |  | PCR | Yes | 0.62 ( 0.39-1.00) | 0.048 | Gao et al. 2015 |
|  |  | Chinese | 44 | 662 | PCR | No |  |  | Wan et al. 2016 |
| TNFSF14 | rs3760746 | Chinese | 69 | 138 | PCR | No |  |  | Liu et al. 2018 |
|  | rs8106574 |  |  |  |  | No |  |  |  |
| TNF-α | -238 | Chinese | 46 | 251 | PCR | Yes |  | < 0.05 | Zhu et al. 2005 |
|  |  | Chinese | 45 | 211 | PCR | Yes |  | 0.009 | Gu et al. 2004 |
| UBE2L3 | rs4821116 | Chinese | 44 | 662 | PCR | No |  |  | Wan et al. 2016 |

**Note:**

1. Case: Numbers of neonates positive for HBsAg and/or HBV DNA born to HBsAg-positive mothers; b. Control: numbers of neonates negative for HBsAg born to HBsAg-positive mothers; in a minority of the studies, the numbers of HBV infected neonates and controls were not separately given. For these studies, the total number of all subjects included were given in the case column.; SNP, single nucleotide polymorphism; HBV, hepatitis B virus; OR (95% CI), odds ratio (95% confidence interval); Yes, positive result reported; No, not statistical significance; PCR, polymerase chain reaction-based research methods; Population, including race or region.

**References:**

Gao, Y., Guo, J., Zhang, F., Guo, Z., Zhang, L. R., Wang, T. et al. (2015). Evaluation of neonatal Toll-like receptors 3 (c.1377C/T) and 9 (G2848A) gene polymorphisms in HBV intrauterine transmission susceptibility. *Epidemiol Infect* 143, 1868-75. doi:10.1017/S0950268814002921.

Gu, S. Q., Zhu, Q. R., Yu, H., Fei, L. E., Dong, Z. Q., Pu, D. P. (2004). Relationship between genetic polymorphism of tumor necrosis factor-alpha and susceptibility to intrauterine HBV infection. *Zhonghua Gan Zang Bing Za Zhi* 12, 538-9.

Liu, S., Weng, H., Wu, J., Zhang, Z., Zeng, Y., Tian, H. (2011). Relationship between intrauterine infection and the gene polymorphism of DC-SIGN/DC-SIGNR in the pregnant women of HBV positive. *Zhonghua Shi Yan He Lin Chuang Bing Du Xue Za Zhi* 25, 331.

Liu, T., Wan, Z., Peng, S., Wang, Y., Chen, H., Li, X. et al. (2018). Genetic variations in LTA gene and PDCD1 gene and intrauterine infection of hepatitis B virus: a case-control study in China. *Amino Acids* 50, 877-883. doi:10.1007/s00726-018-2568-9.

Wan, Z., Lin, X., Li, T., Zhou, A., Yang, M., Hu, D. et al. (2016). Genetic variant in CXCL13 gene is associated with susceptibility to intrauterine infection of hepatitis B virus. *Sci Rep* 6, 26465. doi:10.1038/srep26465.

Xu, Y. Y., Yu, J. Y., Zhong, Y. W., Song, H. B., Liu, H. H., Jia, L. L. et al. (2008). Association between the frequency of class II HLA antigens and the susceptibility to intrauterine infection of hepatitis B virus. *Int J Biol Sci* 4, 111-5.

Yu, H., Zhu, Q. R., Gu, S. Q. and Fei, L. E. (2006). Relationship between IFN-gamma gene polymorphism and susceptibility to intrauterine HBV infection. *World J Gastroenterol* 12, 2928-31.

Zhu, Q. R., Gu, S. Q., Yu, H., Wang, J. S., Gu, X. H., Dong, Z. Q. et al. (2005). Relationship between cytokine gene polymorphism and susceptibility to hepatitis B virus intrauterine infection. *Zhonghua Liu Xing Bing Xue Za Zhi* 26, 236-9.
