# Supplementary material for: Host Genetic Determinants of Hepatitis B Virus Infection
Source: Front Genet. 2019 Aug 13;10:696. doi: 10.3389/fgene.2019.00696 (PMC6702792; doi:10.3389/fgene.2019.00696)
Supplement: Supplementary file 4 [file Table_4.doc]

**Supplement Table S4. Selected studies on host genetic factors associated with occult hepatitis B virus infection.**

| **Genes** | **Gentic determinants**  **SNP/Hap/CNVs** | **Population**  **/Region** | **Case^a^**  **(n)** | **Control^b^**  **(n)** | **Methods** | **Disease association** | **OR (95% CI)** | **P value** | **Reference** |
| --- | --- | --- | --- | --- | --- | --- | --- | --- | --- |
| CTNNB1 | R72 | Italian | 34 | 27 | PCR | No |  |  | Saitta et al.  2015 |
|  | R72P |  |  |  |  | No |  |  |  |
|  | P72 |  |  |  |  | No |  |  |  |
| CXCL12 | +801 | Iranian | 57 | 100 | PCR | Yes |  | <0.001 | Hassanshahi et al.  2010 |
| FAS | -670 | Iranian | 57 | 100 | PCR | No |  |  | Arababadi et al.  2011 |
| HLA-B | *44:03 | Chinese | 107 | 280 | PCR | Yes | 2.15 (1.07-4.31) | 0.028 | Wang et al.  2017 |
| HLA-C | *07:01 |  |  |  |  | Yes | 4.69 (1.82-12.09) | <0.001 |  |
| HLA-DP | rs3077 | Indonesian | 17 | 105 | PCR | Yes | 3.87 (1.58-9.49) | 0.0015 | Mardian et al.  2017 |
|  | T-G-A ^1^ |  |  |  |  | Yes | 4.90 (1.12-21.52) | 0.038 |  |
|  | rs3135021 |  |  |  |  | No |  |  |  |
|  | rs9277535 |  |  |  |  | No |  |  |  |
|  | rs2281388 |  |  |  |  | No |  |  |  |
| HLA-DQB1 | *02:02 | Chinese | 107 | 280 | PCR | Yes | 1.92 (1.19-3.10) | 0.007 | Wang et al.  2017 |
|  | *06:02 |  |  |  |  | Yes | 0.50 (0.25-1.01) | 0.048 |  |
| HLA-DRB1 | *07:01 | Chinese | 107 | 280 | PCR | Yes | 2.01 (1.30-3.11) | 0.001 | Wang et al.  2017 |
|  | *08:03 |  |  |  |  | Yes | 0.40 ( 0.15-1.03) | 0.049 |  |
|  | *15:01 |  |  |  |  | Yes | 0.50 (0.26-0.94) | 0.029 |  |
| IFN-γ | +874 | Iranian | 57 | 100 | PCR | No |  |  | Arababadi et al.  2011 |
| IL-10 | -592 | Iranian | 57 | 100 | PCR | Yes |  | 0.001 | Ahmadabadi et al.  2012 |
| IL-12 | +1188 | Iranian | 57 | 100 | PCR | No |  |  | Arababadi et al.  2011 |
| IL-28B | rs12979860 | Spanish | 34 | 83 | PCR | No |  |  | Bes et al.  2015 |
| TP53 | codon 72 | Italian | 34 | 27 | PCR | No |  |  | Saitta et al.  2015 |
| VDR | exon 9 | Iranian | 57 | 57 | PCR | Yes |  | <0.049 | Arababadi et al.  2010 |
|  | intron 8 |  |  |  |  | No |  |  |  |

**Note:**

1. Case: Occult HBV infection patients; b. Control: Healthy controls; SNP, single nucleotide polymorphism; HBV, hepatitis B virus; OR (95% CI), odds ratio (95% confidence interval); Yes, positive result reported; No, not statistical significance; PCR, polymerase chain reaction-based research methods; Population, including race or region.

**Haplotype**

1. T-G-A, rs3077-rs3135021-rs9277535

**References:**

Ahmadabadi, B. N., Hassanshahi, G., Arababadi, M. K., Leanza, C. and Kennedy, D. (2012). The IL-10 promoter polymorphism at position -592 is correlated with susceptibility to occult HBV infection. *Inflammation* 35, 818-21. doi:10.1007/s10753-011-9381-x.

Arababadi, M. K., Mohammadzadeh, A., Pourfathollah, A. A. and Kennedy, D. (2011). Polymorphisms within Fas gene are not associated with occult hepatitis B virus infection: Polymorphisms within Fas gene in occult HBV infection. *Hepat Mon* 11, 23-6.

Arababadi, M. K., Pourfathollah, A. A., Jafarzadeh, A., Hassanshahi, G. and Rezvani, M. E. (2010). Association of exon 9 but not intron 8 VDR polymorphisms with occult HBV infection in south-eastern Iranian patients. *J Gastroenterol Hepatol* 25, 90-3. doi:10.1111/j.1440-1746.2009.05950.x.

Arababadi, M. K., Pourfathollah, A. A., Jafarzadeh, A., Hassanshahi, G., Daneshmandi, S., Shamsizadeh, A. et al. (2011). Non-association of IL-12 +1188 and IFN-gamma +874 polymorphisms with cytokines serum level in occult HBV infected patients. *Saudi J Gastroenterol* 17, 30-5. doi:10.4103/1319-3767.74461.

Bes, M., Vargas, V., Piron, M., Casamitjana, N., Esteban, J. I., Campos-Varela, I. et al. (2015). Doubtful Role of IL28B Polymorphism in Occult Hepatitis B Infection. *Intervirology* 58, 160 - 165. doi:10.1159/000430444.

Hassanshahi, G., Arababadi, M. K., Khoramdelazad, H., Yaghini, N. and Zarandi, E. R. (2010). Assessment of CXCL12 (SDF-1a) polymorphisms and its serum level in posttransfusion occult HBV-infected patients in Southeastern Iran. *Arch Med Res* 41, 338-42. doi:10.1016/j.arcmed.2010.07.001.

Mardian, Y., Yano, Y., Wasityastuti, W., Ratnasari, N., Liang, Y., Putri, W. A. et al. (2017). Genetic polymorphisms of HLA-DP and isolated anti-HBc are important subsets of occult hepatitis B infection in Indonesian blood donors: a case-control study. *Virol J* 14, 201. doi:10.1186/s12985-017-0865-7.

Saitta, C., Lanza, M., Bertuccio, A., Lazzara, S., Navarra, G., Raimondo, G. et al. (2015). Evaluation of CTNNB1 and TP53 variability in patients with hepatocellular carcinoma and occult hepatitis B virus infection. *Cancer Genet* 208, 513-6. doi:10.1016/j.cancergen.2015.07.002.

Wang, T., Shen, C., Chen, L., Liu, S. and Ji, Y. (2017). Association of HLA polymorphisms with occult hepatitis B virus infection in a Shaanxi Han population. *Journal of Gene Medicine* 19, e2987 - n/a. doi:10.1002/jgm.2987.
