# Supplementary material for: Host Genetic Determinants of Hepatitis B Virus Infection
Source: Front Genet. 2019 Aug 13;10:696. doi: 10.3389/fgene.2019.00696 (PMC6702792; doi:10.3389/fgene.2019.00696)
Supplement: Supplementary file 5 [file Table_5.doc]

**Supplement Table S5. Selected studies on host genetic factors associated response to IFN-α treatment of chronic hepatitis B patients.**

| **Genes** | **Gentic determinants**  **SNP/Hap/CNVs** | **Population**  **/Region** | **Non-responder (n)** | **Responder**  **(n)** | **Methods** | **Disease association** | **OR (95% CI)** | **P value** | **Reference** |
| --- | --- | --- | --- | --- | --- | --- | --- | --- | --- |
| ADAR | exon 15-1 | Taiwan | 46 | 36^d^ | PCR | No |  |  | King et a1.  2002 |
|  | exon 15-2 |  |  |  |  | No |  |  |  |
|  | exon 15-3 |  |  |  |  | No |  |  |  |
|  | rs1127314 | Chinese | 77 | 169^d^ | PCR | No |  |  | Wu et al.  2009 |
|  | rs3738032 |  |  |  |  | No |  |  |  |
|  | rs3766924 |  |  |  |  | No |  |  |  |
| ADAR1 | rs1127313 | Chinese | 358 | 190^a^ | MassArray | Yes | 0.71 (0.55-0.92) | 0.008 | Wu et al.  2012 |
|  | rs4636449 |  |  |  |  | Yes | 0.52 (0.35-0.79) | 0.001 |  |
|  | rs4845384 |  |  |  |  | Yes | 0.62 (0.46-0.83) | 0.0007 |  |
|  | rs7531982 |  |  |  |  | Yes | 0.69 (0.54-0.90) | 0.004 |  |
| CYP27B1 | rs10877012 | Thailand | 80 | 31^c^ | PCR | No |  |  | Thanapirom et al.  2017 |
|  |  | Italy | 91 | 82^e^ | PCR | Yes | 0.32 (0.17-0.60) | <0.001 | Cusato et al.  2017 |
|  |  | Chinese | 29 | 16^f^ | PCR | Yes | 9.92 (3.08-31.98) | <0.001 | Wu et al.  2018 |
|  | rs4646536 | Italy | 129 | 61^e^ | PCR | Yes | 3.13 (1.71-5.76) | <0.001 | Boglione et al. 2015 |
|  |  | Thailand | 92 (HBeAg positive) | 30^a^ | PCR | Yes | 2.48 (1.07-5.76) | 0.034 | Limothai et al.  2017 |
|  |  |  | 123 (HBeAg negative) | 30^e^ | PCR | Yes | 3.36 (1.47-7.65) | 0.008 |  |
|  |  | Italy | 91 | 82^e^ | PCR | Yes | 2.87 (1.52-5.40) | 0.001 | Cusato et al.  2017 |
|  |  | Chinese | 29 | 16^f^ | PCR | Yes | 3.25 (1.32-7.98) | 0.009 | Wu et al.  2018 |
| CYP2R1 | rs12794714 | Thailand | 80 | 31^c^ | PCR | Yes | 4.53 (1.51-13.61) | 0.01 | Thanapirom et al.  2017 |
|  | rs2060793 |  |  |  |  | No |  |  |  |
| CYP24A1 | rs2248359 | Italy | 91 | 82^e^ | PCR | Yes | 2.09 (1.02-4.27) | 0.043 | Cusato et al.  2017 |
|  | rs927650 |  |  |  |  | No |  |  |  |
|  | rs2585428 |  |  |  |  | No |  |  |  |
| DBP | rs7041 | Thailand | 92 (HBeAg positive) | 30^a^ | PCR | No |  |  | Limothai et al.  2017 |
|  |  |  | 123 (HBeAg negative) | 30^e^ | PCR | No |  |  |  |
| DHCR7 | rs12785878 | Thailand | 80 | 31^c^ | PCR | No |  |  | Thanapirom et al.  2017 |
| eIF-2S1 | rs3759756 | Chinese | 77 | 169^d^ | PCR | No |  |  | Wu et al.  2009 |
| eIF-2α | reg 1 | Taiwan | 46 | 36^d^ | PCR | No |  |  | King et a1.  2002 |
|  | reg 2 |  |  |  |  | Yes | 12.82 (1.52-107.85) | 0.009 |  |
|  | reg1/reg2 |  |  |  |  | Yes |  | 0.015 |  |
| ESR1 | rs2077647 | Chinese | 56 | 31^e^ | MassARRAY | No |  |  | Zhang et al.  2016 |
|  | rs2234693 |  |  |  |  | No |  |  |  |
|  | rs9322354 |  |  |  |  | No |  |  |  |
|  | rs9340799 |  |  |  |  | No |  |  |  |
|  | T29C |  | 100/NR | | PCR | Yes |  | <0.05 | Zhang et al.  2010 |
| GBP2 | exon 05-1 | Taiwan | 46 | 36^d^ | PCR | No |  |  | King. et a1.  2002 |
|  | exon 05-2 |  |  |  |  | No |  |  |  |
| G3BP2 | rs3821977 | Europe,  Asia and North America | 253 | 161^e^ | GWAS | Yes |  | 2.46×10^-6^ | Brouwer et al.  2019 |
| GC | rs222020 | Thailand | 80 | 31^c^ | PCR | No |  |  | Thanapirom et al.  2017 |
|  | rs2282679 |  |  |  |  | No |  |  |  |
|  | rs4588 |  |  |  |  | No |  |  |  |
|  | rs7041 |  |  |  |  | No |  |  |  |
| HLA-A, B, C | *1101-*4601-*0102 | Chinese | 66 | 152^d^ | PCR | Yes | 4.84 (1.29-19.48) | 0.009 | Zhu et al.  2011 |
| HLA-C | rs2524099 | Netherlands | 86/NR | | PCR | No |  |  | Stelma et al.  2016 |
|  | rs2853951 |  |  |  |  | No |  |  |  |
|  | rs9394047 |  |  |  |  | No |  |  |  |
|  | rs34131062 |  |  |  |  | No |  |  |  |
|  | rs2524096 |  |  |  |  | No |  |  |  |
|  | rs2001181 |  |  |  |  | No |  |  |  |
|  | rs9264601 |  |  |  |  | No |  |  |  |
|  | rs9264602 |  |  |  |  | No |  |  |  |
|  | rs1131096 |  |  |  |  | No |  |  |  |
|  | rs9366775 |  |  |  |  | No |  |  |  |
|  | rs2074489 |  |  |  |  | No |  |  |  |
|  | rs2308557 |  |  |  |  | Yes |  | 0.003 |  |
| KIR/ HLA-C | KIR2/DL1-C2 | Netherlands | 86/NR | | PCR | Yes | 18.91 (2.15-166.28) | 0.002 | Stelma et al.  2016 |
| HLA-DP | rs3077 | Caucasian | 205 | 57^e^ | PCR | No |  |  | Brouwer et al.  2014 |
|  |  | Chinese | 94 | 50^c^ | PCR | Yes | 2.36 (1.11-5.06) | 0.027 | Cheng et al.  2014 |
|  |  | Thailand | 62 | 45^c^ | PCR | No |  |  | Limothai et al.  2016 |
|  |  | Thailand | 76 | 50^e^ | PCR | Yes | 2.78 (1.27-6.11) | 0.011 | Tangkijvanich et al.  2016 |
|  |  | Taiwan | 85 | 30^a^ | PCR | Yes | 3.49 (1.12-10.84) | 0.031 | Tseng et al.  2011 |
|  | rs9277535 | Caucasian | 205 | 57^e^ | PCR | Yes | 2.1 (1.3-3.4) | 0.001 | Brouwer et al.  2014 |
|  |  | Chinese | 47 | 97^e^ | PCR | Yes |  | 0.013 | Cheng et al.  2014 |
| HLA-DQ | rs9276370 | Taiwan | NR | | GWAS | No |  |  | Chang et al.  2014 |
| HLA-DQA1  -DQB1-DRB1 | *0302-*0303-*09 | Chinese | 66 | 152^d^ | PCR | Yes | 1.94 (1.01-3.73) | 0.031 | Zhu et al.  2011 |
| HLA-DQB1 | *0303 |  |  |  |  | Yes | 1.81 (1.07-3.15) | 0.019 |  |
| HLA-DRB1 | *08 |  |  |  |  | Yes | 2.43 (1.02-5.98) | 0.031 |  |
| IFNAR1 | rs2252930 | Chinese | 77 | 169^d^ | PCR | No |  |  | Wu et al.  2009 |
| IFNL4 | rs117648444 | Italy | 126/NR | | PCR | No |  |  | Galmozzi et al.  2018 |
|  | rs368234815 |  |  |  |  | No |  |  |  |
|  | TT-C/ΔG-T  (rs368234815- rs117648444) |  |  |  |  | Yes | 4.30 (1.4-13.4) | 0.01 |  |
|  | ss469415590 | Thai | 149 | 105^g^ | PCR | No |  |  | Limothai et al.  2015 |
| IL-10 | -1082 | Chinese | 28 | 24^b^ | PCR | No |  |  | Wang et al.  2011 |
|  | -819 |  |  |  |  | Yes | 4.4 | 0.01 |  |
|  | -592 |  |  |  |  | Yes | 6.67 | <0.01 |  |
|  | A-T-A ^1^ |  |  |  |  | Yes | 4.38 | 0.02 |  |
| IL-1RN | SNV | Chinese | 70 | 18^b^ | PCR | No |  |  | Chan et al.  2006 |
| IL-1β | -3954 |  |  |  |  | No |  |  |  |
|  | -511 |  |  |  |  | Yes | 10.4(1.1-96.9) | 0.04 |  |
|  | -31 |  |  |  |  | No |  |  |  |
|  | -511/-31 |  |  |  |  | No |  |  |  |
| IL-21 | rs2221903 | Chinese | 97 | 46^a^ | PCR | Yes | 0.31 (0.12-0.79) | 0.014 | Wang et al.  2018 |
|  | rs907715 |  |  |  |  | Yes | 3.23 (1.0-10.4) | 0.039 |  |
| IL-21R | rs3093301 |  |  |  |  | No |  |  |  |
|  | rs2285452 |  |  |  |  |  |  |  |  |
| IL-28B | rs12979860 | Chinese | 138 | 74^a^ | MassArray | Yes | 2.07 (1.00-4.30) | 0.047 | Wu et al.  2015 |
|  |  | Italy | 80 | 21^f^ | PCR | Yes | 3.9 (1.1-13.2) | 0.025 | Lampertico et al.  2013 |
|  |  | European | 115 | 90^c^ | PCR | Yes | 2.89 (1.15-7.80) | 0.024 | Sonneveld et al.  2012 |
|  |  | Italy | 134 | 56^e^ | PCR | Yes | 11.65 (5.90-23.00) | < 0.001 | Boglione et al.  2014 |
|  |  | Italy | 129 | 61^e^ | PCR | Yes | 11.65 (5.90-23.00) | < 0.001 | Boglione et al.  2015 |
|  |  | Chinese | 76 | 68^g^ | PCR | Yes |  | 0.014 | Cheng et al.  2014 |
|  |  | Chinese | 62 | 84^e^ | PCR | Yes | 2.04 (1.05-3.97) | 0.037 | Guo et al.  2013 |
|  |  | Thailand | 62 | 45^g^ | PCR | No |  |  | Limothai et al.  2016 |
|  |  | Thailand | 76 | 50^e^ | PCR | No |  |  | Tangkijvanich et al.  2016 |
|  |  | Poland | 44 | 26^e^ | PCR | Yes | 0.24(0.09–0.90) | 0.026 | Domagalski et al.  2014 |
|  |  | Caucasian | 10 | 42^e^ | PCR | Yes | 3.3( 1.2-9.2) | 0.021 | Domagalski et al.  2016 |
|  |  | Italy | 91 | 82^e^ | PCR | Yes | 11.58 (5.67-23.67) | <0.001 | Cusato et al.  2017 |
|  | 1. C   (rs12980275-rs12979860) | Chinese | 138 | 74^a^ | MassArray | Yes | 2.53 (1.20-5.34) | 0.015 | Wu et al.  2015 |
|  | rs12980275 |  |  |  |  | Yes | 2.14 (1.03-4.43) | 0.038 |  |
|  |  | Caucasian | 10 | 42^e^ | PCR | Yes | 3.7 (1.3-10.1) | 0.014 | Domagalski et al.  2016 |
|  |  | European | 115 | 90^c^ | PCR | Yes | 3.16 (1.26-8.52) | 0.013 | Sonneveld et al.  2012 |
|  |  | Italy,East-Europe,China,Central Africa | 134 | 56^e^ | PCR | Yes | 7.40 (3.89–14.07) | < 0.001 | Boglione et al.  2014 |
|  |  | Italy | 91 | 82^e^ | PCR | Yes | 4.44 (2.59-7.59) | <0.001 | Cusato et al.  2017 |
|  |  | Poland | 44 | 26^e^ | PCR | No |  |  | Domagalski et al.  2014 |
|  | rs8099917 |  |  |  |  |  |  |  |  |
|  |  | Chinese | 138 | 74^a^ | MassArray | Yes | 2.24 (1.05-4.80) | 0.034 | Wu et al.  2015 |
|  |  | Chinese | 76 | 68^g^ | PCR | Yes |  | 0.014 | Cheng et al.  2014 |
|  |  | Italy | 129 | 61^e^ | PCR | Yes | 4.53 (2.14-9.58) | < 0.001 | Boglione et al.  2015 |
|  |  | Italy,East-Europe,China,Central Africa | 134 | 56^e^ | PCR | Yes | 4.53 (2.14–9.58) | < 0.001 | Boglione et al.  2014 |
|  |  | Italy | 91 | 82^e^ | PCR | Yes | 0.25 (0.12-0.53) | <0.001 | Cusato et al.  2017 |
|  |  | Chinese | 62 | 84^e^ | PCR | Yes | 4.23 (1.17-15.3) | 0.027 | Guo et al.  2013 |
| IP-10 | -201 | Thailand | 62 | 45^b^ | PCR | Yes | 4.52 (1.10-18.60) | 0.037 | Limothai et al.  2016 |
| JAK | Promoter | Taiwan | 46 | 36^d^ | PCR | No |  |  | King et a1.  2002 |
| JAK1 | rs17127090 | Chinese | 77 | 169^d^ | PCR | No |  |  | Wu et al.  2009 |
| KIR/HLA-C | 3DS1-Bw4-80l | Chinese | 76 | 43^b^ | PCR | Yes | 19.85 (2.42-163.00) | 0.0008 | Li et al.  2017 |
|  | 2DS1-C2C2 |  |  |  |  | Yes | 9.87 (1.11-87.50) | 0.04 |  |
|  | 2Dl3-C1C1 |  |  |  |  | Yes | 0.39 (0.18-0.85） | 0.02 |  |
|  | 3DS1-Bw4-80II |  |  |  |  | Yes | 14.58 (1.73-123.05) | 0.006 |  |
| KIR | 2DL5 |  |  |  |  | Yes | 2.38 (1.07-5.26) | 0.03 |  |
|  | 3DS1 |  |  |  |  | Yes | 4.64 (2.02-10.67) | 0.0002 |  |
| HLA-B | w6 |  |  |  |  | Yes | 0.33 (0.12-0.96) | 0.04 |  |
| MxA | -123 | Chinese | 67 | 34^a^ | PCR | No |  |  | Kong et al.  2007 |
|  |  | Taiwan | 46 | 36^d^ | PCR | No |  |  | King et a1.  2002 |
|  | -88 | Chinese | 67 | 34^a^ | PCR | Yes | 5.60 (1.19-26.37) | 0.04 | Kong et al.  2007 |
|  |  | Taiwan | 46 | 36^d^ | PCR | No |  |  | King et a1.  2002 |
| NTCP | S267F | Thailand | 62 | 84^g^ | PCR | Yes | 3.25 (1.23- 8.61) | 0.02 | Thanapirom et al.  2018 |
| OAS | G-T-G-A ^2^ | Chinese | 77 | 169^d^ | PCR | Yes | 2.01 (1.13-3.58) | 0.015 | Wu et al.  2009 |
|  | C-C-T-A ^3^ | Chinese | 322 | 41^f^ | PCR | Yes | 2.21 (1.26-3.90) | 0.005 | Ren et al.  2011 |
|  | C-C-C-A ^4^ |  |  |  |  | Yes | 0.51 (0.26-0.99) | 0.042 |  |
|  | A-C-T-A ^5^ |  |  |  |  | Yes | 1.53 (1.01-2.34) | 0.046 |  |
| OAS1 | rs1131476 | Caucasian | 10 | 42^e^ | PCR | No |  |  | Domagalski et al.  2016 |
|  | rs2285934 | Chinese | 322 | 41^f^ | PCR | No |  |  | Ren et al.  2011 |
|  | Exon 03 | Taiwan | 46 | 36^d^ | PCR | No |  |  | King et a1.  2002 |
|  | rs3177979 | Chinese | 77 | 169^d^ | PCR | No |  |  | Wu et al.  2009 |
| OAS2 | exon 02 | Taiwan | 46 | 36^d^ | PCR | No |  |  | King et a1.  2002 |
|  | rs1293747 | Caucasian | 10 | 42^e^ | PCR | No |  |  | Domagalski et al.  2016 |
|  |  | Chinese | 77 | 169^d^ | PCR | No |  |  | Wu et al.  2009 |
|  | rs2072138 |  | 322 | 41^f^ | PCR | No |  |  | Ren et al.  2011 |
| OAS3 | exon 06-1 | Taiwan | 46 | 36^d^ | PCR | No |  |  | King et a1.  2002 |
|  | exon 06-2 |  |  |  |  | No |  |  |  |
|  | exon 12 |  |  |  |  | No |  |  |  |
|  | exon 16-1 |  |  |  |  | No |  |  |  |
|  | exon 16-2 |  |  |  |  | No |  |  |  |
|  | exon 16-3 |  |  |  |  | No |  |  |  |
|  | exon 16-4 |  |  |  |  | No |  |  |  |
|  | exon 8 |  |  |  |  | No |  |  |  |
|  | rs2072136 | Caucasian | 10 | 42^e^ | PCR | No |  |  | Domagalski et al.  2016 |
|  |  | Chinese | 322 | 41^f^ | PCR | Yes |  | 0.018 | Ren et al.  2011 |
|  | rs4767043 | Chinese | 77 | 169^d^ |  | No |  |  | Wu et al.  2009 |
| OASL | rs10849829 | Caucasian | 30 | 22^e^ | PCR | Yes | 0.26 (0.07-0.88) | 0.044 | Domagalski et al.  2016 |
|  |  | Chinese | 77 | 169^d^ | PCR | No |  |  | Wu et al.  2009 |
|  |  | Chinese | 322 | 41^f^ | PCR | No |  |  | Ren et al.  2011 |
| PRELID2 | rs371991 | Europe,  Asia and North America | 388 | 121^b^ | GWAS | Yes |  | 3.44×10^-6^ | Brouwer et al.  2019 |
| PKR | exon 01 | Taiwan | 46 | 36^d^ | PCR | No |  |  | King et a1.  2002 |
| STAT1 | exon 02 |  |  |  |  | No |  |  |  |
| STAT4 | rs7574865 | Chinese | 328 | 138^a^ | MassArray | Yes | 0.34 (0.21-0.56) | 1.30×10^-5^ | Jiang et al.  2016 |
| TRAPPC9 | rs78900671 | Europe,  Asia and North America | 641 | 282^b^ | GWAS | Yes |  | 6.43×10^-7^ | Brouwer et al.  2019 |
| VDBP | rs7041 | Italy | 91 | 82^e^ | PCR | Yes | 2.81 (1.05-7.50) | 0.039 | Cusato et al.  2017 |
| VDR | rs1544410 | Italy | 91 | 82^e^ | PCR | No |  |  | Cusato et al.  2017 |
|  |  | Egypt | 200/NR | | PCR | Yes |  | 0.023 | Mostafa-Hedeab et al.  2018 |
|  |  | Thailand | 80 | 31^c^ | PCR | No |  |  | Thanapirom et al.  2017 |
|  | rs2228570 |  |  |  |  | No |  |  |  |
|  |  | Thailand | 92 (HBeAg positive) | 30^a^ | PCR | No |  |  | Limothai et al.  2017 |
|  |  |  | 123 (HBeAg negative) | 30^e^ | PCR | No |  |  |  |
|  | rs731236 | Italy | 91 | 82^e^ | PCR | No |  |  | Cusato et al.  2017 |
|  |  | Egypt | 200/NR | | PCR | Yes |  | 0.01 | Mostafa-Hedeab et al.  2018 |
|  |  | Thailand | 80 | 31^c^ | PCR | No |  |  | Thanapirom et al.  2017 |
|  | rs757343 |  |  |  |  | No |  |  |  |
|  | rs7975232 | Italy | 91 | 82^e^ | PCR | No |  |  | Cusato et al.  2017 |
|  |  | Thailand | 80 | 31^c^ | PCR | No |  |  | Thanapirom et al.  2017 |
|  | rs11568820 | Italy | 91 | 82^e^ | PCR | Yes | 2.92 (1.34-6.34) | 0.007 | Cusato et al.  2017 |
|  | rs10735810 | Egypt | 200/NR | | PCR | Yes |  | 0.02 | Mostafa-Hedeab et al.  2018 |
|  |  | Italy | 91 | 82^e^ | PCR | No |  |  | Cusato et al.  2017 |
|  | rs7975253 | Egypt | 200/NR | | PCR | No |  |  | Mostafa-Hedeab et al.  2018 |
|  | bAt haplotype ^6^ |  |  |  |  | Yes |  | 0.043 |  |

**Note:**

Responses to IFN-α treatment are categorized as a, HBeAg seroconversion plus HBV DNA negativity; b, HBeAg negativity plus HBV DNA negativity; c, HBeAg seroconversion; d, HBeAg negativity; e, HBV DNA negativity; f, HBsAg negativity; g, other; If the evaluation method is not clearly indicated, the sample size is marked by numbers, and the criterion of NR marking for “not clear”; SNP, single nucleotide polymorphism; CNVs, copy number variations; HBV: hepatitis B virus; OR (95% CI), odds ratio (95% confidence interval); Yes, positive result reported; No, not statistical significance; NR, date not showed; GWAS, genome-wide association study; PCR, polymerase chain reaction-based research methods; MALDI-TOF-MS, Matrix-Assisted Laser Desorption/ Ionization Time of Flight Mass Spectrometry; MA, Meta-Analysis; Population, including race or region..

**Haplotypes**

1. A-T-A, -1082/-819/-592
2. G-T-G-A, rs3177979-rs1293747-rs4767043-rs10849829
3. C-C-T-A, rs2285934-rs2072138-rs2072136-rs10849829
4. C-C-C-A, rs2285934-rs2072138-rs2072136-rs10849829
5. A-C-T-A, rs2285934-rs2072138-rs2072136-rs10849829
6. bAt haplotype, rs1544410-rs7975253-rs731236

**References:**

Boglione, L., Cusato, J., Allegra, S., Esposito, I., Patti, F., Cariti, G., et al. (2014). Role of IL28-B polymorphisms in the treatment of chronic hepatitis B HBeAg-negative patients with peginterferon. *Antiviral Res* 102, 35-43. doi:10.1016/j.antiviral.2013.11.014

Boglione, L., Cusato, J., De Nicolò, A., Cariti, G., Di Perri, G., and D'Avolio, A. (2015). Role of CYP27B1+2838 promoter polymorphism in the treatment of chronic hepatitis B HBeAg negative with PEG-interferon. *J Viral Hepat* 22, 318-327. doi:10.1111/jvh.12288

Brouwer, W. P., Sonneveld, M. J., Tabak, F., Simon, K., Cakaloglu, Y., Akarca, U. S., et al. (2014). Polymorphisms of HLA-DP are associated with response to peginterferon in Caucasian patients with chronic hepatitis B. *Aliment Pharmacol Ther* 40, 811-818. doi:10.1111/apt.12910

Brouwer, W. P., Chan, H. L., Lampertico, P., Hou, J., Tangkijvanich, P., Reesink, H. W., et al. (2019). Genome Wide Association Study Identifies Genetic Variants Associated With Early And Sustained Response To (Peg)Interferon In Chronic Hepatitis B Patients: The GIANT-B Study. *Clin Infect Dis* doi:10.1093/cid/ciz084

Chan, H. L. Y., Tse, A. M. L., Zhang, M. D., Wong, V. W., Chim, A. M., Hui, A. Y., et al. (2006). Genetic polymorphisms of interleukin-1-beta in association with sustained response to anti-viral treatment in chronic hepatitis B in Chinese. *Aliment Pharmacol Ther* 23, 1703-1711. doi:10.1111/j.1365-2036.2006.02948.x

Chang, S. W., Fann, C. S., Su, W. H., Wang, Y. C., Weng, C. C., Yu, C. J., et al. (2014). A genome-wide association study on chronic HBV infection and its clinical progression in male Han-Taiwanese. *PloS One* 9, e99724. doi:10.1371/journal.pone.0099724

Cheng, L., Sun, X., Tan, S., Tan, W., Dan, Y., Zhou, Y., et al. (2014). Effect of HLA-DP and IL28B gene polymorphisms on response to interferon treatment in hepatitis B e-antigen seropositive chronic hepatitis B patients. *Hepatol Res* 44, 1000-1007. doi:10.1111/hepr.12284

Cusato, J., Boglione, L., De Nicolò, A., Imbornone, R., Cardellino, C. S., Ghisetti, V., et al. (2017). Association of vitamin D pathway SNPs and clinical response to interferon in a cohort of HBeAg-negative patients. *Pharmacogenomics* 18, 651-661. doi:10.2217/pgs-2016-0041

Domagalski, K., Pawłowska, M., Zaleśna, A., Pilarczyk, M., Rajewski, P., Halota, W., et al. (2016). Impact of IL28B and OAS gene family polymorphisms on interferon treatment response in Caucasian children chronically infected with hepatitis B virus. *World J Gastroenterol* 22, 9186-9195. doi:10.3748/wjg.v22.i41.9186

Domagalski, K., Pawłowska, M., Zaleśna, A., Tyczyno, M., Skorupa-Kłaput, M., Tretyn, A., et al. (2014). The relationship between IL-28B polymorphisms and the response to peginterferon alfa-2a monotherapy in anti-HBe-positive patients with chronic HBV infection. *Eur J Clin Microbiol Infect Dis* 33, 2025-2033. doi:10.1007/s10096-014-2172-1

Galmozzi, E., Facchetti, F., Grossi, G., Loglio, A., Viganò, M., Lunghi, G., et al. (2018). IFNL4 rs368234815 and rs117648444 variants predict off-treatment HBsAg seroclearance in IFN-treated HBeAg-negative chronic hepatitis B patients. *Liver Int* 38, 417-423. doi:10.1111/liv.13526

Guo, X., Yang, G., Yuan, J., Ruan, P., Zhang, M., Chen, X., et al. (2013). Genetic variation in interleukin 28B and response to antiviral therapy in patients with dual chronic infection with hepatitis B and C viruses. *PLoS One* 8, e77911. doi:10.1371/journal.pone.0077911

Jiang, D. K., Wu, X., Qian, J., Ma, X. P., Yang, J., Li, Z., et al. (2016). Genetic variation in STAT4 predicts response to interferon-α therapy for hepatitis B e antigen-positive chronic hepatitis B. *Hepatology* 63, 1102-1111. doi:10.1002/hep.28423

King, J. K., Yeh, S. H., Lin, M. W., Liu, C. J., Lai, M. Y., Kao, J. H., et al. (2002). Genetic polymorphisms in interferon pathway and response to interferon treatment in hepatitis B patients: A pilot study. *Hepatology* 36, 1416-1424. doi:10.1053/jhep.2002.37198

Kong, X., Zhang, X., Gong, Q., Gao, J., Zhang, S., Wang, L., et al. (2007). MxA induction may predict sustained virologic responses of chronic hepatitis B patients with IFN-alpha treatment. *J Interferon Cytokine Res* 27, 809-818. doi:10.1089/jir.2006.0163

Lampertico, P., Viganò, M., Cheroni, C., Facchetti, F., Invernizzi, F., Valveri, V., et al. (2013). IL28B polymorphisms predict interferon-related hepatitis B surface antigen seroclearance in genotype D hepatitis B e antigen-negative patients with chronic hepatitis B. *Hepatology* 57, 890-896. doi:10.1002/hep.25749

Li, W., Shen, X., Fu, B., Guo, C., Liu, Y., Ye, Y., et al. (2017). KIR3DS1/HLA-B Bw4-80Ile Genotype Is Correlated with the IFN-α Therapy Response in hepatitis B e antigen-Positive Chronic Hepatitis B. *Front Immunol* 8, 1285. doi:10.3389/fimmu.2017.01285

Limothai, U., Chuaypen, N., Khlaiphuengsin, A., Posuwan, N., Wasitthankasem, R., Poovorawan, Y., et al. (2016). Association of interferon-gamma inducible protein 10 polymorphism with treatment response to pegylated interferon in HBeAg-positive chronic hepatitis B. *Antivir Ther* 21, 97-106. doi:10.3851/IMP2992

Limothai, U., Chuaypen, N., Khlaiphuengsin, A., Chittmittraprap, S., Poovorawan, Y., and Tangkijvanich, P. (2017). Association of vitamin-D-related genetic variations and treatment response to pegylated interferon in patients with chronic hepatitis B. *Antivir Ther* 22, 681-688. doi:10.3851/IMP3154

Limothai, U., Wasitthankasem, R., Poovorawan, Y., and Tangkijvanich, P. (2015). Single Nucleotide Polymorphism of Interferon Lambda-4 Gene is not Associated with Treatment Response to Pegylated Interferon in Thai Patients with Chronic Hepatitis B. *Asian Pac J Cancer Prev* 16, 5515-5519. doi:10.7314/APJCP.2015.16.13.5515

Mostafa-Hedeab, G., Sabry, D., Abdelaziz, G. M., Ewaiss, M., Adli, N., and Fathy, W. (2018). Influence of Vitamin D Receptor Gene Polymorphisms on Response to Pegylated Interferon in Chronic Hepatitis B Egyptian Patients. *Rep Biochem Mol Biol* 6, 186-196.

Ren, S., Yu, H., Zhang, H., Liu, Y., Huang, Y., Ma, L., et al. (2011). Polymorphisms of interferon-inducible genes OAS associated with interferon-α treatment response in chronic HBV infection. *Antiviral Res* 89, 232-237. doi:10.1016/j.antiviral.2011.01.006

Sonneveld, M. J., Wong, V. W., Woltman, A. M., Wong, G. L., Cakaloglu, Y., Zeuzem, S., et al. (2012). Polymorphisms Near IL28B and Serologic Response to Peginterferon in HBeAg-Positive Patients With Chronic Hepatitis B. *Gastroenterology* 142, 513-520.e1. doi:10.1053/j.gastro.2011.11.025

Stelma, F., Jansen, L., Sinnige, M. J., van Dort, K. A., Takkenberg, R. B., Janssen, H. L. A., et al. (2016). HLA-C and KIR combined genotype as new response marker for HBeAg-positive chronic hepatitis B patients treated with interferon-based combination therapy. *J Viral Hepat* 23, 652-659. doi:10.1111/jvh.12525

Tangkijvanich, P., Chittmittraprap, S., Poovorawan, K., Limothai, U., Khlaiphuengsin, A., Chuaypen, N., et al. (2016). A randomized clinical trial of peginterferon alpha-2b with or without entecavir in patients with HBeAg-negative chronic hepatitis B: Role of host and viral factors associated with treatment response. *J Viral Hepat* 23, 427-438. doi:10.1111/jvh.12467

Thanapirom, K., Suksawatamnuay, S., Sukeepaisarnjaroen, W., Treeprasertsuk, S., Tanwandee, T., Charatcharoenwitthaya, P., et al. (2018). Association of the S267F variant on NTCP gene and treatment response to pegylated interferon in patients with chronic hepatitis B: a multicentre study. *Antivir Ther* 23, 67-75. doi:10.3851/IMP3179

Thanapirom, K., Suksawatamnuay, S., Sukeepaisarnjareon, W., Tanwandee, T., Charatcharoenwitthaya, P., Thongsawat, S., et al. (2017). Genetic variation in the vitamin D pathway CYP2R1 gene predicts sustained HBeAg seroconversion in chronic hepatitis B patients treated with pegylated interferon: A multicenter study. *PLoS One* 12, e0173263. doi:10.1371/journal.pone.0173263

Tseng, T., Yu, M., Liu, C., Lin, C., Huang, Y., Hsu, C., et al. (2011). Effect of host and viral factors on hepatitis B e antigen-positive chronic hepatitis B patients receiving pegylated interferon-α-2a therapy. *Antivir Ther* 16, 629-637. doi:10.3851/IMP1841

Wang, S., Huang, D., Sun, S., Ma, W., and Zhen, Q. (2011). Interleukin-10 promoter polymorphism predicts initial response of chronic hepatitis B to interferon alfa. *Virol J* 8, 28. doi:10.1186/1743-422X-8-28

Wang, X., Xu, Z., Fu, J., Cheng, L., Li, Y., Li, L., et al. (2018). Role of interleukin-21 and interleukin-21 receptor polymorphisms in the treatment of HBeAg-positive chronic hepatitis B patients with peginterferon. *Medicine (Baltimore)* 97, e10891. doi:10.1097/MD.0000000000010891

Wu, H., Zhao, G., Qian, F., Liu, K., Xie, J., Zhou, H., et al. (2015). Association of IL28B polymorphisms with peginterferon treatment response in Chinese Han patients with HBeAg‐positive chronic hepatitis B. *Liver Int* 35, 473-481. doi:10.1111/liv.12491

Wu, X., Xin, Z., Zhu, X., Pan, L., Li, Z., Li, H., et al. (2012). Polymorphisms in ADAR1 gene affect response to interferon alpha based therapy for chronic hepatitis B in Han Chinese. *Antiviral Res* 94, 272-275. doi:10.1016/j.antiviral.2012.03.004

Wu, X., Zhu, X., Zhu, S., Li, J., Ma, J., Li, Z., et al. (2009). A pharmacogenetic study of polymorphisms in interferon pathway genes and response to interferon-alpha treatment in chronic hepatitis B patients. *Antiviral Res* 83, 252-256. doi:10.1016/j.antiviral.2009.06.003

Wu, Y., Zeng, Y., Wu, W., Lin, J., and Ou, Q. (2018). Polymorphisms of CYP27B1 are associated with IFN efficacy in HBeAg-positive patients. *J Clin Lab Anal* 32, e22367. doi:10.1002/jcla.22367

Zhang, T., Zhang, Z., Gao, Y., Zhang, Y., Yang, D., and Li, X. (2010). T29C genotype polymorphism of estrogen receptor alpha is associated with initial response to interferon-alpha therapy in chronic hepatitis B patients. *Hepatobiliary Pancreat Dis Int* 9, 275-279.

Zhang, T., Zhang, Z., Zhang, Y., Ye, J., and Li, X. (2016). A Lower PBMC Estrogen Receptor α Gene Expression in Chronic Hepatitis B Is Associated with a Sustained Virological Response to Pegylated Interferon. *J Interferon Cytokine Res* 36, 120-128. doi:10.1089/jir.2014.0223

Zhu, X., Du, T., Wu, X., Guo, X., Niu, N., Pan, L., et al. (2011). Human leukocyte antigen class I and class II genes polymorphisms might be associated with interferon α therapy efficiency of chronic hepatitis B. *Antiviral Res* 89, 189-192. doi:10.1016/j.antiviral.2011.01.001
