# Supplementary material for: Host Genetic Determinants of Hepatitis B Virus Infection
Source: Front Genet. 2019 Aug 13;10:696. doi: 10.3389/fgene.2019.00696 (PMC6702792; doi:10.3389/fgene.2019.00696)
Supplement: Supplementary file 6 [file Table_6.doc]

**Supplement Table S6. Selected studies on host genetic factors associated with response to NUC therapy of patients with chronic hepatitis B.**

| **Genes** | **Gentic determinants**  **SNP/Hap/CNVs** | **Population**  **/Region** | **Non-responder (n)** | **Responder (n)** | **Methods** | **Disease association** | **OR (95% CI)** | **P value** | **Reference** |
| --- | --- | --- | --- | --- | --- | --- | --- | --- | --- |
| CD40 | rs1883832 | Chinese | 76/NR | | PCR | Yes | 3.93 (1.21-12.76) | 0.023 | Li et al.  2018 |
| CFB | rs12614 | Chinese | 76/NR | | PCR | No |  |  | Li et al.  2018 |
| CTLA-4 | rs231775 | Taiwan | 100/NR | | PCR | Yes | 1.74 (1.01-3.00) | 0.048 | Su et al.  2018 |
| ESR1 | Pvu II | Chinese | 7 | 45^e^ | PCR | Yes |  | 0.018 | Zhang et al.  2013 |
|  | Xba I |  |  |  |  | No |  |  |  |
| HLA-DP | rs3077 | Japanese | 184 | 18^f^ | PCR | Yes | 2.69 (1.04-6.93) | 0.041 | Hosaka et al.  2015 |
|  |  | Taiwan | 100/NR | | PCR | Yes | 10.84 (1.12-105) | 0.04 | Su et al.  2018 |
|  | rs9277535 |  |  |  |  | No |  |  |  |
|  |  | Chinese | 76/NR | | PCR | No |  |  | Li et al.  2018 |
|  |  | Japanese | 184 | 18^f^ | PCR | Yes | 2.78 (1.04-7.42) | 0.041 | Hosaka et al.  2015 |
|  | A-A (rs3077-rs9277535) |  |  |  |  | Yes | 2.81 (1.09-7.25) | 0.033 |  |
| HLA‑DQ | rs2856718 | Chinese | 76/NR | | PCR | No |  |  | Li et al.  2018 |
|  |  | Chinese | NR | | PCR | No |  |  | Zhang et al.  2014 |
|  | rs7453920 | Chinese | 76/NR | | PCR | No |  |  | Li et al.  2018 |
|  | rs9275572 | Chinese | NR | | PCR | Yes | 2.60 (1.53-4.43) | 4.43×10^-4^ | Zhang et al.  2014 |
|  | rs9276370 (LAM) | Taiwan | 28 | 91^e^ | GWAS | Yes | 5.41 (1.73-16.95) | 0.0037 | Chang et al.  2014 |
|  | rs9276370 (ETV) |  | 17 | 47^e^ |  | No |  |  |  |
| HLA-DRB1 | *010101 | Korea | 26 | 308^e^ | PCR | Yes | 0.19 (0.06-0.57) | 0.025 | Jin et al.  2011 |
|  | *070101 | Korea | 26 | 308^e^ | PCR | No |  |  |  |
|  | *080302 |  |  |  |  | No |  |  |  |
|  | *090102 |  |  |  |  | No |  |  |  |
|  | *120201 |  |  |  |  | No |  |  |  |
|  | *130201 |  |  |  |  | No |  |  |  |
|  | *140101 |  |  |  |  | No |  |  |  |
|  | *150101 |  |  |  |  | No |  |  |  |
| IL-10 | rs1800896 | Poland | 26 | 28^e^ | PCR | No |  |  | Stalke et al.  2014 |
|  | rs1800871 |  |  |  |  | No |  |  |  |
|  | rs1800872 |  |  |  |  | No |  |  |  |
|  | A-T-A ^1^ |  |  |  |  | No |  |  |  |
| IL-28B | rs8099917 | Japanese | 184 | 18^f^ | PCR | No |  |  | Hosaka et al.  2015 |
| KIR | 2DL3 | Chinese | 139 | 59^b^ | PCR | Yes | 3.63 (10.83-1.21) | 0.019 | Zhuang et al.  2018 |
|  | 2DS3 |  |  |  |  | Yes | 0.40 (0.83-0.20) | 0.018 |  |
|  | 3DS1 |  |  |  |  | Yes | 1.95 (3.65-1.04) | 0.041 |  |
|  | 2DL1 |  |  |  |  | No |  |  |  |
|  | 2DL2 |  |  |  |  | No |  |  |  |
|  | 2DL4 |  |  |  |  | No |  |  |  |
|  | 2DL5 |  |  |  |  | No |  |  |  |
|  | 3DL1 |  |  |  |  | No |  |  |  |
|  | 3DL2 |  |  |  |  | No |  |  |  |
|  | 3DL3 |  |  |  |  | No |  |  |  |
|  | 2DS1 |  |  |  |  | No |  |  |  |
|  | 2DS2 |  |  |  |  | No |  |  |  |
|  | 2DS4 |  |  |  |  | No |  |  |  |
|  | 2DS5 |  |  |  |  | No |  |  |  |
|  | 2DP1 |  |  |  |  | No |  |  |  |
|  | 3DP1 |  |  |  |  | No |  |  |  |
| NOTCH4 | rs422951 | Chinese | 76/NR | | PCR | No |  |  | Li et al.  2018 |
| NTCP | rs2296651 | Taiwan | 100/NR | | PCR | No |  |  | Su et al.  2018 |

**Note:**

Responses to NUC therapy are categorized as a, HBeAg seroconversion plus HBV DNA negativity; b, HBeAg negativity plus HBV DNA negativity; c, HBeAg seroconversion; d, HBeAg negativity; e, HBV DNA negativity; f,HBsAg negativity; g,other; If the evaluation method is not clearly indicated, the sample size is marked by numbers, and the criterion of NR marking for “not clear”; SNP, single nucleotide polymorphism; HBV, hepatitis B virus; OR (95% CI), odds ratio (95% confidence interval); Yes, Positive result reported; No, not statistical significance; NR,date not showed; GWAS, genome-wide association study; PCR, polymerase chain reaction-based research methods; Population, including race or region.

**Haplotype**

1. A-T-A, rs1800896-rs1800871-rs1800872

**References:**

Chang, S. W., Fann, C. S., Su, W. H., Wang, Y. C., Weng, C. C., Yu, C. J. et al. (2014). A genome-wide association study on chronic HBV infection and its clinical progression in male Han-Taiwanese. *PLoS One* 9, e99724. doi:10.1371/journal.pone.0099724.

Hosaka, T., Suzuki, F., Kobayashi, M., Fukushima, T., Kawamura, Y., Sezaki, H. et al. (2015). HLA-DP genes polymorphisms associate with hepatitis B surface antigen kinetics and seroclearance during nucleot(s)ide analogue therapy. *Liver Int* 35, 1290-302. doi:10.1111/liv.12652.

Jin, Y. J., Shim, J. H., Chung, Y. H., Kim, J. A., Choi, J. G., Park, W. H. et al. (2011). HLA-DRB1*010101 allele is closely associated with poor virological response to lamivudine therapy in patients with chronic hepatitis B. *Digestion* 84 Suppl 1, 35-42. doi:10.1159/000333783.

Li, T., Liu, F., Zhang, L., Ye, Q., Fan, X., Xue, Y. et al. (2018). Host genetic factors in predicting response status in chronic hepatitis B patients discontinuing nucleos(t)ide analogs. *Saudi J Gastroenterol* 24, 30-36. doi:10.4103/sjg.SJG_228_17.

Stalke, P., Rybicka, M., Wroblewska, A., Dreczewski, M., Stracewska, E., Smiatacz, T. et al. (2014). An initial assessment of correlations between host- and virus-related factors affecting analogues antiviral therapy in HBV chronically infected patients. *Med Sci Monit* 20, 321-8. doi:10.12659/MSM.889788.

Su, T. H., Yang, H. C., Tseng, T. C., Liou, J. M., Liu, C. H., Chen, C. L. et al. (2018). Distinct Relapse Rates and Risk Predictors After Discontinuing Tenofovir and Entecavir Therapy. *J Infect Dis* 217, 1193-1201. doi:10.1093/infdis/jix690.

Zhang, T. T., Ye, J., Xia, S. L., Zhang, Y. F., Su, Q., Zhang, Z. H. et al. (2013). Polymorphism of estrogen receptor alpha (ESR1) is associated with virological response to entecavir (ETV) in nucleoside-naive adult patients with chronic hepatitis B. *Infection* 41, 371-8. doi:10.1007/s15010-012-0320-z.

Zhang, X., Jia, J., Dong, J., Yu, F., Ma, N., Li, M. et al. (2014). HLA-DQ polymorphisms with HBV infection: different outcomes upon infection and prognosis to lamivudine therapy. *J Viral Hepat* 21, 491-8. doi:10.1111/jvh.12159.

Zhuang, Y., Li, X., Xu, H., Ye, H., Sun, D., Liu, X. et al. (2018). Association between KIR Genes and Efficacy of Treatment of HBeAg-Positive Chronic Hepatitis B Patients with Entecavir. *Iran J Immunol* 15, 112.
