# Supplementary material for: Host Genetic Determinants of Hepatitis B Virus Infection
Source: Front Genet. 2019 Aug 13;10:696. doi: 10.3389/fgene.2019.00696 (PMC6702792; doi:10.3389/fgene.2019.00696)
Supplement: Supplementary file 7 [file Table_7.doc]

**Supplement Table S7. Selected studies on host genetic factors associated with response to hepatitis B vaccine.**

| **Genes** | **Gentic determinants**  **SNP/Hap/CNVs** | **Population**  **/Region** | **Case^a^**  **(n)** | **Control^b^**  **(n)** | **Research methods** | **Association with response** | **OR (95% CI)** | **P value** | **Reference** |
| --- | --- | --- | --- | --- | --- | --- | --- | --- | --- |
| BTNL2 | rs3763316 | Chinese | 498 | 1449 | GWAS | Yes | 1.84 (1.56-2.17) | 3.75×10^-13^ | Pan et al.  2014 |
|  |  | Chinese | 566 | 1040 | PCR | Yes | 1.58 (1.32-1.88) | 2.00×10^-7^ | Yang et al.  2014 |
|  | rs3763311 |  |  |  |  | Yes | 1.27(1.09-1.48) | 0.002 |  |
|  | rs9268494 |  |  |  |  | Yes | 1.41 (1.22-1.64) | 2.90×10^-6^ |  |
|  | rs3806156 |  |  |  |  | Yes | 1.20 (1.05-1.39) | 0.015 |  |
|  | rs2076530 |  |  |  |  | Yes | 1.18 (1.01-1.37) | 0.029 |  |
|  | rs9268501 |  |  |  |  | Yes | 0.81 (0.70-0.95) | 0007 |  |
|  | rs3763313 |  |  |  |  | Yes | 0.74 (0.59-0.91) | 0.004 |  |
|  | G-T-A-T-C-A-G ^1^ |  |  |  |  | Yes | 1.48 (1.02-2.15) | 0.038 |  |
|  | G-T-A-T-C ^2^ |  |  |  |  | Yes | 2.34 (1.64-3.33) | < 1.0×10^-4^ |  |
|  | A-A (rs3806156-rs2076530) |  |  |  |  | Yes | 4.08 (3.12-5.33) | < 1.0×10^-4^ |  |
|  | C-G (rs3806156-rs2076530) |  |  |  |  | Yes | 4.75 (3.54-6.36) | < 1.0×10^-4^ |  |
|  | G-C-A-T-C ^3^ |  |  |  |  | Yes | 0.54 (0.40-0.76) | 1.00×10^-4^ |  |
|  | rs3817963 | Indonesian | 667 | 979 | GWAS | Yes | 1.52 (1.28-1.80) | 8.5×10^-7^ | Davila et al.  2010 |
|  | rs4248166 | Japan | 1193 |  | GWAS | Yes |  | 5.51×10^-12^ | Nishida et al.  2018 |
| CCR2 | 190 | Poland | 185 |  | PCR | No |  |  | Ganczak et al.  2017 |
| CCR5 | Δ32 |  |  |  |  | No |  |  |  |
| FCN2 | –986 | Kenya | 42 | 251 | PCR | No |  |  | Osthoff et al.  2014 |
|  | –602 |  |  |  |  | No |  |  |  |
|  | –557 |  |  |  |  | No |  |  |  |
|  | –4 |  |  |  |  | No |  |  |  |
|  | + 6359 |  |  |  |  | No |  |  |  |
|  | + 6424 |  |  |  |  | No |  |  |  |
| FOXP1 | rs6789153 | Indonesian | 665 | 981 | GWAS | Yes | 1.38 (1.2-1.6) | 9.2×10^-6^ | Davila et al.  2010 |
| GC | rs7041 | Poland | 223 | 692 | PCR | No |  |  | Grzegorzewska et al.  2014 |
|  | rs1155563 |  |  |  |  | No |  |  |  |
|  | rs2298849 |  |  |  |  | No |  |  |  |
| HLA-III | rs9267665 | Indonesian | 3614 |  | GWAS | Yes | 2.05 (1.64-2.57) | 1.24×10^-17^ | Png et al.  2011 |
| HLA-A | *02 | Korean | 944 |  | PCR | Yes | 0.54 (0.33-0.88) | 0.012 | Yoon et al.  2014 |
|  | *33 |  |  |  |  | Yes | 1.64 (1.01-2.67) | 0.044 |  |
| HLA-B | *62 |  |  |  |  | Yes | 2.30 (1.39-3.81) | 0.001 |  |
|  |  | Korean | 290 |  | PCR | Yes | 0.4 (0.2-0.8) | 0.002 | Roh et al.  2016 |
| HLA-DP | rs7770370 | Taiwan | 2057 |  | GWAS | Yes | 0.33 (0.23-0.49) | 1.20×10^−8^ | Wu et al.  2015 |
|  |  | Korean | 290 |  | PCR | Yes | 1.8 (1.2-2.7) | 0.004 | Roh et al.  2016 |
|  | rs9277535 | Indonesian | 3614 |  | GWAS | Yes | 0.72 (0.63-0.81) | 2.91×10^-12^ | Png et al.  2011 |
|  |  | Japanese | 278 |  | PCR | Yes | 0.31 (0.11-0.74) | 0.004 | Okada et al.  2017 |
|  |  | Taiwan | 2057 |  | GWAS | Yes | 0.41 (0.27-0.61) | 7.85×10^−6^ | Wu et al.  2015 |
|  |  | Korean | 290 |  | PCR | Yes | 1.8 (1.2-2.7) | 0.006 | Roh et al.  2016 |
|  | rs3077 | Japanese | 278 |  | PCR | Yes | 0.32 (0.11-0.80) | 0.010 | Okada et al.  2017 |
|  |  | Korean | 290 |  | PCR | No |  |  | Roh et al.  2016 |
|  | rs3830066 |  |  |  |  | No |  |  |  |
|  |  | Taiwan | 2057 |  | GWAS | Yes | 0.25 (0.15-0.44) | 3.43×10^−7^ | Wu et al.  2015 |
|  | rs7770501 | Korean | 290 |  | PCR | Yes | 2.0 (1.3-3.0) | 0.001 | Roh et al.  2016 |
|  | rs3128961 |  |  |  |  | Yes | 1.8 (1.2-2.7) | 0.004 |  |
|  |  | Taiwan | 2057 |  | GWAS | Yes | 0.43 (0.28-0.65) | 3.88×10^-5^ | Wu et al.  2015 |
|  | rs2116260 |  |  |  |  | Yes | 0.36 (0.22-0.58) | 2.48×10^-5^ |  |
|  | rs5025825 |  |  |  |  | Yes | 0.15 (0.066-0.36) | 1.77×10^-6^ |  |
|  | rs6457709 |  |  |  |  | Yes | 0.21 (0.098-0.44) | 8.83×10^-6^ |  |
|  | rs35953215 |  |  |  |  | Yes | 0.25 (0.15-0.44) | 3.43×10^-7^ |  |
|  | rs9277542 |  |  |  |  | Yes | 0.39 (0.25-0.60) | 1.00×10^-5^ |  |
|  | rs4282438 |  |  |  |  | Yes | 0.48 (0.34-0.68) | 3.57×10^-5^ |  |
|  | 04:02 | Japanese | 1193 |  | GWAS | Yes |  | 7.58×10^-8^ | Nishida et al.  2018 |
|  |  | Japanese | 312 | 836 | PCR | Yes | 0.35 (0.19-0.61) | 1.0×10^-4^ | Sakai et al.  2017 |
|  | 05:01 | Japanese | 1193 |  | GWAS | Yes |  | 4.76×10^-9^ | Nishida et al.  2018 |
|  |  | Japanese | 312 | 836 | PCR | Yes | 1.86 (1.43-2.42) | <1.0×10^-4^ | Sakai et al. 2017 |
|  |  | Taiwan | 342 | 1020 | PCR | Yes | 1.98 (1.53-2.56) | 1.9×10^-8^ | Wu et al.  2013 |
|  | 09:01 |  |  |  |  | Yes | 3.91 (1.45-10.58) | 0.017 |  |
|  | 02:02 |  |  |  |  | Yes | 0.13 (0.03-0.54) | 9.1×10^-4^ |  |
|  | 03:01:01 |  |  |  |  | Yes | 0.29 (0.12-0.67) | 0.0041 |  |
|  | 04:01:01 |  |  |  |  | Yes | 0.28 (0.11-0.70) | 0.0093 |  |
| HLA-DP | A-G-A-G-G ^4^ | Taiwan | 2057 |  | GWAS | Yes | 2.72 (1.85-4.02) | 4.48×10^-7^ | Wu et al.  2015 |
|  | A-G (rs3128961-rs9277535) |  |  |  |  | Yes | 2.26 (1.53-3.35) | 4.65×10^-5^ |  |
|  | A-G-A-G-G-A-G ^5^ |  |  |  |  | Yes | 2.51 (1.74-3.61) | 8.28×10^-7^ |  |
| HLA-DQ | rs2856718 | Japanese | 278 |  | PCR | No |  |  | Okada et al.  2017 |
|  | rs7453920 |  |  |  |  | No |  |  |  |
| HLA-DQB1 | *02 |  | 2308 |  | MA | Yes | 0.28 ( 0.18-0.42) | <1.0×10^-5^ | Li et al.  2013 |
|  | *0201 | Chinese | 297 |  | PCR | No |  |  | Xu et al.  2017 |
|  | *0401 |  |  |  |  | Yes | 2.17 (1.01-4.73) | 0.047 |  |
|  |  | Japanese | 1193 |  | GWAS | Yes |  | 5.01×10^-9^ | Nishida et al.  2018 |
|  | *05 |  | 2308 |  | MA | Yes | 1.85 (1.17-2.93) | 0.008 | Li et al.  2013 |
|  | *0501 |  |  |  |  | Yes | 2.35 (1.09-5.05) | 0.03 |  |
|  |  | Chinese | 297 |  | PCR | No |  |  | Xu et al.  2017 |
|  |  | Japanese | 312 | 836 | PCR | Yes | 0.16 (0.06-0.46) | <0.0001 | Sakai et al.  2017 |
|  |  | Japanese | 1193 |  | GWAS | Yes |  | 4.47×10^-5^ | Nishida et al. 2018 |
|  | *06 |  | 2308 |  | MA | Yes | 2.34 (1.09-5.01) | 0.03 | Li et al.  2013 |
|  | *0602 |  |  |  |  | Yes | 3.32 (1.80-6.15) | 1.0×10^-4^ |  |
|  |  | Japanese | 1193 |  | GWAS | Yes |  | 3.23×10^-4^ | Nishida et al. 2018 |
| HLA-DR | rs3135363 | Indonesian | 3614 |  | GWAS | Yes | 1.53 (1.35-1.74) | 6.53×10^-22^ | Png et al.  2011 |
| HLA-DRA | rs5000563 | Indonesian | 667 | 979 | GWAS | Yes | 0.61 (0.52-0.71) | 5.57×10^-10^ | Davila et al. 2010 |
|  | rs2395177 |  |  |  |  | Yes | 0.61 (0.52-0.71) | 6.4×10^-10^ |  |
|  | rs7192 |  |  |  |  | Yes | 1.43 (1.24-1.65) | 1.1×10^-6^ |  |
| HLA-DRB1 | *01 | Chinese | 297 |  | PCR | No |  |  | Xu et al.  2017 |
|  |  |  | 2308 |  | MA | Yes | 2.73 (1.55-4.81) | 5.0×10^-4^ | Li et al.  2013 |
|  | *01:01 | Japanese | 312 | 836 | PCR | Yes | 0.18 (0.07-0.50) | 1.0×10^-4^ | Sakai et al. 2017 |
|  |  | Japanese | 1193 |  | GWAS | Yes |  | 8.89×10^-5^ | Nishida et al. 2018 |
|  | *03 | Chinese | 297 |  | PCR | No |  |  | Xu et al.  2017 |
|  |  |  | 2308 |  | MA | Yes | 0.55 (0.35-0.86) | 0.009 | Li et al.  2013 |
|  | *0301 |  |  |  |  | Yes | 0.42 (0.21-0.84) | 0.01 |  |
|  | *04 | Chinese | 297 |  | PCR | No |  |  | Xu et al.  2017 |
|  |  | Korean | 944 |  | PCR | Yes | 1.88 (1.18-3.02) | 0.008 | Yoon et al. 2014 |
|  |  |  | 2308 |  | MA | Yes | 0.57 (0.37-0.87) | 0.009 | Li et al.  2013 |
|  | *04:05 | Japanese | 1193 |  | GWAS | Yes |  | 2.61×10^-9^ | Nishida et al. 2018 |
|  | *07 | Korean | 944 |  | PCR | Yes | 2.19 (1.23-3.90) | 0.007 | Yoon et al. 2014 |
|  |  | Chinese | 297 |  | PCR | No |  |  | Xu et al.  2017 |
|  |  | Korea | 290 |  | PCR | No |  |  | Roh et al.  2016 |
|  |  |  | 2308 |  | MA | Yes | 0.24 (0.14-0.43) | <1.0×10^-5^ | Li et al.  2013 |
|  | *08 | Chinese | 297 |  | PCR | No |  |  | Xu et al.  2017 |
|  |  | Korea | 290 |  | PCR | Yes | 4.8 (1.1-20.4) | 0.021 | Roh et al.  2016 |
|  |  | Korean | 944 |  | PCR | Yes | 0.18 (0.06-0.57) | 0.001 | Yoon et al. 2014 |
|  | *08:03 | Japanese | 1193 |  | GWAS | Yes |  | 2.75×10^-6^ | Nishida et al. 2018 |
|  |  | Japanese | 312 | 836 | PCR | Yes | 0.31 (0.15-0.62) | 3.0×10^-4^ | Sakai et al. 2017 |
|  | *11 | Chinese | 297 |  | PCR | No |  |  | Xu et al.  2017 |
|  | *1301 |  |  |  |  | No |  |  |  |
|  |  |  | 2308 |  | MA | Yes | 5.94 (2.65-13.32) | <1.0×10^-4^ | Li et al.  2013 |
|  | *1302 | Chinese | 297 |  | PCR | No |  |  | Xu et al.  2017 |
|  |  |  | 2308 |  | MA | Yes | 0.25 (0.09-0.68) | 0.007 | Li et al.  2013 |
|  | *15 |  |  |  |  | Yes | 2.29 (1.61-3.27) | <1.0×10^-5^ |  |
|  | *15:01 | Japanese | 1193 |  | GWAS | Yes |  | 2.5×10^-4^ | Nishida et al. 2018 |
|  | rs477515 | Chinese | 498 | 1449 | GWAS | Yes | 2.05 (1.75-2.41) | 2.63×10^-19^ | Pan et al.  2014 |
|  | rs28366298 |  |  |  |  | Yes | 1.77 (1.53-2.05) | 1.67×10^-14^ |  |
|  | rs13204672 |  |  |  |  | Yes | 2.01 (1.67-2.43) | 1.45×10^-13^ |  |
| HLA-DRB1-DQB1 | *01:01-*05:01 | Japanese | 1193 |  | GWAS | Yes |  | 8.89×10^-5^ | Nishida et al. 2018 |
|  | *04:05-*04:01 |  |  |  |  | Yes |  | 2.61×10^-9^ |  |
|  | *08:03-*06:01 |  |  |  |  | Yes |  | 2.75×10^-6^ |  |
|  | *14:06-*03:01 |  |  |  |  | Yes |  | 6.4×10^-4^ |  |
|  | *15:01-*06:02 |  |  |  |  | Yes |  | 3.2×10^-4^ |  |
| IL-1β | rs1143627 | Chinese | 215 | 86 | PCR | No |  |  | Wang et al. 2012 |
|  | rs16944 |  |  |  |  | No |  |  |  |
|  | rs1143634 |  |  |  |  | No |  |  |  |
|  | A-G (rs1143633-rs1143627) | Chinese | 24 | 46 | MassARRAY | Yes |  | 0.035 | Chen et al. 2011 |
| IL-2 | rs2069762 | Chinese | 215 | 86 | PCR | No |  |  | Wang et al. 2012 |
|  | rs2069763 |  |  |  |  | No |  |  |  |
| IL-4 | rs2070874 |  | 1030 | 843 | MA | Yes | 1.53 (1.13-2.06) | 0.006 | Cui et al.  2013 |
|  |  | Chinese | 215 | 86 | PCR | Yes | 1.92 (1.18-3.12) | 0.008 | Wang et al. 2012 |
|  | rs2243250 |  | 1030 | 843 | MA | Yes | 1.39 (1.10-1.76) | 0.006 | Cui et al.  2013 |
|  | rs2243250 | Chinese | 215 | 86 | PCR | Yes | 1.82 (1.12-2.96) | 0.015 | Wang et al. 2012 |
|  |  | Chinese | 107 | 214 | PCR | No |  |  | Pan et al.  2012 |
|  | rs2227282 |  |  |  |  | No |  |  |  |
|  |  |  | 1030 | 843 | MA | No |  |  | Cui et al.  2013 |
|  | rs2243248 | Chinese | 24 | 46 | MassARRAY | Yes | 5.37 (1.00-28.83) | 0.031 | Chen et al. 2011 |
|  |  |  | 1030 | 843 | MA | No |  |  | Cui et al.  2013 |
|  | rs2227284 |  |  |  |  | Yes | 1.80 (1.03-3.16) | 0.040 |  |
| IL-4RA | rs1805015 | Chinese | 24 | 46 | MassARRAY | Yes | 0.13 (0.02-1.07) | 0.028 | Chen et al. 2011 |
| IL-10 | rs1800872 | Chinese | 107 | 214 | PCR | No |  |  | Pan et al.  2012 |
|  | rs1800872 | Chinese | 215 | 86 | PCR | No |  |  | Wang et al. 2012 |
|  | rs1800896 |  |  |  |  | No |  |  |  |
|  | A-C-C ^6^ | Germany | 202 twin pairs |  | PCR | Yes |  | <1.0×10^-4^ | Hohler et al. 2005 |
| IL-12A | rs2243115 | Chinese | 107 | 214 | PCR | No |  |  | Pan et al.  2012 |
| IL-12B | rs17860508 | Chinese | 107 | 214 | PCR | Yes | 1.41 (1.00-1.99) | 0.039 | Pan et al.  2012 |
|  | rs3213094 | Chinese | 107 | 214 | PCR | No |  |  | Pan et al.  2012 |
|  | rs3212227 | Chinese | 215 | 86 | PCR | No |  |  | Wang et al. 2012 |
| IL-13 | rs1295686 | Chinese | 215 | 86 | PCR | No |  |  | Wang et al. 2012 |
|  | rs1295686 | Chinese | 24 | 46 | MassARRAY | Yes | 2.69 (1.28-5.66) | 0.008 | Chen et al. 2011 |
| TNF | rs1800629 | Chinese | 215 | 86 | PCR | No |  |  | Wang et al. 2012 |
| IL12A / IL12B | rs2243115/rs17860508  (TT -CTCTAA/CTCTAA) | Chinese | 107 | 214 | PCR | Yes | 2.19 (1.23-3.93) | 0.008 | Pan et al.  2012 |
| IFN-γ | rs2069727 | Chinese | 107 | 214 | PCR | No |  |  | Pan et al.  2012 |
|  | rs2069705 |  |  |  |  | No |  |  |  |
| IL-17 | rs4711998 | Iran | 21 | 99 | PCR | Yes | 0.34 (0.13-0.89) | 0.025 | Borzooy et al. 2016 |
| IL-22 | rs1026780 | Iran | 21 | 99 | PCR | No |  |  | Borzooy et al. 2016 |
|  | rs2227501 |  |  |  |  | No |  |  |  |
|  | rs2227503 |  |  |  |  | No |  |  |  |
| LILRB4 | rs1654668 | Indonesian | 665 | 981 | GWAS | Yes | 1.34 (1.16-1.56) | 8×10^–5^ | Davila et al. 2010 |
| MBL2 | Codon 57 | Kenya | 42 | 251 | PCR | No |  |  | Osthoff et al. 2014 |
|  | Codon 54 |  |  |  |  | No |  |  |  |
|  | Codon 52 |  |  |  |  | No |  |  |  |
|  | promoter |  |  |  |  | No |  |  |  |
| RXRA | rs10881578 | Poland | 223 | 692 | PCR | No |  |  | Grzegorzewska et al.  2014 |
|  | rs10776909 |  |  |  |  | No |  |  |  |
|  | rs749759 |  |  |  |  | No |  |  |  |
| TLR-2 | rs3804100 | Chinese | 24 | 46 | MassARRAY | Yes | 0.28 (0.09-0.81) | 0.032 | Chen et al. 2011 |
|  |  | Chinese | 215 | 86 | PCR | No |  |  | Wang et al. 2012 |
| TNFRSF1A | rs4149623 | Chinese | 107 | 214 | PCR | No |  |  | Pan et al.  2012 |
|  | rs767455 |  |  |  |  | No |  |  |  |
| VDR | rs2228570 | Poland | 223 | 692 | PCR | No |  |  | Grzegorzewska et al.  2014 |
|  | rs1544410 |  |  |  |  | Yes | 1.49 (1.03-2.17) | 0.036 |  |
| SC | CNVs | Chinese | 387 |  | AccuCopy assay | No |  |  | Xu et al.  2018 |
| ITGAL |  |  |  |  |  | No |  |  |  |
| CD58 |  |  |  |  |  | No |  |  |  |
| TNFSF15 |  |  |  |  |  | No |  |  |  |
| CCL15 |  |  |  |  |  | No |  |  |  |
| TGFB3 |  |  |  |  |  | No |  |  |  |
| BCL6 |  |  |  |  |  | No |  |  |  |

**Note:**

1. Case:numbers of non- or low- responders to HBV vaccine; b. Control: numbers of responders or high responders to HBV vaccine; In a minority of the studies, the numbers of subjects of each groups were not separately given. For these studies, the total number of all subjects included were given in the case column. SNP, single nucleotide polymorphism; CNVs, copy number variations; HBV, hepatitis B virus; OR (95%CI), odds ratio (95% confidence interval); Yes, positive result reported; No, not statistical significance; GWAS, genome-wide association study; PCR, polymerase chain reaction-based research methods; MALDI-TOF-MS, Matrix-Assisted Laser Desorption/ Ionization Time of Flight Mass Spectrometry; MA, Meta-Analysis; Population, including race or region.

**Haplotypes**

1. G-T-A-T-C-A-G, rs9268501-rs3763316- rs3763313-rs3763311- rs9268494- rs3806156-rs2076530
2. G-T-A-T-C, rs9268501-rs3763316- rs3763313-rs3763311-rs9268494
3. G-C-A-T-C, rs9268501-rs3763316- rs3763313-rs3763311-rs9268494
4. A-G-A-G-G, rs5025825-rs6457709-rs35953215-rs3830066-rs7770370
5. A-G-A-G-G-A-G, rs5025825-rs6457709-rs35953215-rs3830066-rs7770370-rs3128961-rs9277535
6. A-C-C, -1082/-819/-592

**References:**

Borzooy, Z., Streinu-Cercel, A., Mirshafiey, A., Khamseh, A., Mahmoudie, M. K., Navabi, S. S. et al. (2016). IL-17 and IL-22 genetic polymorphisms in HBV vaccine non- and low-responders among healthcare workers. *Germs* 6, 14-20. doi:10.11599/germs.2016.1084.

Chen, J., Liang, Z., Lu, F., Fang, X., Liu, S., Zeng, Y. et al. (2011). Toll-like receptors and cytokines/cytokine receptors polymorphisms associate with non-response to hepatitis B vaccine. *Vaccine* 29, 706-11. doi:10.1016/j.vaccine.2010.11.023.

Cui, W., Sun, C. M., Deng, B. C. and Liu, P. (2013). Association of polymorphisms in the interleukin-4 gene with response to hepatitis B vaccine and susceptibility to hepatitis B virus infection: a meta-analysis. *Gene* 525, 35-40. doi:10.1016/j.gene.2013.04.065.

Davila, S., Froeling, F. E. M., Tan, A., Bonnard, C., Boland, G. J., Snippe, H. et al. (2010). New genetic associations detected in a host response study to hepatitis B vaccine. *Genes and Immunity* 11, 232 - 238. doi:10.1038/gene.2010.1.

Ganczak, M., Skonieczna-Zydecka, K., Drozd-Dabrowska, M. and Adler, G. (2017). Possible Impact of 190G >A CCR2 and Δ32 CCR5 Mutations on Decrease of the HBV Vaccine Immunogenicity-A Preliminary Report. *Int J Environ Res Public Health* 14, doi:10.3390/ijerph14020166.

Grzegorzewska, A. E., Jodlowska, E., Mostowska, A., Sowinska, A. and Jagodzinski, P. P. (2014). Single nucleotide polymorphisms of vitamin D binding protein, vitamin D receptor and retinoid X receptor alpha genes and response to hepatitis B vaccination in renal replacement therapy patients. *Expert Rev Vaccines* 13, 1395-403. doi:10.1586/14760584.2014.962521.

Hohler, T., Reuss, E., Freitag, C. M. and Schneider, P. M. (2005). A functional polymorphism in the IL-10 promoter influences the response after vaccination with HBsAg and hepatitis A. *Hepatology* 42, 72-6. doi:10.1002/hep.20740.

Li, Z. K., Nie, J. J., Li, J. and Zhuang, H. (2013). The effect of HLA on immunological response to hepatitis B vaccine in healthy people: a meta-analysis. *Vaccine* 31, 4355-61. doi:10.1016/j.vaccine.2013.06.108.

Nishida, N., Sugiyama, M., Sawai, H., Nishina, S., Sakai, A., Ohashi, J. et al. (2018). Key HLA-DRB1-DQB1 haplotypes and role of the BTNL2 gene for response to a hepatitis B vaccine. *Hepatology* doi:10.1002/hep.29876.

Okada, Y., Uno, N., Sato, S., Mori, S., Sasaki, D., Kaku, N. et al. (2017). Strong influence of human leukocyte antigen-DP variants on response to hepatitis B vaccine in a Japanese population. *Vaccine* 35, 5662-5665. doi:10.1016/j.vaccine.2017.08.045.

Osthoff, M., Irungu, E., Ngure, K., Mugo, N., Thomas, K. K., Baeten, J. M. et al. (2014). Mannose-binding lectin and ficolin-2 do not influence humoral immune response to hepatitis B vaccine. *Vaccine* 32, 4772 - 4777. doi:10.1016/j.vaccine.2014.06.023.

Pan, L., Zhang, W., Liang, Z., Wu, X., Zhu, X., Li, J. et al. (2012). Association between polymorphisms of the cytokine and cytokine receptor genes and immune response to hepatitis B vaccination in a Chinese Han population. *J Med Virol* 84, 26-33. doi:10.1002/jmv.22251.

Pan, L., Zhang, L., Zhang, W., Wu, X., Li, Y., Yan, B. et al. (2014). A genome-wide association study identifies polymorphisms in the HLA-DR region associated with non-response to hepatitis B vaccination in Chinese Han populations. *Hum Mol Genet* 23, 2210-9. doi:10.1093/hmg/ddt586.

Png, E., Thalamuthu, A., Ong, R. T., Snippe, H., Boland, G. J., Seielstad, M. (2011). A genome-wide association study of hepatitis B vaccine response in an Indonesian population reveals multiple independent risk variants in the HLA region. *Hum Mol Genet* 20, 3893-8. doi:10.1093/hmg/ddr302.

Roh, E. Y., Yoon, J. H., In, J. W., Lee, N., Shin, S., Song, E. Y. (2016). Association of HLA-DP variants with the responsiveness to Hepatitis B virus vaccination in Korean Infants. *Vaccine* 34, 2602-7. doi:10.1016/j.vaccine.2016.03.090.

Sakai, A., Noguchi, E., Fukushima, T., Tagawa, M., Iwabuchi, A., Kita, M. et al. (2017). Identification of amino acids in antigen-binding site of class II HLA proteins independently associated with hepatitis B vaccine response. *Vaccine* 35, 703-710. doi:10.1016/j.vaccine.2016.08.068.

Wang, Y., Xu, P., Zhu, D., Zhang, S., Bi, Y., Hu, Y. et al. (2012). Association of polymorphisms of cytokine and TLR-2 genes with long-term immunity to hepatitis B in children vaccinated early in life. *Vaccine* 30, 5708-13. doi:10.1016/j.vaccine.2012.07.010.

Wu, T. W., Chu, C. C., Ho, T. Y., Chang, L. H., Lin, S. K., Lin, M. et al. (2013). Responses to booster hepatitis B vaccination are significantly correlated with genotypes of human leukocyte antigen (HLA)-DPB1 in neonatally vaccinated adolescents. *Hum Genet* 132, 1131-9. doi:10.1007/s00439-013-1320-5.

Wu, T. W., Chen, C. F., Lai, S. K., Lin, H. H., Chu, C. C., Wang, L. Y. (2015). SNP rs7770370 in HLA-DPB1 loci as a major genetic determinant of response to booster hepatitis B vaccination: results of a genome-wide association study. *J Gastroenterol Hepatol* 30, 891-9. doi:10.1111/jgh.12845.

Xu, B., Zhu, D., Bi, Y., Wang, Y., Hu, Y., Zhou, Y. H. (2017). Minimal association of alleles of human leukocyte antigen class II gene and long-term antibody response to hepatitis B vaccine vaccinated during infancy. *Vaccine* 35, 2457-2462. doi:10.1016/j.vaccine.2017.03.021.

Xu, X., Li, Y., Liang, Y., Yin, M., Zhang, Y., Huang, L. et al. (2018). Low responsiveness to a hepatitis B virus vaccine in a Chinese population lacks association with ITGAL, CD58, TNFSF15, CCL15, TGFB3, and BCL6 gene variants. *Infect Genet Evol* 64, 126-130. doi:10.1016/j.meegid.2018.06.010.

Yang, C., Pan, L., Zhang, L., Wu, X., Zhu, X., Yan, B. et al. (2014). BTNL2 associated with the immune response to hepatitis B vaccination in a Chinese Han population. *J Med Virol* 86, 1105-12. doi:10.1002/jmv.23934.

Yoon, J. H., Shin, S., In, J., Chang, J. Y., Song, E. Y., Roh, E. Y. (2014). Association of HLA alleles with the responsiveness to hepatitis B virus vaccination in Korean infants. *Vaccine* 32, 5638-44. doi:10.1016/j.vaccine.2014.08.007.
